# Cost‐effectiveness of population‐based, community, workplace and individual policies for diabetes prevention in the UK

**DOI:** 10.1111/dme.13349

**Published:** 2017-04-18

**Authors:** P. R. Breeze, C. Thomas, H. Squires, A. Brennan, C. Greaves, P. Diggle, E. Brunner, A. Tabak, L. Preston, J. Chilcott

**Affiliations:** ^1^ School of Health and Related Research University of Sheffield Sheffield; ^2^ University of Exeter Medical School University of Exeter Exeter; ^3^ Medical School Lancaster University Lancaster; ^4^ Institute of Infection and Global Health University of Liverpool Liverpool; ^5^ Epidemiology and Public Health University College London London UK; ^6^ First Department of Medicine Semmelweis University Faculty of Medicine Budapest Hungary

## Abstract

**Aim:**

To analyse the cost‐effectiveness of different interventions for Type 2 diabetes prevention within a common framework.

**Methods:**

A micro‐simulation model was developed to evaluate the cost‐effectiveness of a range of diabetes prevention interventions including: (1) soft drinks taxation; (2) retail policy in socially deprived areas; (3) workplace intervention; (4) community‐based intervention; and (5) screening and intensive lifestyle intervention in individuals with high diabetes risk. Within the model, individuals follow metabolic trajectories (for BMI, cholesterol, systolic blood pressure and glycaemia); individuals may develop diabetes, and some may exhibit complications of diabetes and related disorders, including cardiovascular disease, and eventually die. Lifetime healthcare costs, employment costs and quality‐adjusted life‐years are collected for each person.

**Results:**

All interventions generate more life‐years and lifetime quality‐adjusted life‐years and reduce healthcare spending compared with doing nothing. Screening and intensive lifestyle intervention generates greatest lifetime net benefit (£37) but is costly to implement. In comparison, soft drinks taxation or retail policy generate lower net benefit (£11 and £11) but are cost‐saving in a shorter time period, preferentially benefit individuals from deprived backgrounds and reduce employer costs.

**Conclusion:**

The model enables a wide range of diabetes prevention interventions to be evaluated according to cost‐effectiveness, employment and equity impacts over the short and long term, allowing decision‐makers to prioritize policies that maximize the expected benefits, as well as fulfilling other policy targets, such as addressing social inequalities.

## What's new?

- A novel model was developed to help policy‐makers decide which diabetes prevention interventions to pursue, balancing cost‐effectiveness against other objectives, such as equity, employment and short‐term return.
- Most interventions examined were cost‐saving over a lifetime compared with doing nothing.
- Individual‐based intervention in high‐risk individuals is likely to be the most cost‐effective option in the long run, whilst population‐ and community‐based interventions are more equitable, reduce employer costs and are cost‐saving over shorter timescales.
- The model can easily be adapted to evaluate new interventions as they are trialled, and help design local and national diabetes and obesity prevention programmes.

**What's new?**

- A novel model was developed to help policy‐makers decide which diabetes prevention interventions to pursue, balancing cost‐effectiveness against other objectives, such as equity, employment and short‐term return.
- Most interventions examined were cost‐saving over a lifetime compared with doing nothing.
- Individual‐based intervention in high‐risk individuals is likely to be the most cost‐effective option in the long run, whilst population‐ and community‐based interventions are more equitable, reduce employer costs and are cost‐saving over shorter timescales.
- The model can easily be adapted to evaluate new interventions as they are trialled, and help design local and national diabetes and obesity prevention programmes.

## Introduction

Over 35% of adults in England are thought to be at high risk of developing type 2 diabetes because of impaired glucose regulation 1, defined by the American Diabetes Association as HbA_1c_ concentrations of 39–46 mmol/mol (5.7–6.4%). There is now a wealth of evidence that diabetes prevention through lifestyle change for people with impaired glucose regulation is effective 2 and cost‐effective 3, and current National Institute for Health and Care Excellence (NICE) guidelines recommend that individuals at high risk of diabetes [fasting plasma glucose levels 5.5–6.9 mmol/L or HbA_1c_ 42–46 mmol/mol (6.0–6.4%)] are offered an intensive programme of lifestyle change 4. In the UK, a National Diabetes Prevention Programme is being implemented; however, interventions targeting the obesogenic environment may be more cost‐effective, given that the risk factors overlap with other non‐communicable diseases and many people benefit from improvements in diet and lifestyle.

A review identified several diabetes models that have investigated the cost‐effectiveness of diabetes prevention and policies, including intensive lifestyle intervention for individuals at high risk of diabetes, weight loss interventions for obese/overweight individuals and lifestyle promotion through fiscal or media campaigns 5; however, because of differences in model structure it is not possible to compare interventions across studies and no study has directly compared the cost‐effectiveness of intensive lifestyle intervention for individuals at high risk of diabetes within broader weight loss policies in a single modelling framework or estimated how outcomes are distributed across socio‐economic groups.

The aims of the present study were to evaluate the economic benefits of a range of intervention types within a common modelling framework to help prioritize campaigns according to cost‐effectiveness and equity considerations, as well as to report the short‐term cost impact, distribution of outcomes across socio‐economic groups, and implications for work productivity.

## Methods

### Model

The analysis was designed to evaluate lifetime costs and health outcomes of diabetes prevention policies in England. The model was developed using a novel conceptual modelling framework 6, based on literature reviews and in consultation with a stakeholders group of diabetes clinicians, researchers and public health commissioners. The stakeholder group of lay members, clinicians, researchers and public health commissioners met three times to agree the conceptual model, model structure, data inputs and policy interventions. The model was an individual level simulation, written using R software, which allows individual participants to be recruited into interventions conditional on their characteristics. Baseline individual characteristics were obtained from the Health Survey for England 2011, which is a representative sample of the population in England 7. Individuals with diabetes and those aged < 16 years were excluded from analysis. Individuals were sampled at random with replacement from this dataset to populate the model in which 5 000 000 individuals were simulated. In the model, each individual follows trajectories for HbA_1c_, BMI, systolic blood pressure and cholesterol derived from the Whitehall II cohort 8. In yearly cycles, people visit their general practitioner and may be diagnosed with diabetes, hypertension or dyslipidaemia and treated accordingly. The model simulates a number of health outcomes that are related to BMI and diabetes. Each year, individuals are at risk of developing cardiovascular disease (QRISK2 9), heart failure (Framingham study 10), microvascular complications of diabetes (UK Prospective Diabetes Study 11), breast or colon cancer 12, 13, osteoarthritis 14, depression 15, or they may die. A detailed description of the model methods, assumptions, variables and validation tests can be found in the Supporting Information, supplementary methods.

### Healthcare costs and quality of life

Healthcare costs were assigned to the health states in the model to estimate costs from a National Health Service (NHS) and Personal Social Services (PSS) perspective in 2014–2015 UK pounds. It was not feasible to accurately capture all impacts of these interventions from a societal perspective without making substantial assumptions and approximations that would render the final estimate irrelevant. We favour reporting the net benefit from an NHS/PSS perspective, with supplementary information on workplace productivity, to target the analysis at public health and healthcare professionals interested in diabetes prevention. EQ‐5D health questionnaire scores were extracted from the Health Survey for England dataset to describe an individual's baseline health‐related quality of life, and utility decrements were applied in each year of a person's life according to age and health status.

### Work productivity and employer costs

The model was designed to estimate work absence, conditional on health status in employed individuals. The cost to the employer was calculated based on the number of days absent from work. Productivity losses were estimated using the friction cost method, which assumes that there was sufficient unemployment to replace workers on sick leave after a friction period. The employer incurred a recruitment cost of a replacement worker if an individual died whilst employed.

### Interventions

A series of interventions were selected for inclusion in the model (see Supporting Information, supplementary methods for more details). Details of the target population, uptake, effectiveness and costs of the interventions are reported in Table 1.

**Table 1:** Assumptions made to evaluate the effectiveness of interventions

	Soft drinks tax	Retail policy	Workplace	Community	High‐risk individuals
Brief description	20% tax on sugar‐sweetened soft drinks	New supermarket in a deprived urban area	Healthy eating promotion in workplace canteen	Men‐only weight loss programme and cooking skills programme	Individuals attending vascular checks with a Leicester risk score > 4.75 HbA_1_c screening diabetes (HbA_1c_ > 6.5%) and IGR (HbA_1c_ > 6%) individuals with IGR offered lifestyle intervention programme
Total cost per person targeted	None	None	£4.99	£173 (weight‐loss) £82 (Cooking)	HbA_1c_ screening £14 Lifestyle Intervention £280
Population	All individuals	IMD lowest quintile	20% of employed population	IMD lowest quintile and men BMI > 30 kg/m^2^	Screen‐detected IGR
Uptake	100%	100%	11.9% fruit 8.9% milk	11.4%	43.7% vascular checks 32% education uptake
1‐year change in BMI, kg/m^2^	Age 16‐29 −0.23 (−0.28, −0.20)	None	None	Weight loss −1.29 (−1.796, −0.784) Cooking −1.04 (−1.448, −0.632)	−0.94 (−1.265, −0.655)
	Age 30‐49 −0.05 (−0.07, −0.03)				
	Age ≥ 50 0.00 (−0.01, 0.03)				
1‐year change in HbA_1c_ (%)	None	−0.010 (−0.014, −0.006)	Fruit −0.063 (−0.088, −0.034%) Milk −0.0156 (−0.022, −0.009)	Weight loss −0.009 (−0.013, −0.005) Cooking −0.009 (−0.013, −0.005)	−0.121 (−0.215, −0.045)
1‐year change in systolic blood pressure, mm Hg	None	−0.46	Fruit −2.86 (−3.75, −1.67)	Weight loss −0.409 (−0.536, −0.238) Cooking −0.409 (−0.536, −0.238)	−4.30 (−6.11, −2.49)
1‐year change in total cholesterol, mmol/l	None	None	None	None	−0.098 (−0.235, −0.125)
Base case duration of effect	5 years	5 years	5 years	5 years	5 years

IGR, impaired glucose regulation; IMD, index of multiple deprivation.

#### Sugar‐sweetened soft drinks

The effect of a 20% soft drinks tax on mean BMI was estimated previously 16. The age‐dependent effect was applied to the general population. No costs were associated with the soft drinks taxation scheme, nor was revenue included in the NHS and PSS perspective.

#### Retail provision of fruit and vegetables

A supermarket opening was studied to observe the impact of retail provision on local fruit and vegetable consumption 17. Fruit and vegetable consumption increased by 0.162 portions per day after the store opened. The change in fruit and vegetable consumption was related directly to changes in HbA_1c_ and systolic blood pressure. Individuals in the highest index of multiple deprivation (IMD) quintile (low socio‐economic status) received the intervention. The costs were assumed to be incurred by the private sector.

#### Worksite healthy eating promotion

The Heartbeat Award scheme implemented healthy food options in cafeterias in the workplace and observed the impact on workers’ dietary patterns 18. The study reported the proportion of individuals who made a positive switch to healthier food options compared with non‐participating workplaces. The benefits of the workplace intervention were described by the increase in fruit consumption and the switching of milk from a high‐ to a low‐fat choice, and were assumed to affect 20% of the working population.

#### Deprived community education programmes

Two community education programmes were identified to describe the effectiveness of targeted education interventions in deprived communities. Firstly, community nurses in a deprived area of Scotland developed a group‐based weight management intervention specifically for obese men 19. Secondly, a Mediterranean diet class was run for socially deprived women 20. Both studies reported mean change in BMI and change in fruit and vegetable consumption. These interventions were combined such that, within the same scenario, women in the highest IMD quintile were offered a cooking class, whilst men with a BMI > 30 kg/m^2^ and in the highest IMD quintile were offered the diet programme.

#### Translational diabetes prevention programme

An individual's risk of diabetes was assessed using the Leicester Risk Score 21 whilst he or she attended for vascular checks 22, and the individual was invited for diabetes screening if the score was > 4.75. An NHS vascular checks attendance rate of 43.7% was assumed in line with a review of NHS health checks 23 and an intervention uptake rate of 32% based on estimates from Public Health England 24. Individuals who attended screening with HbA_1c_ ≥ 47.5 mmol/mol (6.5%) were diagnosed with diabetes. Individuals with HbA_1c_ ≥ 42.5 mmol/mol (6.0%), and not diagnosed with diabetes were offered the lifestyle programme. A meta‐analysis of translational diabetes prevention programmes was used to estimate the change in BMI, HbA_1c_, systolic blood pressure and cholesterol at 12 months after a lifestyle intervention 22. It was assumed that individuals received 6‐monthly maintenance classes for 3 years after the first year's programme.

#### Maintenance of intervention effectiveness

Data were not available for the maintenance of metabolic changes for each intervention. The effectiveness decreased linearly after the first year, reaching zero effect after 5 years, in line with observations from studies of dietary counselling for weight loss 25.

### Outcomes of the model

The results describe the benefits of the interventions compared with a do‐nothing strategy. Health benefits were measured in quality‐adjusted life years (QALYs). The benefits of the interventions were also described in natural units such as health events. Costs and QALYs were discounted at 1.5% per year. Incremental net benefit was estimated from the NHS/PSS perspective to describe the overall monetary benefit of the interventions in a single unit by assuming a willingness to pay (*λ*) of £20,000 per QALY.

$$\begin{array}{cc} \text{Incremental net benefit}= & \;\lambda *(\text{incremental QALY})-\\ & \left(\text{incremental cost}\right) \end{array}$$

Cost‐effectiveness analysis included a lifetime perspective, in line with NICE guidelines 26. In addition, net benefit over 5 and 10 years was obtained to describe short‐term cost‐effectiveness. The results were disaggregated by deprivation quintiles to enable consideration of the distribution of benefits across socio‐economic groups. Differences in the effectiveness of the interventions did not vary across deprivation quintiles; however, the characteristics and potential to benefit from the interventions is variable. Days absent from work and employer costs are reported separately from the cost‐effectiveness analysis to preserve the NHS/PSS perspective.

One‐way sensitivity analysis was conducted to examine the impact of the intervention assumptions and model variables on the incremental net benefit outcomes. A probabilistic sensitivity analysis was conducted to describe the uncertainty in the model outcomes, for which appropriate statistical distributions were assigned to all uncertain model parameters (Supplementary methods).

## Results

Table 2 shows the lifetime incremental differences between the five interventions and doing nothing in health units and cost‐effectiveness outcomes. The retail policy, workplace intervention and community interventions reduce the number of diabetes diagnoses. The screening and intensive lifestyle intervention for individuals at high risk of diabetes increases the number of diabetes diagnoses over a lifetime by 2102 cases per 5 000 000 in the general population. This is because the screening strategy increases life expectancy and substantially improves identification of diabetes cases that would otherwise remain undiagnosed but are still associated with a high risk of diabetes‐related complications and death.

**Table 2:** Incremental health and cost outcomes of interventions compared with ‘do nothing’ per 5 000 000 simulated individuals in the general population

	Soft drinks tax	Retail policy	Workplace health promotion	Community dietary advice	Intervention for individuals at high risk
Events per 5 000 000 simulated individuals from the general population					
Diabetes diagnosis	−18	−268	−16	−24	2102
Cardiovascular disease	−30	−37	−23	−19	−663
Congestive heart failure	−13	−35	−7	−25	−64
Cardiovascular death	−8	−13	−13	−13	−326
Foot ulcer	3	2	3	1	−551
Amputation	−18	−40	−17	−10	−667
Blindness	−2	−42	6	−5	−1159
Renal failure	−2	−12	−1	−2	−23
Osteoarthritis	−280	−68	−7	−92	−87
Depression	1	0	1	−9	505
Cancer death	17	6	2	7	7
Life years	324	2869	565	167	5571
QALYs^a^	1495	1828	531	372	3301
Mean difference per individual in the general population					
QALYs^a^	0.0003	0.0004	0.0001	0.0001	0.0007
Healthcare costs (lifetime)^a^	−£4.80	−£3.35	−£0.56	£0.00	−£23.85
Net benefit (5 years)^a^	£1.96	£2.18	−£0.10	−£0.67	−£5.09
Net benefit (10 years)^a^	£4.16	£5.55	£0.68	£0.05	−£1.87
**Net benefit (lifetime)** a	£10.78	£10.66	£2.68	£1.48	£37.05

QALY, quality‐adjusted life‐year.

a Discounted at 1.5%.

QALYs valued at £20,000 per QALY for net benefit calculations.

All interventions decrease the incidence of cardiovascular disease, congestive heart disease and cardiovascular death over the lifetime perspective (Table 2). The screening and intensive lifestyle intervention substantially reduces the incidence of microvascular disease and increases the incidence of depression 27. In some cases, other interventions slightly increase the incidence of foot ulcer, amputation and cancer‐specific death because of the increase in life expectancy.

All interventions generate more life‐years and lifetime QALYs compared with a do‐nothing scenario (Table 2). All interventions, except community dietary advice, reduce healthcare spending. The incremental net benefit is a statistic that describes the overall value of each intervention per person in the general population, and is calculated from the costs saved and health benefits valued at £20,000 per QALY. The screening and intensive lifestyle intervention in individuals with a high risk of diabetes generates the greatest incremental net benefit (£37), with a soft drinks taxation coming second in our analysis (£11). The screening and lifestyle intervention has a negative net benefit at 5 and 10 years because it takes longer to re‐coup the costs of the intervention. The retail intervention and soft drinks tax, which incur no costs to the health provider, generate the greatest 5‐year, and 10‐year incremental net benefit. An incremental analysis in Table S1 comparing all intervention strategies found that the screening and intensive lifestyle intervention dominates all other options over a lifetime.

**Figure 1:**
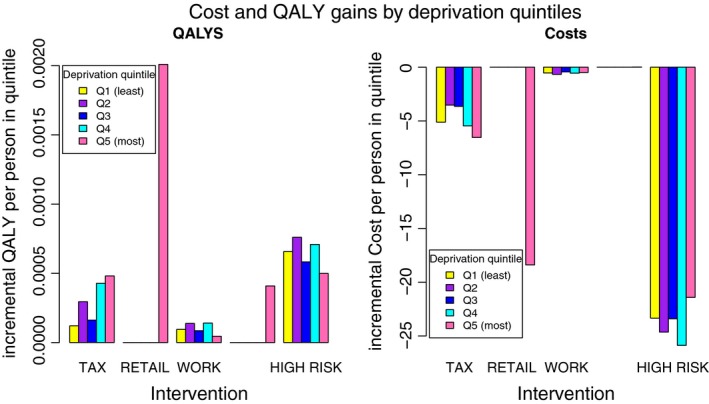
Lifetime incremental costs and QALYs compared to doing nothing, by deprivation quintile from simulation of five million adults in the general population

Figure 1 summarizes the distribution of lifetime QALYs and costs across the five quintiles of deprivation. The retail policy and community interventions generate benefits only in the lowest quintile because these interventions are targeted at the most deprived groups by design. The workplace intervention and intensive lifestyle intervention in individuals at high risk of diabetes have an even spread of benefits across quintiles, whereas the soft drinks taxation offers increased benefits to the most deprived. The interventions do not assume differential effects between socio‐economic groups, therefore, the results are attributable to differences in baseline characteristics and underlying risks of disease progression inherent in the individuals targeted by the interventions. For example, the low socio‐economic group tends to be younger and more likely to be affected by the soft drinks tax.

The wider social impacts of these interventions were considered by looking at the effects on employment (Table 3). The retail, community and intensive lifestyle interventions increase the number of days of work absence over a lifetime compared with doing nothing. The overall impact on the employer costs per individual in employment (56.3% of population) suggests that the soft drinks tax, retail policy, workplace intervention and community interventions generate cost savings for the employer (ranging from £0.01 to £0.12 per individual in employment); however, the screening and intensive lifestyle intervention for individuals at high risk of diabetes is more costly to employers than doing nothing (£0.43 per individual in employment). This counterintuitive result arises because of the increase in the number of individuals with diabetes and, as a consequence, an increase in depression 27, both associated with a high level of work absence; however, to explore uncertainty about this assumption, a sensitivity analysis was performed in which depression and work absence were associated with the onset of diabetes, rather than diagnosis; in this case, the intensive lifestyle intervention saves money for employers (£0.05 per individual in employment).

**Table 3:** Lifetime incremental day of work absence per 5 000 000 simulated individuals, deaths whilst in employment per 5 000 000 simulated individuals and overall employer cost per individual in employment at baseline compared with a do‐nothing strategy

	Soft drinks tax	Retail policy	Workplace health promotion	Community dietary advice	Intervention for individuals at high risk
(a) Baseline analysis assuming work absence after diabetes diagnosis					
Days absent from work	−5118	3181	2102	−854	31044
Deaths whilst in employment	−5	−35	−20	0	−46
Employer cost per individual in employment at baseline^a^	−£0.12	−£0.05	−£0.03	−£0.02	£0.43
(b) Sensitivity analysis assuming work absence after diabetes onset (diagnosed plus undiagnosed)					
Days absent from work	−6106	2253	1473	−321	−919
Deaths whilst in employment	−9	−9	−7	1	−8
Employer cost per individual in employment at baseline^a^	−£0.11	−£0.02	−£0.01	−£0.01	−£0.05

a Discounted at 1.5%.

Table S4 describes the results of the other one‐way sensitivity analyses. These suggest that the results are very sensitive to the rate of weight regain assumed in the model. The education interventions for deprived communities and individuals at high risk of diabetes are also very sensitive to the assumed uptake rates of these interventions. This highlights the importance of recruitment and retention of individuals in education programmes. A sensitivity analysis for HbA_1c_ testing without lifestyle intervention for individuals at high risk results in a net benefit of approximately £25 per person, suggesting that a policy identifying individuals with undiagnosed diabetes alone is also highly cost‐effective. Outcomes for all interventions were sensitive to the discount rate used, but most results were fairly insensitive to non‐intervention model variables. The intervention for individuals at high risk of diabetes, however, was sensitive to diabetes related costs because of the number of individuals diagnosed with diabetes as a result of this screening and intervention process.

**Figure 2:**
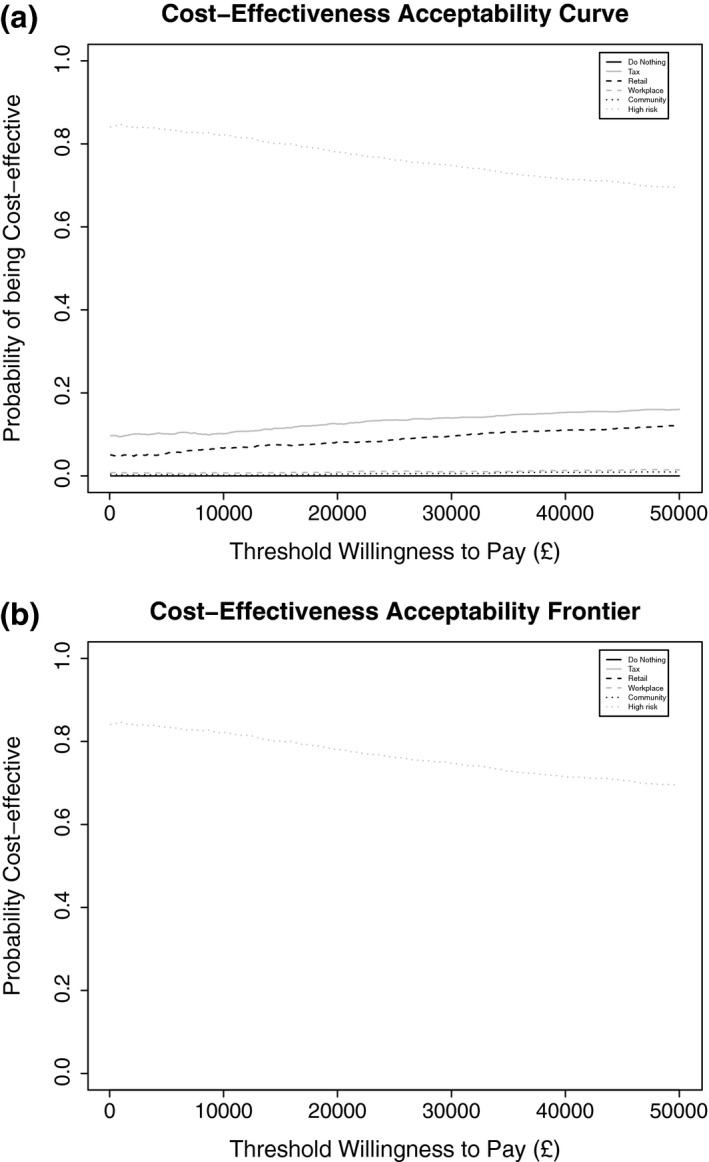
(a) Cost‐effectiveness acceptability curve showing the probability of being cost‐effective of all five interventions and a do‐nothing scenario. (b) Cost‐effectiveness acceptability frontier showing the option maximizing net benefit.

The probabilistic sensitivity analyses are summarized in Fig. 2 and in the Supporting Information, supplementary results. Figure 2a shows that the screening and intensive lifestyle intervention has ~78% probability of being the most cost‐effective strategy above a threshold of £20,000 per QALY gained and Figure 2a shows that this intervention maximizes net benefit at all willingness‐to‐pay thresholds.

## Discussion

The present analysis showed that the intensive lifestyle intervention in individuals at a high risk of diabetes would generate the largest benefits; however, this intervention would not reduce health inequalities in society, and might marginally increase the costs to employers. Furthermore, cost savings take many years to accrue, meaning that, in the short term, the intensive intervention is less cost‐effective to the NHS than the other interventions. By contrast, soft drinks taxation or the retail policy would generate less overall net benefit over a lifetime, but a greater proportion of those who benefit would be in the most deprived groups. These interventions would be marginally cost‐saving for employers and cost‐savings for the NHS would accrue more quickly.

The analysis supports the introduction of two policies currently being implemented in the UK. The NHS Diabetes Prevention Programme will start in 2016 with a first wave of 27 areas making up to 20 000 places available. This will roll out to the whole country by 2020 with an expected 100 000 referrals available each year after. The present analysis shows that the programme will most likely be cost‐saving to the NHS over the lifetime of the patients, and is substantially more cost‐saving than alternative diabetes prevention strategies we have evaluated. In April 2018 a tax will be imposed on sugar‐sweetened drinks in the UK. We have shown that a 20% tax is likely to result in cost‐savings to the NHS and QALY gains and that the benefits are greatest amongst the most deprived socio‐economic groups.

The analysis found that the community intervention in a deprived area would be the least cost‐effective intervention. Sensitivity analysis indicates that the poor performance of this intervention compared with the lifestyle intervention in individuals at high risk of diabetes is attributable to the low uptake of the intervention in this population, and does not capture the benefits of identifying undiagnosed diabetes. It is possible that changes to the choice environment, as demonstrated by the retail intervention or soft drinks tax, may be more cost‐effective in the most deprived communities.

In recent years, several other studies have considered the cost‐effectiveness of multiple interventions targeting different groups within the general population. There are some common findings, for example, that less intensive interventions targeting a broad population are cost‐effective 28, 29 and are more cost‐effective when targeting younger adults 29, and that individual counselling and fiscal measures are more cost‐effective than workplace interventions 28. The present analysis has two major strengths compared with previous diabetes prevention studies 30. Firstly, the flexibility of the model allows input of multiple population‐level scenarios, including a range of different interventions and different population subgroups targeted. Secondly, the breadth of outcomes generated for each intervention, including net benefit to the NHS in the short and long term and impact on socio‐economic groups and employers. This means that the model can be adapted to suit a wide range of potential decision problems that may arise.

The present analysis has shown that most interventions are cost‐saving over the lifetime horizon. In contrast, previous modelling of the health economic consequences of diabetes prevention in the UK suggested that the cost of the intervention would exceed the expected healthcare savings 31, 32. This difference can be attributed to several factors: (1) the cost of diabetes management in early stages of disease is lower in this model following recommendations from clinical experts; (2) the inclusion of renal failure and osteoarthritis generates substantial cost savings for interventions over the long term; and (3) the cost of treating cardiovascular disease has increased.

There are several limitations of the model, many of which arise as a result of assumptions that have had to be made when implementing interventions because of lack of data, for example, concerning rate of weight regain, or because of indirect estimations of intervention efficacy. Sensitivity analysis has been performed to explore these issues and indicates that whereas model results are relatively stable to alteration of general model variables, those concerning the intervention can have dramatic effects on intervention cost‐effectiveness. To reduce uncertainty in such analyses, more evidence is needed on the long‐term duration of benefits, the uptake of interventions in different population sub‐groups and the direct effects of interventions on metabolic trajectories.

Cost‐effectiveness is not the only issue of importance for public health policy‐makers; other targets such as addressing social inequalities are also important 33. We present the relative distribution of incremental costs and QALYs for each intervention (Fig. 1); however, we have not varied the effectiveness of the interventions by sub‐group for the soft drinks tax, workplace intervention or intensive lifestyle programme. It is possible that the interventions will have differential effectiveness according to baseline BMI or socio‐economic group. The evidence suggested almost no differences in effectiveness of the soft drinks tax by income group 16, and no evidence was available to describe the effectiveness of the workplace intervention or intensive lifestyle programme by socio‐economic group or baseline BMI. Further research is needed to examine in more detail the differential impact of these policies on sub‐groups. Our validation work indicates that the model may overestimate HbA_1c_ and systolic blood pressure in people with newly diagnosed diabetes, which may bias the cost‐effectiveness outcomes. There is a paucity of up‐to‐date data, however, on metabolic trajectories for patients with diabetes to investigate whether this underestimate persists in the long term.

There are several avenues for further research to extend the analysis to other policy areas and reduce uncertainty in the model. The model is sufficiently flexible to investigate the effect of layering multiple interventions across overlapping target populations. Another area for future model development would be to add in the effects of changes on physical activity, a common target for diabetes prevention interventions. Unpicking the differential effects of physical activity and dietary change/weight loss would be highly informative to developers of diabetes prevention programmes. Further extensions of the model to describe smoking and alcohol consumption would allow analyses of other public health policies. Finally, we do not currently account for non‐related healthcare costs that may have an impact on the results, particularly where interventions improve survival 34. Current NICE guidelines 26 do not require inclusion of unrelated healthcare costs, but we believe that the model would benefit from inclusion of other health outcomes, such as dementia.

## Funding sources

This study presents independent research funded by the National Institute for Health Research School for Public Health Research. Researchers worked, wrote the report and decided to submit it for publication entirely independently from the funders.

## Competing interests

None declared.

## Supporting information


**Data S1.** Methods
**Data S2.** Results
**Table S1.** Total costs, QALYs and incremental analysis per person in the general population comparing all strategies
**Table S2.** One‐way sensitivity analyses showing expected Net Benefit per individual in the general population (based on 2 million simulated individuals so baseline results differ slightly from Table 1)
**Table S3.** Probabilistic Sensitivity Results for 2000 model runs of 20000 individuals
