# Supplementary material for: Cost‐effectiveness of population‐based, community, workplace and individual policies for diabetes prevention in the UK
Source: Diabet Med. 2017 Apr 18;34(8):1136–44. doi: 10.1111/dme.13349 (PMC5573930; doi:10.1111/dme.13349)

Supplementary Results

The cost-effectiveness and equity impact of population based, community, workplace, and individual policies for weight management and diabetes prevention in the United Kingdom.

Penny R Breeze, Chloe Thomas, Hazel Squires, Alan Brennan, Colin Greaves, Peter Diggle, Eric Brunner, Adam Tabak, Louise Preston, James Chilcott

**Table S1: Total costs, QALYs and incremental analysis per person in the general population comparing all strategies**

| **Intervention** | **Total cost** | **Total QALYs** | **Incremental Costs** | **Incremental QALYs** | **ICER** |
| --- | --- | --- | --- | --- | --- |
| **Intensive Lifestyle Intervention** | £36,348.73 | 15.52665 |  |  | DOMINATED |
| **Soft Drinks Tax** | £36,348.73 | 15.52672 | £0.00 | 0.00007 | DOMINATED |
| **Retail Policy** | £36,348.17 | 15.52676 | -£0.56 | 0.00003 | DOMINATED |
| **Workplace health promotion** | £36,343.92 | 15.52695 | -£4.24 | 0.00019 | DOMINATED |
| **Community dietary advice** | £36,345.38 | 15.52701 | £1.46 | 0.00007 | DOMINATED |
| **Do nothing** | £36,324.88 | 15.52731 | -£20.50 | 0.00029 |  |

Table S2: One-way sensitivity analyses showing expected Net Benefit per individual in the general population (based on 2 million simulated individuals so baseline results differ slightly from Table 1)

|  | **Soft drinks tax** | **Retail Policy** | **Workplace health promotion** | **Community dietary advice** | **Intervention for individuals at high risk** |
| --- | --- | --- | --- | --- | --- |
| **Baseline** | £10.78 | £10.66 | £2.68 | £1.48 | £37.05 |
| Intervention Sensitivity Analyses | | | | | |
| **Intervention Effects 2.5^th^ CI** | £9.44 | £8.51 | £1.08 | £0.37 | £27.92 |
| **Intervention Effects 97.5^th^ CI** | £11.93 | £12.27 | £3.78 | £2.40 | £45.30 |
| **Diabetes screening only** | NA | NA | NA | NA | £25.34 |
| **Intervention Effects last a lifetime (i.e. no weight regain)** | £165 | £112 | NA | NA | NA |
| **Weight regain occurs over 10 years** | NA | NA | £5.29 | £4.49 | £54.40 |
| **Weight regain occurs over 2 years** | NA | NA | -£1.75 | -£1.63 | £25.45 |
| **Switch uptake rates between community dietary advice (11.4%) and intensive lifestyle intervention (43.7%).** | NA | NA | NA | £51.14 | £24.16 |
| **Increase efficacy of workplace intervention by estimating extra effects on BMI and HbA1c due to reducing fried food/sweet puddings.** | NA | NA | £36.67 | NA | NA |
| **Intervention costs +20%** | NA | NA | £2.33 | £1.06 | £36.76 |
| **Intervention cost -20%** | NA | NA | £2.55 | £1.59 | £38.37 |
| Model Parameter Sensitivity Analyses | | | | | |
| **Discount rate at 0%** | £13.79 | £12.61 | £3.44 | £2.17 | £51.09 |
| **Discount rate at 3.5%** | £8.15 | £8.71 | £1.96 | £0.89 | £24.34 |
| **Non-intervention costs at 2.5^th^ CI** | £8.34 | £8.14 | £1.41 | £1.14 | £24.21 |
| **Non-intervention costs at 97.5^th^ CI** | £9.79 | £9.71 | £1.79 | £1.72 | £15.63 |
| **Cardiovascular costs at 2.5^th^ CI** | £11.86 | £11.84 | £2.43 | £2.30 | £74.44 |
| **Cardiovascular costs at 97.5^th^ CI** | £10.69 | £10.58 | £2.02 | £1.96 | £36.97 |
| **Diabetes costs at 2.5^th^ CI** | £10.72 | £10.64 | £2.09 | £2.00 | £37.76 |
| **Diabetes costs at 97.5^th^ CI** | £10.55 | £10.26 | £1.92 | £1.95 | £17.18 |
| **Microvascular costs at 2.5^th^ CI** | £10.97 | £11.20 | £2.27 | £2.03 | £72.04 |
| **Microvascular costs at 97.5^th^ CI** | £10.69 | £10.38 | £2.00 | £1.97 | £35.56 |
| **All utility decrements at 2.5^th^ CI** | £10.76 | £10.91 | £2.12 | £2.00 | £40.00 |
| **All utility decrements 97.5^th^ CI** | £20.74 | £11.95 | £2.30 | £4.05 | £38.39 |
| **Cardiovascular utility decrements at 2.5^th^ CI** | £12.21 | £10.45 | £1.97 | £2.21 | £36.61 |
| **Cardiovascular utility decrements at 97.5^th^ CI** | £10.78 | £10.69 | £2.12 | £2.00 | £37.84 |
| **Microvascular utility decrements at 2.5^th^ CI** | £10.64 | £10.53 | £1.99 | £1.97 | £36.86 |
| **Microvascular utility decrements at 97.5^th^ CI** | £10.76 | £10.67 | £2.08 | £1.99 | £38.36 |
| **Statin uptake 50%** | £10.71 | £10.61 | £2.05 | £1.98 | £37.35 |
| **Statin uptake 80%** | £10.80 | £10.85 | £2.02 | £2.09 | £37.51 |
| **QRISK IGR hazard ratio 2.5^th^ CI** | £10.76 | £10.81 | £2.03 | £2.06 | £37.38 |
| **QRISK IGR hazard ratio 97.5^th^ CI** | £10.97 | £10.40 | £1.22 | £2.01 | £37.45 |
| **No BMI effect on cancer** | £10.11 | £10.26 | £2.35 | £1.11 | £36.52 |
| **No BMI effect on Osteoarthritis** | £3.66 | £9.62 | £2.15 | £-0.04 | £36.12 |
| **No Diabetes or stroke effect on Depression** | £9.19 | £9.71 | £2.14 | £0.90 | £40.38 |
| **No Diabetes impact on mortality** | £9.49 | £9.27 | £1.90 | £1.10 | £44.09 |
| **CVD incidence low** | £8.19 | £8.13 | £1.84 | £0.62 | £42.06 |
| **CVD incidence high** | £14.33 | £11.78 | £3.15 | £2.52 | £22.54 |
| **Low Diabetes diagnosis threshold** | £9.63 | £10.96 | £2.30 | £1.39 | £32.62 |
| **High Diabetes diagnosis threshold** | £9.06 | £10.24 | £2.57 | £1.17 | £47.25 |
| **Low CHF incidence** | £9.58 | £10.52 | £2.45 | £1.31 | £36.31 |
| **High CHF incidence** | £9.08 | £10.25 | £2.34 | £0.94 | £37.31 |
| **Diabetes onset increases risk of depression and work absence** | £11.21 | £12.59 | £2.94 | £1.99 | £37.18 |

**Table S3: Probabilistic Sensitivity Results for 2000 model runs of 20000 individuals**

|  | **Soft drinks tax** | **Retail Policy** | **Workplace health promotion** | **Community dietary advice** | **Intervention for individuals at high risk** |
| --- | --- | --- | --- | --- | --- |
| **Threshold (£ per QALY)** | 20000 | 20000 | 20000 | 20000 | 20000 |
| **Comparator** | Do.Nothing | Do.Nothing | Do.Nothing | Do.Nothing | Do.Nothing |
| **Number of PSA runs** | 2000 | 2000 | 2000 | 2000 | 2000 |
| **Mean inc. Effect per Person (QALY)** | 0.0004 | 0.0004 | 0.00008 | 0.0001 | 0.0008 |
| **Mean inc. Cost per Person (£)** | -5.271 | -4.286 | -0.607 | 0.096 | -28.565 |
| **2.5th CI for inc. Effects (QALY)** | -0.0005 | -0.0001 | -0.0009 | -0.0001 | -0.0007 |
| **97.5th CI for inc. Effects (QALY)** | 0.0019 | 0.0015 | 0.00075 | 0.00076 | 0.0027 |
| **2.5th CI for inc. Costs (£)** | -19.98 | -20.58 | -6.45 | -5.18 | -78.41 |
| **97.5th CI for inc. Costs (£)** | 7.40 | 6.85 | 3.37 | 3.31 | 5.62 |
| **Probability intervention is cost saving** | 0.89 | 0.86 | 0.48 | 0.37 | 0.84 |
| **Probability intervention provides more benefit** | 0.85 | 0.90 | 0.48 | 0.63 | 0.84 |

Figure S1 presents scatter plots to compare the distribution of incremental costs and QALYs between all interventions in the probabilistic sensitivity analysis. The analysis shows that compared with do-nothing all interventions have the majority of probabilistic sensitive analysis outcomes below the threshold. When soft drinks, retail policy, workplace, and community interventions are compared against each other there is generally an even spread across all quadrants indicating that there is very little difference between the cost-effectiveness of the interventions when uncertainty is accounted for. However, the probabilistic sensitivity analysis for lifestyle intervention in high risk individuals confirms that this interventions is likely to be more cost-effective than the other interventions because the PSA runs tend to fall below the threshold.

Figure S1: Multi-way scatter plot for all interventions and a do nothing scenario from 1000 iterations of probabilistic sensitivity analysis.


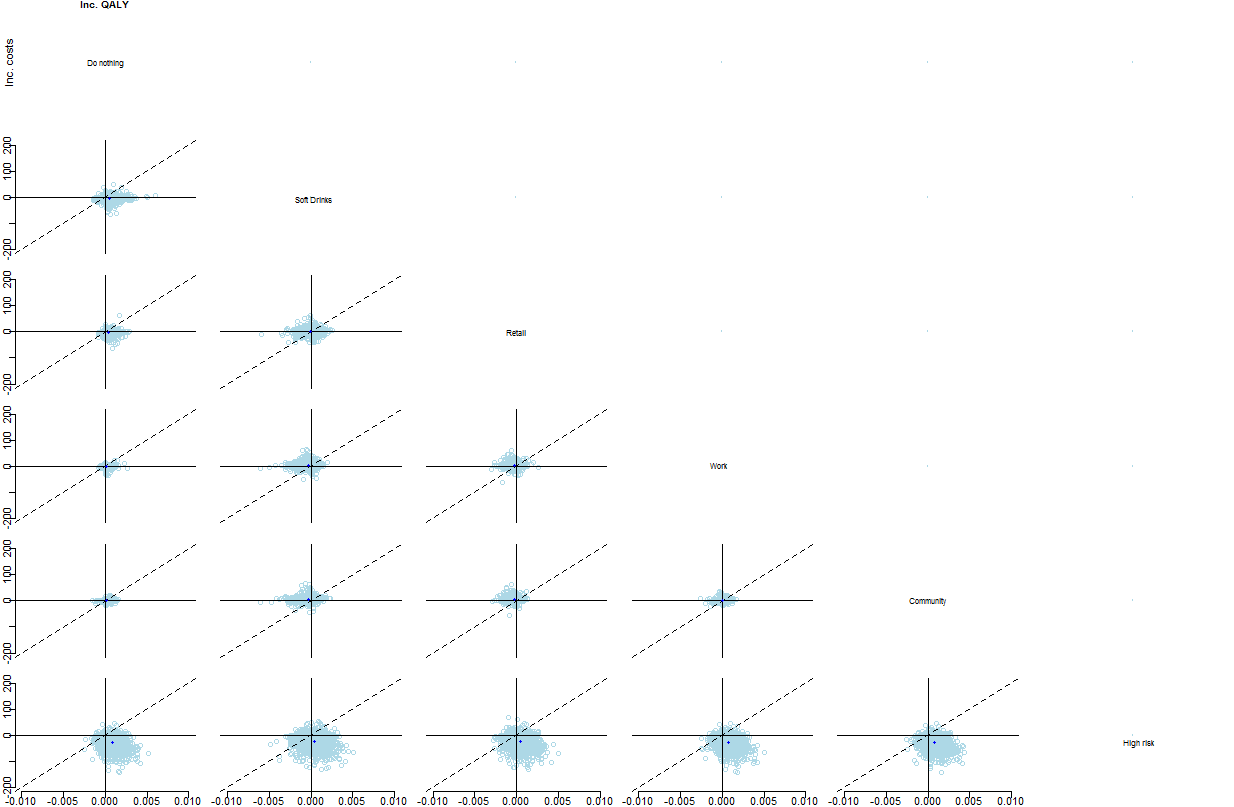

Supplement: Supplementary file 1 — Data S1. Methods Data S2. Results Table S1. Total costs, QALYs and incremental analysis per person in the general population comparing all strategies Table S2. One‐way sensitivity analyses showing expected Net Benefit per individual in the general population (based on 2 million simulated individuals so baseline results differ slightly from Table 1) Table S3. Probabilistic Sensitivity Results for 2000 model runs of 20000 individuals [file DME-34-1136-s001.docx]
