# Supplementary material for: Cost‐effectiveness of population‐based, community, workplace and individual policies for diabetes prevention in the UK
Source: Diabet Med. 2017 Apr 18;34(8):1136–44. doi: 10.1111/dme.13349 (PMC5573930; doi:10.1111/dme.13349)
Supplement: Supplementary file 2 [file DME-34-1136-s002.docx]

School for Public Health Research (SPHR) Diabetes Prevention Model: Detailed Description of Model Background, Methods, Assumptions and Parameters

Penny R Breeze^1^, Chloe Thomas^1^*, Hazel Squires^1^, Alan Brennan^1^, Colin Greaves^2^, Peter J Diggle^3^, Eric Brunner^4^, Adam Tabak^4^, Louise Preston^1^ and James B Chilcott^1^

1. School of Health and Related Research, University of Sheffield
2. Medical School, University of Exeter, United Kingdom
3. Medical School, Lancaster University and Institute of Infection and Global Health, University of Liverpool, United Kingdom
4. Epidemiology & Public Health, University College London, United Kingdom

*Author for correspondence

**ABSTRACT**: Type 2 diabetes is a complex disease with multiple risk factors and health consequences whose prevention is a major public health priority. We have developed a microsimulation model written in the R programming language that can evaluate the effectiveness and cost-effectiveness of a comprehensive range of different diabetes prevention interventions, either in the general population or in subgroups at high risk of diabetes. Within the model individual patients with different risk factors for diabetes follow metabolic trajectories (for body mass index, cholesterol, systolic blood pressure and glycaemia), develop diabetes, complications of diabetes and related disorders including cardiovascular disease and cancer, and eventually die. Lifetime costs and quality-adjusted life-years are collected for each patient. The model allows assessment of the wider social impact on employment and the equity impact of different interventions. Interventions may be population-based, community-based or individually targeted, and administered singly or layered together. The model is fully enabled for probabilistic sensitivity analysis (PSA) to provide an estimate of decision uncertainty. This discussion paper provides a detailed description of the model background, methods and assumptions, together with details of all parameters used in the model, their sources and distributions for PSA.

Contents

[2 Background 9](#_Toc470002013)

[3 Developing the Conceptual Model 10](#_Toc470002014)

[4 Model Structure 12](#_Toc470002015)

[5 Data Selection 15](#_Toc470002016)

[6 Baseline Population Characteristics 16](#_Toc470002017)

[6.1 Choice of Health Survey for England 2011 Dataset 16](#_Toc470002018)

[6.2 Exclusion Criteria 16](#_Toc470002019)

[6.3 Data Extraction 16](#_Toc470002020)

[6.4 Missing data imputation 18](#_Toc470002021)

[6.4.1 Ethnicity 18](#_Toc470002022)

[6.4.2 Anthropometric data 18](#_Toc470002023)

[6.4.3 Metabolic data 21](#_Toc470002024)

[6.4.4 Treatment for Hypertension and Statins 23](#_Toc470002025)

[6.4.5 Gestational Diabetes 23](#_Toc470002026)

[6.4.6 Anxiety/Depression 24](#_Toc470002027)

[6.4.7 Smoking 24](#_Toc470002028)

[6.4.8 Rheumatoid Arthritis and Atrial Fibrillation 24](#_Toc470002029)

[6.4.9 Family history of diabetes 24](#_Toc470002030)

[6.4.10 Economic Activity 24](#_Toc470002031)

[7 GP Attendance in the General Population 26](#_Toc470002032)

[8 Longitudinal Trajectories of Metabolic Risk Factors 28](#_Toc470002033)

[8.1 Whitehall II Data Analysis 28](#_Toc470002034)

[8.2 BMI Trajectory 31](#_Toc470002035)

[8.3 Glycaemic Trajectory in Non-Diabetics/undiagnosed Diabetes 32](#_Toc470002036)

[8.4 HbA1c trajectory in type 2 diagnosed diabetics 34](#_Toc470002037)

[8.5 Total Cholesterol and HDL cholesterol Trajectories in Individuals not receiving Statins 36](#_Toc470002038)

[8.6 Total Cholesterol and HDL cholesterol Trajectories in Individuals receiving Statins 39](#_Toc470002039)

[8.7 Systolic Blood Pressure Trajectories in Individuals not receiving Anti-hypertensive treatment 40](#_Toc470002040)

[8.8 Systolic Blood Pressure Trajectories in Individuals receiving Anti-hypertensive treatment 41](#_Toc470002041)

[8.9 Metabolic Risk factor screening 41](#_Toc470002042)

[8.10 Diagnosis and Treatment Initiation 42](#_Toc470002043)

[9 Comorbid Outcomes and Mortality 44](#_Toc470002044)

[9.1 Cardiovascular Disease 44](#_Toc470002045)

[9.1.1 First Cardiovascular event 44](#_Toc470002046)

[9.1.2 Subsequent Cardiovascular events 47](#_Toc470002047)

[9.1.3 Congestive Heart Failure 49](#_Toc470002048)

[9.2 Microvascular Complications 51](#_Toc470002049)

[9.2.1 Retinopathy 52](#_Toc470002050)

[9.2.2 Neuropathy 52](#_Toc470002051)

[9.2.3 Nephropathy 54](#_Toc470002052)

[9.3 Cancer 55](#_Toc470002053)

[9.3.1 Breast cancer 55](#_Toc470002054)

[9.3.2 Colorectal cancer 56](#_Toc470002055)

[9.4 Osteoarthritis 56](#_Toc470002056)

[9.5 Depression 57](#_Toc470002057)

[9.6 Mortality 58](#_Toc470002058)

[9.6.1 Cardiovascular Mortality 58](#_Toc470002059)

[9.6.2 Cancer Mortality 58](#_Toc470002060)

[10 Direct Health Care Costs 62](#_Toc470002061)

[10.1 GP attendance 62](#_Toc470002062)

[10.2 Diabetes 63](#_Toc470002063)

[10.2.1 Metformin Monotherapy 63](#_Toc470002064)

[10.2.2 Metformin plus Gliptins 64](#_Toc470002065)

[10.2.3 Insulin plus Oral Anti-diabetics 65](#_Toc470002066)

[10.3 Statins 66](#_Toc470002067)

[10.4 Anti-hypertensives 67](#_Toc470002068)

[10.5 Cardiovascular Events 67](#_Toc470002069)

[10.6 Microvascular Events 69](#_Toc470002070)

[10.6.1 Renal Failure 69](#_Toc470002071)

[10.6.2 Foot Ulcers 69](#_Toc470002072)

[10.6.3 Amputation 70](#_Toc470002073)

[10.6.4 Blindness 70](#_Toc470002074)

[10.7 Cancer 70](#_Toc470002075)

[10.8 Osteoarthritis 71](#_Toc470002076)

[10.9 Depression 71](#_Toc470002077)

[11 Employer COsts 73](#_Toc470002078)

[12 Utilities 75](#_Toc470002079)

[12.1 Baseline Utility 75](#_Toc470002080)

[12.2 Utility Decrements 75](#_Toc470002081)

[13 Model Validation 78](#_Toc470002082)

[13.1 Prediction of Diabetes Incidence 78](#_Toc470002083)

[13.1.1 Methods 78](#_Toc470002084)

[13.1.2 Results & Discussion 79](#_Toc470002085)

[13.2 Using Data from HSE 2003 to predict HSE 2011 80](#_Toc470002086)

[13.2.1 Methods 81](#_Toc470002087)

[13.2.2 Results & Discussion 83](#_Toc470002088)

[13.3 Predicting the Results of the ADDITION study 90](#_Toc470002089)

[13.3.1 Methods 90](#_Toc470002090)

[13.3.2 Results & Discussion 91](#_Toc470002091)

[13.4 UKPDS Major Events in Diabetes 92](#_Toc470002092)

[13.4.1 Methods 92](#_Toc470002093)

[13.4.2 Results & Discussion 93](#_Toc470002094)

[14 Diabetes Prevention Interventions 95](#_Toc470002095)

[14.1 Selecting Interventions to be included in the Model 95](#_Toc470002096)

[14.2 Intervention A: Soft-Drinks Tax 96](#_Toc470002097)

[14.2.1 Effectiveness 96](#_Toc470002098)

[14.2.2 Population 98](#_Toc470002099)

[14.2.3 Cost 98](#_Toc470002100)

[14.3 Intervention B: Fruit and Vegetable Retail provision 98](#_Toc470002101)

[14.3.1 Effectiveness 98](#_Toc470002102)

[14.3.2 Population 99](#_Toc470002103)

[14.3.3 Costs 99](#_Toc470002104)

[14.4 Intervention C: Worksite environment 99](#_Toc470002105)

[14.4.1 Effectiveness 99](#_Toc470002106)

[14.4.2 Population 100](#_Toc470002107)

[14.4.3 Costs 101](#_Toc470002108)

[14.5 Intervention D: Community Education Programmes 101](#_Toc470002109)

[14.5.1 Effectiveness 101](#_Toc470002110)

[14.5.2 Population 102](#_Toc470002111)

[14.5.3 Costs 102](#_Toc470002112)

[14.6 Intervention E: Translational Diabetes Prevention Programme 102](#_Toc470002113)

[14.6.1 Effectiveness 102](#_Toc470002114)

[14.6.2 Population 103](#_Toc470002115)

[14.6.3 Costs 103](#_Toc470002116)

[14.7 Maintenance of Intervention Effects 103](#_Toc470002117)

[14.8 Layering Interventions 104](#_Toc470002118)

[15 Probabilistic Sensitivity Analysis 104](#_Toc470002119)

[15.1 GP Attendance in the General Population 104](#_Toc470002120)

[15.2 Whitehall II Statistical Model of Metabolic Trajectories 105](#_Toc470002121)

[15.2.1 HbA1c trajectory in individuals diagnosed with type 2 diabetes 108](#_Toc470002122)

[15.2.2 Systolic blood pressure and cholesterol trajectory following treatment 108](#_Toc470002123)

[15.2.3 Metabolic Risk Factor screening 109](#_Toc470002124)

[15.3 Comorbid Outcomes and Mortality 109](#_Toc470002125)

[15.3.1 Cardiovascular disease 109](#_Toc470002126)

[15.3.2 Congestive Heart Failure 111](#_Toc470002127)

[15.3.3 Microvascular Complications 112](#_Toc470002128)

[15.3.4 Cancer 113](#_Toc470002129)

[15.3.5 Osteoarthritis 113](#_Toc470002130)

[15.3.6 Depression 114](#_Toc470002131)

[15.3.7 Mortality 114](#_Toc470002132)

[15.4 Utilities 114](#_Toc470002133)

[15.5 Unit Health Care Costs 115](#_Toc470002134)

[16 One-Way Sensitivity Analyses 117](#_Toc470002135)

[16.1 Sensitivity Analysis around the Interventions 117](#_Toc470002136)

[16.1.1 Duration of Intervention Effect 118](#_Toc470002137)

[16.1.2 Intervention Uptake 118](#_Toc470002138)

[16.1.3 Intervention Efficacy 118](#_Toc470002139)

[16.2 Sensitivity Analysis around other Parameters 119](#_Toc470002140)

[17 Model Limitations and Further Research 121](#_Toc470002141)

[17.1 Model Limitations 121](#_Toc470002142)

[17.2 Further Research 122](#_Toc470002143)

# Background

This project aims to provide a coherent framework for the evaluation of strategies for the prevention of type 2 diabetes. Specifically, the focus is to enable the design of preventive strategies that are effective and cost-effective in combination, and support decision making around these. This is to enable a rational allocation of resources between population/community level interventions, which aim to alter the distribution of risk factors for diabetes, and targeted identification/screening interventions such as the NHS health checks programme, which aim to identify and provide management for individuals at increased risk.

There are a number of necessary steps involved in translating knowledge from epidemiological studies in diabetes into preventive action: Identification of individuals or groups who are at increased risk; description of the important risk factors that can be altered; identification of key influences on risk factors (e.g. attitudes or environmental aspects); development of interventions to act on risk factors (e.g. promoting walking); development of methods to identify people likely to benefit from an intervention; evaluation of the success of an intervention and estimation of its potential impact on public health.

Modelling can play a key role in developing our understanding of this complex system. Firstly it can estimate the potential impact of different risk identification and management strategies on public health outcomes and help in pinpointing the most cost-effective strategies for intervention. Furthermore, it can play a key role in facilitating the iterative research cycle by helping us identify and analyse key current uncertainties, focus further research and input into the design of the next generation of interventions.

As part of this project we conducted a review of previous decision analytic models used to evaluation diabetes prevention interventions (1). This review confirmed that no other diabetes models were sufficient to meet the objectives of this project and identified some areas of development from previous models to consider in the model design.

# Developing the Conceptual Model

A conceptual model of the problem and a model-based conceptual model were developed according to a new conceptual modelling framework for complex public health models (2). In line with this framework the conceptual models were developed in collaboration with a project stakeholder group comprising health economists, public health specialists, research collaborators from other SPHR groups, diabetologists, local commissioners and lay members. The conceptual model of the problem mapped out all relevant factors associated with diabetes based upon iterative literature searches. Key initial sources were reports of two existing diabetes prevention models used for National Institute for Health and Care Excellence public health guidance (3;4). This conceptual model of the problem was presented at a Stakeholder Workshop. Discussion at the workshop led to modifications of the model, identifying additional outcomes such as depression and helping to identify a suitable conceptual model boundary for the cost-effectiveness model structure. Table 1 describes which factors included within the conceptual model of the problem were chosen to be included and excluded from the health economic model as agreed by stakeholders following the workshop. This final model boundary based upon Table 1 provided the final scope for the simulation model developed. A review of previous economic evaluations of diabetes prevention was also instrumental in deciding on the final boundary of the economic model (1).

Table 1: Diabetes model boundary selection

| **Factor** | **Include/Exclude** | **Reason for inclusion/exclusion** |
| --- | --- | --- |
| Risk factors | Include | Key component of causal diagram |
| Gestational diabetes/ pregnancy complications | Exclude | This is a small subgroup and is not considered to be a focus of this project. |
| Osteoarthritis | Include | Diabetes has been found to be an independent risk factor for osteoarthritis independent of the effect of BMI (5). In addition, the report by Gillett et al. suggests that the cost of osteoarthritis is comparable to the cost of diabetes (6). |
| Risk factors of next generation | Exclude | Within the high risk group, only a minority of people will parent a young child due to the age of the people affected, thus there would be limited impact upon the next generation. Within the general population, Whitaker et al. suggest that parental obesity more than doubles the risk of adult obesity among their children. This could bear substantial future costs and effects; however because these costs and outcomes would occur so far in the future, by applying a discount rate to both costs and effects, there would be minimal impact upon the model results. Time would be better spent elsewhere. |
| Blood glucose levels/ Non-diabetic hyperglycaemia/ Diabetes | Include | Key component of causal diagram. |
| Hypoglycaemia & weight gain associated with pharmacological interventions | Include (but not as a separate factor) | The quality of life implications of hypoglycaemia and weight gain are likely to be captured within the quality of life of people with diabetes. There are likely to be minimal additional costs associated with hypoglycaemia and weight gain above those associated with treating the disease. |
| Non-alcoholic fatty liver | Include (but not as a separate factor) | This is likely to be included within the costs and quality of life estimates associated with diabetes and obesity. |
| Fatigue | Include (but not as a separate factor) | The quality of life implications of fatigue are likely to be captured within the quality of life of people with disease. There are likely to be minimal additional costs associated with fatigue above those associated with treating disease. |
| Neuropathy | Include | Key outcome associated with diabetes. |
| Erectile dysfunction | Include (but not as a separate factor) | This is likely to be included within the costs and quality of life impacts of neuropathy. |
| Nephropathy | Include | Key outcome associated with diabetes. |
| Retinopathy | Include | Key outcome associated with diabetes |
| Cancers (post-menopausal breast cancer, colorectal cancer) | Include | The report by the World Cancer Research Fund (WCRF) Panel on Food, Nutrition, Physical Activity and the Prevention of Cancer suggests that BMI has a significant impact upon the incidence and mortality of post-menopausal breast cancer, colorectal cancer, oesophagus cancer, kidney cancer, endometrial cancer, gall bladder cancer and pancreatic cancer (7). It also suggests that physical activity is associated with colorectal cancer, postmenopausal breast cancer and endometrial cancer. |
| CVD including hypertension, coronary heart disease (leading to heart attacks & angina), congestive heart failure, and cerebrovascular disease (incl. stroke & dementia) | Include | Has a substantial impact upon both costs and effects. |
| Mental illness (incl. dementia) | Partially include (but not as a separate factor for all illnesses) | Depression was included as a separate factor. However, the relationship between mental illness and diabetes is complex and currently not completely understood. Part of the relationship is associated with the incidence of cerebrovascular disease and the impact of mental illness will be captured within these costs and outcomes. The remaining associations, such as the direct increase in mental illness as a result of being diagnosed and living with diabetes, are difficult to untangle and are expected to have a small impact upon the model outcomes relative to other model factors. |
| Obstructive sleep apnoea | Include (but not as a separate factor) | The relationship between risk factors and CVD is expected to capture those events resulting from obstructive sleep apnoea. The quality of life associated with people who are overweight is likely to include poorer quality of life resulting from obstructive sleep apnoea. In the instances where sleep apnoea is treated, the cost is minimal. |
| Infectious diseases | Exclude | Relative to other model factors, this is likely to have a smaller impact upon the model outcomes. |
| Environmental outcomes (congestion, CO2, pollutants) | Not currently clear | This depends upon the choice of interventions within the model (see Section 1). |

# Model Structure

We developed an individual patient simulation that estimates individuals’ health in yearly cycles until death. Figure 1 describes the overall model structure and indicates the interactions between constant, time-varying and health events in the model.

Figure 1: Model structure and health state interactions


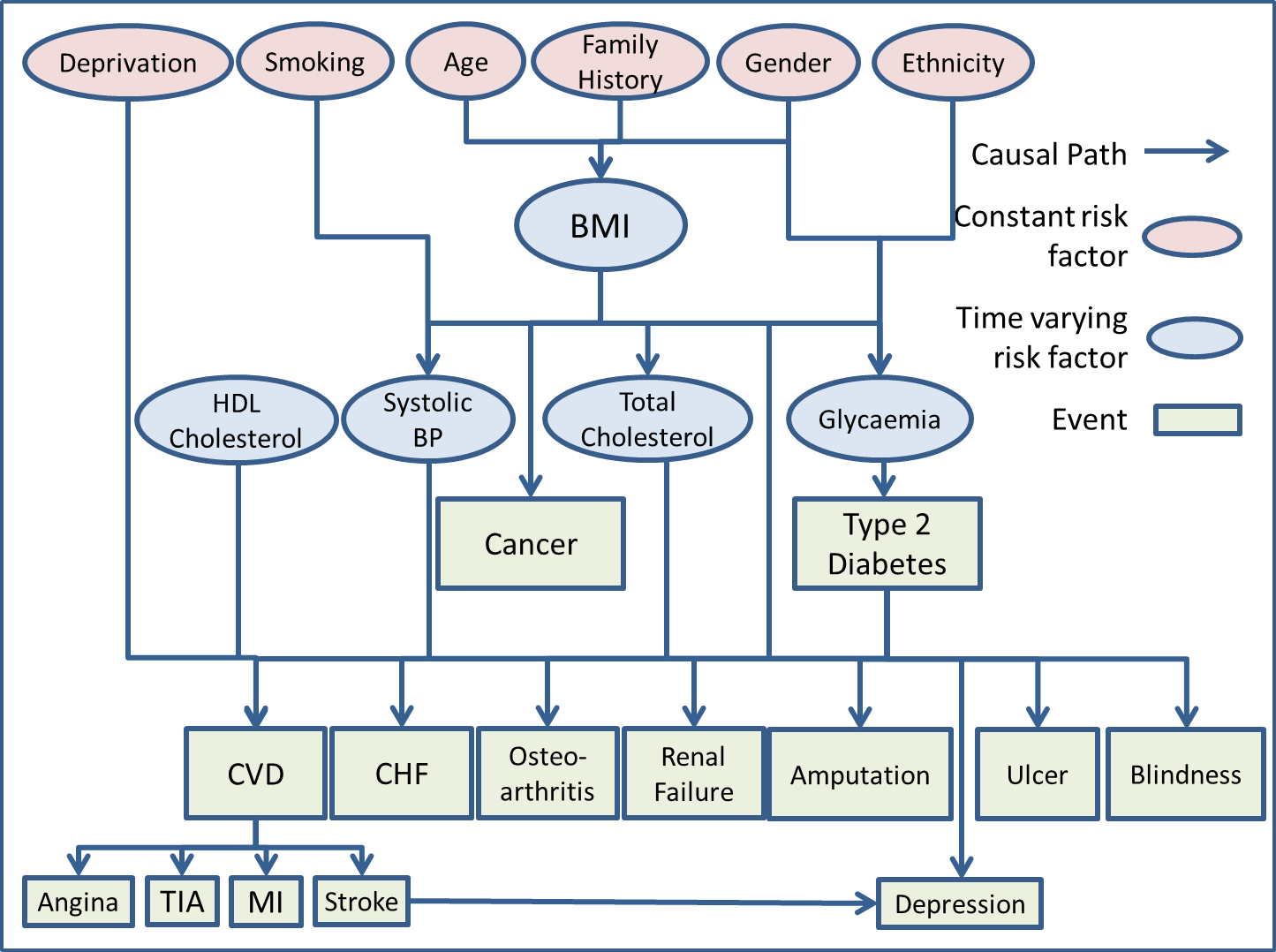


The simulation draws baseline demographic and clinical status for individuals sampled from the Health Survey for England (HSE) 2011 (8). The simulation estimates yearly changes in metabolic risk factors based upon the individuals’ baseline characteristics. Within each annual cycle the individuals may be screened for hypertension, dyslipidaemia or diabetes during a visit to the GP. The opportunistic screening is used to determine diabetes diagnosis or the initiation of anti-hypertensive treatment or statins. Baseline characteristics and metabolic risk factors determine the individuals’ probability of cardiovascular events, diabetes microvascular complications, cancer, osteoarthritis and depression. Individuals within the model may die in any cycle as a result of cardiovascular disease, cancer or from other causes.

Figure 1 illustrates the sequence of updating clinical characteristics and clinical events that are estimated within a cycle of the model. This sequence is repeated for every annual cycle of the model. The first stage of the sequence updates the age of the individual. The second stage estimates how many times the individual attends the GP. The third stage estimates the change in BMI of the individual from the previous period. In the fourth stage, if the individual has not been diagnosed as diabetic (Diabetes_Dx=0) their change in glycaemia is estimated using the Whitehall II model. If they are diabetic (Diabetes_Dx=1), it is estimated using the UKPDS model. In stages five and six the individual’s blood pressure and cholesterol are updated using the Whitehall II model if the individual is not identified as hypertensive or receiving statins. In stage seven, the individual may undergo assessment for diabetes, hypertension and dyslipidaemia during a GP consultation. From stage eight onwards the individual may experience cardiovascular outcomes, diabetes related complications, cancer, osteoarthritis or depression. If the individual has a history of cardiovascular disease (CVD history=1), they follow a different pathway in stage eight to those without a history of cardiovascular disease (CVD history=0). Individuals with HbA1c greater than 6.5 are assumed to be at risk of diabetes related complications. Individuals who do not have a history of cancer (Cancer history=0) are at risk of cancer diagnosis, whereas those with a diagnosis of cancer (Cancer history=1) are at risk of mortality due to cancer. Individuals without a history of osteoarthritis or depression may develop these conditions in stages 12 and 13. Finally, all individuals are at risk of dying due to causes other than cardiovascular or cancer mortality. Death from renal disease is included in the estimate of other-cause mortality.

Figure 2: Model Schematic

# Data Selection

Having developed and agreed the model structure and boundary with the stakeholder group the project team sought suitable sources of data for the baseline population, GP attendance, metabolic risk trajectories, treatment algorithms, and risk models for long term health outcomes, health care and health related. Given the complexity of the model it was not possible to use systematic review methods to identify all sources of data for these model inputs. As a consequence we used a series of methods to identify the most appropriate sources of data within the time constraints of the project.

Firstly, we discussed data sources with the stakeholder groups and identified key studies in the UK that have been used to investigate diabetes and its complications and comorbidities. The stakeholder group included experts in the epidemiology of non-communicable disease who provided useful insight into the strengths and limitations of prominent cohort studies and trials that have studies the risks of long term health outcomes included in the model. The stakeholder group included diabetes prevention cost-effectiveness modellers, whose understanding of studies that could be used to inform risk parameters, costs and health related quality of life estimates. Secondly, we used a review of economic evaluations of diabetes prevention and weight management cost-effectiveness studies to identify sources of data used in similar economic evaluations (1). Thirdly, we conducted targeted literature searches where data could not be identified from large scale studies of a UK population, or could be arguably described as representative of a UK population through processes described above. Justification for data inputs for all model parameters are described below.

# Baseline Population Characteristics

## Choice of Health Survey for England 2011 Dataset

The model required demographic, anthropometric and metabolic characteristics that would be representative of the UK general population. The Heath Survey for England (HSE) was suggested by the stakeholder group because it collects up-to-date cross-sectional data on the characteristics of all ages of the English population. It also benefits from being a reasonably good representation of the socioeconomic profile of England. A major advantage of this dataset is that includes important clinical risk factors such as HbA1c, SBP, and cholesterol. The characteristics of individuals included in the cost-effectiveness model were based sampled from the HSE 2011 dataset (8). The HSE 2011 focused on CVD and associated risk factors. The whole dataset was obtained from the UK Data Service.

## Exclusion Criteria

The total sample size of the HSE 2011 was 10,617. Individuals from the HSE dataset who met the following criteria were excluded from the sample. The list of exclusion criteria and the number of individuals that met these criteria are listed below.

1. Individuals younger than 16 years (N=2007)
2. Individuals with a previous diagnosis of diabetes (N=572)

This left a final sample size of 8038 individuals.

## Data Extraction

Only a subset of variables reported in the HSE 2011 cohort was needed to inform the baseline characteristics in the economic model. A list of model baseline characteristics and the corresponding variable name and description from the HSE 2011 are listed below in Table 2. Two questions for smoking were combined to describe smoking status according to the QRISK2 algorithm in which former smokers and the intensity of smoking are recorded within one measure. The number of missing data for each observation in the HSE data is detailed in Table 2 and summary statistics for the data extracted from the HSE2011 dataset are reported in Table 3.

Table 2: HSE variable names and missing data summary

| **Model requirements** | **HSE 2011 variable name** | **HSE 2011 variable description** | **No. Missing data entries (N=8038)** |
| --- | --- | --- | --- |
| Age | Age | Age last birthday | 0 |
| Sex | Sex | Sex | 0 |
| Ethnicity | Origin | Ethnic origin of individual | 36 |
| Deprivation (Townsend) | qimd | Quintile of IMD SCORE | 0 |
| Weight | wtval | Valid weight (Kg) inc. estimated>130kg | 1284 |
| Height | htval | Valid height (cm) | 1207 |
| BMI | bmival | Valid BMI | 1431 |
| Waist circumference | wstval | Valid Mean Waist (cm) | 2871 |
| Waist-Hip ratio | whval | Valid Mean Waist/Hip ratio | 2882 |
| Total Cholesterol | cholval | Valid Total Cholesterol Result | 4760 |
| HDL cholesterol | hdlval | Valid HDL Cholesterol Result | 4760 |
| HbA1c | glyhbval | Valid Glycated HB Result | 4360 |
| FPG |  |  | N/A |
| 2-hr glucose |  |  | N/A |
| Systolic Blood pressure | omsysval | Omron Valid Mean Systolic BP | 3593 |
| Hypertension treatment | medcinbp | Currently taking any medicines, tablets or pills for high BP | 6050 |
| Gestational diabetes | pregdi | Whether pregnant when told had diabetes | 8008 |
| Anxiety/depression | Anxiety | Anxiety/Depression | 930 |
| Smoking | cigsta3 | Cigarette Smoking Status: Current/Ex-Reg/Never-Reg | 75 |
|  | cigst2 | Cigarette Smoking Status - Banded current smokers | 74 |
| Statins | lipid | Lipid lowering (Cholesterol/Fibrinogen) – prescribed | 5804 |
| Rheumatoid Arthritis | compm12 | XIII Musculoskeletal system | 5 |
| Atrial Fibrillation | murmur1 | Doctor diagnosed heart murmur (excluding pregnant) | 2008 |
| Family history diabetes |  |  | N/A |
| History of Cardiovascular disease | cvdis2 | Had CVD (Angina, Heart Attack or Stroke) | 3 |
| Economic Activity | econact | Economic status | 37 |

Table 3: Characteristics of final sample from HSE 2011 (N=8038)

|  | **Number** | **Percentage** |  |
| --- | --- | --- | --- |
| Male | 3506 | 43.6 |  |
| White | 7212 | 89.7 |  |
| IMD 1 (least deprived) | 1700 | 21.1 |  |
| IMD 2 | 1699 | 21.1 |  |
| IMD 3 | 1696 | 21.1 |  |
| IMD 4 | 1479 | 18.4 |  |
| IMD 5 (most deprived) | 1464 | 18.2 |  |
| Non-smoker | 6415 | 79.8 |  |
| Anti-hypertensive treatment | 2092 | 26.0 |  |
| Statins | 665 | 8.3 |  |
| Employed | 4525 | 56.3 |  |
| Unemployed | 385 | 4.8 |  |
| Retired | 1945 | 24.2 |  |
| Economically Inactive | 1183 | 14.7 |  |
|  | **Mean** | **Standard deviation** | **Median** |
| Age | 48.59 | 18.49 | 47.00 |
| BMI | 27.13 | 5.18 | 26.40 |
| Total Cholesterol | 5.42 | 1.07 | 5.40 |
| HDL Cholesterol | 1.53 | 0.44 | 1.50 |
| HbA1c | 5.61 | 0.47 | 5.60 |
| Systolic Blood Pressure | 125.90 | 16.92 | 123.50 |
| EQ-5D (TTO) | 0.836 | 0.232 | 0.883 |
| BMI Body Mass Index; IMD Index of Multiple Deprivation; EQ-5D 5 dimensions Euroqol (health related quality of life index) | | | |

A complete dataset was required for all individuals at baseline. However, no measurements for Fasting Plasma Glucose (FPG) or 2 hour glucose were obtained for the HSE 2011 cohort. In addition, the questionnaire did not collect information about individual family history of diabetes or family history of Cardiovascular Disease (CVD). These variables were imputed from other datasets.

Many individuals were lacking responses to some questions but had data for others. One way of dealing with this is to exclude all individuals with incomplete data from the sample. However, this would have reduced the sample size dramatically, which would have been detrimental to the analysis. It was decided that it would be better to make use of all the data available to represent a broad range of individuals within the UK population. With this in mind, we decided to use assumptions and imputation models to estimate missing data.

## Missing data imputation

### Ethnicity

Only a small number of individuals had missing data for ethnicity. In the QRISK2 algorithm the indicator for white includes individuals for whom ethnicity is not recorded. In order to be consistent with the QRISK2 algorithm we assumed that individuals with missing ethnicity data were white.

### Anthropometric data

A large proportion of anthropometric data was missing in the cohort. Table 4 reports the number of individuals with two or more anthropometric records missing. This illustrates that only 758 individuals had no anthropometric data at all. Imputation models for anthropometric data were developed utilising observations from other measures to help improve their accuracy.

Table 4: Multi-way assessment of missing data

| Conditions | Number of individuals |
| --- | --- |
| No weight and no height | 1060 |
| No weight and no waist circumference | 907 |
| No weight and no hip circumference | 906 |
| No height and no waist circumference | 818 |
| No height and no hip circumference | 817 |
| No hip and no waist | 2865 |
| No anthropometric data | 758 |

Data were imputed using linear regression models to describe patterns observed within the dataset. Simple ordinary least squares (OLS) regression models were used to predict missing data. Missing data were sampled stochastically from the conditional distributions to allow variability in imputed values.

Two imputation models were generated for each of the following anthropometric measures: weight, height, waist circumference and hip circumference. The first imputation method included an alternative anthropometric measure to improve precision. The second included only age and/or sex, to be used if the alternative measure was also missing. Simple ordinary least squares (OLS) regression models were used to predict missing data. Summary data for each measure confirmed that the data were approximately normally distributed. Covariate selection was made by selecting the anthropometric measure that maximised the Adjusted R-squared statistic, and age and sex were included if the coefficients were statistically significant (P<0.1).

The imputation models for weight are reported in Table 5. Individuals’ sex and age were included in both models. A quadratic relationship between age and weight was identified. Waist circumference had a positive and significant relationship with weight. The R^2^ for model 1 suggested that 80% of the variation in weight is described by the model. The R^2^ for model 2 was much lower as only 18% of the variation in weight was described by age and sex. The residual standard error is reported for both models.

**Table 5: Imputation model for weight**

| **Coefficient** | **Model 1** | **Model 2** |
| --- | --- | --- |
| Intercept | -17.76 | 50.249 |
| Sex | 2.614 | 13.036 |
| Age | 0.064 | 0.903 |
| Age*Age | -0.0027 | -0.0086 |
| Waist circumference | 1.060 |  |
| R-squared | 0.7981 | 0.1831 |
| Residual standard error | 7.483 | 15.31 |

The imputation models for height are reported in Table 6. Individuals’ sex and age were included in both models. A quadratic relationship between age and height was identified. Waist circumference had a positive and significant relationship with height. The R^2^ for model 1 suggested that 53% of the variation in height is described by the model suggesting a fairly good fit. The R^2^ for model 2 was slightly lower in which 52% of the variation in height was described by age and sex. The residual standard error is reported for both models.

**Table 6: Imputation model for height**

| **Coefficient** | **Model 1** | **Model 2** |
| --- | --- | --- |
| Intercept | 157.4 | 162.1 |
| Sex | 12.82 | 13.43 |
| Age | 0.081 | 0.1291 |
| Age*Age | -0.0021 | -0.0025 |
| Waist circumference | 0.071 |  |
| R-squared | 0.532 | 0.5244 |
| Residual standard error | 6.617 | 6.682 |

The imputation models for waist circumference are reported in Table 7. Individuals’ sex and age were included in both models. A quadratic relationship between age and waist circumference fit to the data better than a linear relationship. Weight had a positive and significant relationship with waist circumference. The R^2^ for model 1 suggested that 81% of the variation in waist circumference is described by the model suggesting a very good fit. The R^2^ for model 2 was much lower in which only 22% of the variation in waist circumference was described by age and sex which is a moderately poor fit. The residual standard error is reported for both models.

**Table 7: Imputation model for waist**

| **Coefficient** | **Model 1** | **Model 2** |
| --- | --- | --- |
| Intercept | 28.73 | 65.327 |
| Sex | 0.5754 | 9.569 |
| Age | 0.1404 | 0.7617 |
| Age*Age | 0.0007 | -0.0053 |
| Weight | 0.7098 |  |
| R-squared | 0.8096 | 0.2196 |
| Residual standard error | 6.122 | 12.44 |

The imputation models for hip circumference are reported in Table 8. Individuals’ sex and age were included in both models. A quadratic relationship between age and hip circumference fit to the data better than a linear relationship. Weight had a positive and significant relationship with hip circumference. The R^2^ for model 1 suggested that 80% of the variation in hip circumference is described by the model suggesting a very good fit. The R^2^ for model 2 was much lower in which only 2% of the variation in hip circumference was described by age and sex which is a very poor fit. The residual standard error is reported for both models.

**Table 8: Imputation model for hip**

| **Coefficient** | **Model 1** | **Model 2** |
| --- | --- | --- |
| Intercept | 66.9145 | 96.891 |
| Sex | -8.3709 | -0.9783 |
| Age | -0.1714 | 0.3528 |
| Age*Age | 0.0021 | -0.0029 |
| Weight | 0.5866 |  |
| R-squared | 0.7949 | 0.023 |
| Residual standard error | 4.539 | 10.1 |

### Metabolic data

A large proportion of metabolic data was missing in the cohort, ranging from 2997-4309 observations for each metabolic measurement. Table 9 reports the number of individuals with two or more metabolic records missing. This illustrates that 2987 individuals have no metabolic data. Imputation models for metabolic data were developed utilising observations from other measures to help improve their accuracy.

Table 9: Multi-way assessment of missing data

| **Conditions** | **Number of individuals** |
| --- | --- |
| No HbA1c and no cholesterol | 4309 |
| No HbA1c and no blood pressure | 2997 |
| No cholesterol and no blood pressure | 3050 |
| No metabolic data | 2987 |

Two imputation models were generated for each of the following metabolic measures: total cholesterol, high density lipoprotein (HDL) cholesterol, HbA1c and systolic blood pressure (SBP) and. The first imputation method included an alternative metabolic measure to improve precision. The second included only age and/or sex, to be used if the alternative measure was also missing. Simple ordinary least squares (OLS) regression models were used to predict missing data. Summary data for each measure confirmed that the data were approximately normally distributed. Covariate selection was made by selecting the metabolic measure that maximised the adjusted R-squared statistic, and age and sex were included if the coefficients were statistically significant (P<0.1).

These imputation models were developed to estimate metabolic data from information collected in the HSE. An alternative approach would have been to use estimates of these measures from the natural history statistical models. At the time of the analysis it was uncertain what form and design the natural history models would take, therefore the HSE imputation models were developed for use until a better alternative was found.

The imputation models for total cholesterol are reported in Table 10. Individuals’ age was included in both models. A quadratic relationship between age and weight was identified. Diastolic blood pressure had a positive and significant relationship with total cholesterol. The R^2^ for model 1 suggested that 20% of the variation in total cholesterol is described by the model. The R^2^ for model 2 was lower in which only 18% of the variation in total cholesterol was described by age. The residual standard error is reported for both models.

**Table 10: Imputation model for total cholesterol**

| **Coefficient** | **Model 1** | **Model 2** |
| --- | --- | --- |
| Intercept | 1.973 | 2.821 |
| Age | 0.0774 | 0.0904 |
| Age*Age | -0.0006 | -0.0007 |
| Diastolic blood pressure | 0.0159 |  |
| R-squared | 0.2035 | 0.1792 |
| Residual standard error | 0.9526 | 0.9741 |

The imputation models for HDL cholesterol are reported in Table 11. Individuals’ sex and age were included in both models. A quadratic relationship between age and height was identified. Diastolic blood pressure had a negative and significant relationship with HDL cholesterol. The R^2^ for model 1 suggested that only 13% of the variation in HDL cholesterol is described by the model suggesting a relatively poor fit. The R^2^ for model 2 suggested that 12% of the variation in HDL cholesterol was described by age and sex. The residual standard error is reported for both models.

**Table 11: Imputation model for HDL Cholesterol**

| **Coefficient** | **Model 1** | **Model 2** |
| --- | --- | --- |
| Intercept | 1.501 | 1.383 |
| Sex | -0.279 | -0.274 |
| Age | 0.0086 | 0.0075 |
| Age*Age | -0.0001 | -0.00004 |
| Diastolic blood pressure | -0.0018 |  |
| R-squared | 0.1198 | 0.1157 |
| Residual standard error | 0.4122 | 0.417 |

The imputation models for HbA1c are reported in Table 12. Individuals’ age was included in both models. A quadratic relationship between age and HbA1c fit to the data better than a linear relationship. SBP had a positive and significant relationship with HbA1c. The R^2^ for model 1 suggested that only 19% of the variation in HbA1c is described by the model, suggesting a modest fit. The R^2^ for model 2 described 18% of the variation in HbA1c by age alone. The residual standard error is reported for both models.

**Table 12: Imputation model for HbA1c**

| **Coefficient** | **Model 1** | **Model 2** |
| --- | --- | --- |
| Intercept | 4.732 | 4.962 |
| Age | 0.0141 | 1.422 |
| Age*Age | -0.00003 | -0.00003 |
| Systolic blood pressure | 0.002 |  |
| R-squared | 0.1941 | 0.1835 |
| Residual standard error | 0.4243 | 0.4228 |

The imputation models for SBP are reported in Table 13. Individuals’ sex and age were included in both models. A linear relationship between age and SBP fit to the data better than a quadratic relationship. Total cholesterol and HbA1c had a positive and significant relationship with SBP, whereas HDL cholesterol had a negative significant relationship with SBP. The R^2^ for model 1 suggested that 22% of the variation in SBP is described by the model suggesting a modest fit. The R^2^ for model 2 was similar in which only 20% of the variation in SBP was described by age and sex. The residual standard error is reported for both models.

**Table 13: Imputation model for Systolic Blood Pressure**

| **Coefficient** | **Model 1** | **Model 2** |
| --- | --- | --- |
| Intercept | 84.983 | 104.132 |
| Sex | 6.982 | 6.396 |
| Age | 0.330 | 0.380 |
| Total cholesterol | 2.093 |  |
| HDL cholesterol | -0.746 |  |
| HbA1c | 1.986 |  |
| R-squared | 0.2235 | 0.2047 |
| Residual standard error | 14.59 | 15.1 |

### Treatment for Hypertension and Statins

A large proportion of individuals had missing data for questions relating to whether they received treatment for hypertension or high cholesterol. The majority of non-responses to these questions were coded to suggest that the question was not applicable to the individual. As a consequence it was assumed that individuals with missing treatment data were not taking these medications.

### Gestational Diabetes

Only 30 respondents without current diabetes reported that they had been diagnosed with diabetes during a pregnancy in the past. Most individuals had missing data for this question due to it not being applicable. The missing data was assumed to indicate that individuals had not had gestational diabetes.

### Anxiety/Depression

Most individuals who had missing data for anxiety and depression did so because the question was not applicable. A small sample N=69 refused to answer the question. We assumed that individuals with missing data for anxiety and depression did not have severe anxiety/depression.

### Smoking

Individuals with missing data for smoking status were assumed to be non-smokers, without a history of smoking.

### Rheumatoid Arthritis and Atrial Fibrillation

A very small sample of individuals had missing data for musculoskeletal illness (N=5) and atrial fibrillation (N=1). These individuals were assumed to not suffer from these illnesses.

### Family history of diabetes

No questions in the HSE referred to the individual having a family history of diabetes, so this data had to be imputed. It was important that data was correlated with other risk factors for diabetes, such as HbA1c and ethnicity. We analysed a cross-section of the Whitehall II dataset (9) to generate a logistic regression to describe the probability that an individual has a history of diabetes conditional on their HbA1c and ethnic origin. The model is described in Table 14.

**Table 14: Imputation model for history of diabetes**

|  | **Coefficient** |
| --- | --- |
| Intercept | -3.29077 (0.4430) |
| HbA1c | 0.28960 (0.0840) |
| HDL Cholesterol | 0.81940 (0.13878) |

### Economic Activity

Individuals without information about their employment status were assumed to be retired if aged 65 or over and in employment if under 65.

# GP Attendance in the General Population

GP visit frequency was simulated in the dataset for two reasons; firstly, to estimate the healthcare utilisation for the general population without diabetes and cardiovascular disease and secondly, to predict the likelihood that individuals participate in opportunistic screening for diabetes and vascular risks. Analysis of wave 1 of the Yorkshire Health Study (Table 15: Model 1) investigated whether disease comorbidity, BMI, IMD deprivation score, ethnicity and EQ-5D contributed to the rate of GP attendance (10). The analysis used a negative binomial regression model to estimate self-reported rate of GP attendance per 3 months. The results show that non-white individuals and those from poorer backgrounds visit the GP more frequently. This suggested that GP attendance would be a poor proxy for uptake of screening and prevention services, which are known to be lower in deprived groups. It is possible that higher GP attendance in deprived and ethnic groups reflect poorer health amongst these communities. Model 2 was used in the final model to describe GP attendance conditional on age, sex, BMI, ethnicity, and health outcomes. We did not relate GP attendance to IMD, because we did not have accurate IMD data in the HSE 2011, and EQ-5D was removed to avoid double counting with disease outcomes. The estimated number of GP visits was multiplied by 4 to reflect the annual number of visits per year.

Table 15: GP attendance reported in the Yorkshire Health Study (N= 18,437)

|  | **Model 1** | | **Model 2** | |
| --- | --- | --- | --- | --- |
|  | Mean | Standard error | Mean | Standard error |
| Age | 0.0057 | 0.0005 | 0.0076 | 0.0005 |
| Male | -0.1502 | 0.0155 | -0.1495 | 0.0159 |
| BMI | 0.0020 | 0.0015 | 0.0110 | 0.0015 |
| IMD score 2010 | 0.0043 | 0.0005 |  |  |
| Ethnicity (Non-white) | 0.1814 | 0.0370 | 0.2620 | 0.0375 |
| Heart Disease | 0.1588 | 0.0281 | 0.2533 | 0.0289 |
| Depression | 0.2390 | 0.0240 | 0.6127 | 0.0224 |
| Osteoarthritis | 0.0313 | 0.0240 | 0.2641 | 0.0238 |
| Diabetes | 0.2023 | 0.0270 | 0.2702 | 0.0278 |
| Stroke | 0.0069 | 0.0460 | 0.1659 | 0.0474 |
| Cancer | 0.1908 | 0.0400 | 0.2672 | 0.0414 |
| Intercept | 0.6275 | 0.0590 | -0.5014 | 0.0468 |
| Alpha | 0.3328 | 0.0097 | 0.3423 | 0.0108 |

The coefficients of the Negative Binomial model described in Table 15, were used to estimate the first parameter of the Negative Binomial distribution$\mu_{i}$.

|  | $\mu_{i}=exp(x_{i}\beta)$ |  |
| --- | --- | --- |

The dispersion parameter of the Negative Binomial distribution $v_{i}$ was sampled from a gamma distribution with mean 1 and variance $\alpha$ based on estimates reported in. The dose was estimated from the Poisson function.

|  | $p\left( Y=y \vert y>0,x \right)=\frac{\left( v_{i}\mu_{i} \right)^{y}e^{-\left( v_{i}\mu_{i} \right)}}{y!}$ |  |
| --- | --- | --- |

In the probabilistic sensitivity analysis the parameters of the Yorkshire Health Study negative binomial model are sampled from a multivariate normal distribution, using the mean estimates described in Table 15 and covariance matrix in Table 16.

Table 16: Variance-covariance matrix for GP attendance regression

|  | Age | Male | BMI | Ethnicity (Non-white) | Heart Disease | Depression | Osteoarthritis | Diabetes | Stroke | Cancer | Intercept | Alpha |
| --- | --- | --- | --- | --- | --- | --- | --- | --- | --- | --- | --- | --- |
| Age | 0.0000 |  |  |  |  |  |  |  |  |  |  |  |
| Male | 0.0000 | 0.0003 |  |  |  |  |  |  |  |  |  |  |
| BMI | 0.0000 | 0.0000 | 0.0000 |  |  |  |  |  |  |  |  |  |
| Ethnicity (Non-white) | 0.0000 | 0.0000 | 0.0000 | 0.0014 |  |  |  |  |  |  |  |  |
| Heart Disease | 0.0000 | 0.0000 | 0.0000 | 0.0000 | 0.0008 |  |  |  |  |  |  |  |
| Depression | 0.0000 | 0.0000 | 0.0000 | 0.0000 | 0.0000 | 0.0005 |  |  |  |  |  |  |
| Osteoarthritis | 0.0000 | 0.0000 | 0.0000 | 0.0000 | 0.0000 | 0.0000 | 0.0006 |  |  |  |  |  |
| Diabetes | 0.0000 | 0.0000 | 0.0000 | 0.0000 | -0.0001 | 0.0000 | 0.0000 | 0.0008 |  |  |  |  |
| Stroke | 0.0000 | 0.0000 | 0.0000 | 0.0000 | -0.0002 | -0.0001 | 0.0000 | -0.0001 | 0.0022 |  |  |  |
| Cancer | 0.0000 | 0.0000 | 0.0000 | 0.0000 | 0.0000 | 0.0000 | 0.0000 | 0.0000 | -0.0001 | 0.0017 |  |  |
| Intercept | 0.0000 | 0.0000 | -0.0001 | -0.0002 | 0.0002 | 0.0000 | 0.0002 | 0.0003 | 0.0000 | 0.0001 | 0.0022 |  |
| Alpha | 0.0000 | 0.0000 | 0.0000 | 0.0000 | 0.0000 | 0.0000 | 0.0000 | 0.0000 | 0.0000 | 0.0000 | 0.0000 | 0.0010 |

# Longitudinal Trajectories of Metabolic Risk Factors

A search of epidemiology studies of metabolic risk factor trajectories identified a number of studies estimating changes in HbA1c, SBP, total cholesterol, and HDL cholesterol over time from longitudinal studies. However, no analysis had looked at the correlations and associations between these risk factors. Including the correlation between risk factor trajectories was would affect the long term risk profile for cardiovascular disease and other complications, therefore a statistical analysis of the Whitehall II cohort study (9) was developed to describe correlated longitudinal changes in metabolic risk factors. The analysis was developed in collaboration with epidemiologists at University College London, and in consultation with the stakeholder group.

## Whitehall II Data Analysis

Changes in BMI, latent blood glucose, total cholesterol, HDL cholesterol and systolic blood pressure were estimated from statistical analysis of the Whitehall II cohort. The growth factors for all 5 risk factors were estimated using parallel latent growth modelling. This enabled the growth factors for BMI to be implemented as covariates for the growth processes of glycaemia, systolic blood pressure, and total cholesterol ^[[1]](#footnote-1)^. The structural assumptions of the analysis are described in more detail below.

In the Whitehall II data analysis we assume that individuals have an underlying level of glycaemia, which cannot be observed but can be measured by HbA1c, fasting plasma glucose (FPG) and 2-hour glucose. We describe this underlying propensity for diabetes as latent glycaemia. The statistical model estimated the unobservable latent glycaemia and from this identified associations with test results for HbA1c, FPG, and 2-hour glucose. The longitudinal changes in BMI, glycaemia, systolic blood pressure, total and HDL cholesterol could then be estimated through statistical analysis.

These growth factors are conditional on several individual characteristics including age, sex, ethnicity, smoking, family history of CVD, and family history of type 2 diabetes. Deprivation was excluded from the final analysis because it was not associated with the growth models, and it estimated counter-intuitive coefficients. Last known employment grade was considered to be an alternative specification of socioeconomic status. However, this was excluded from the final analysis because it was not a statistically significant predictor of glycaemia. We related the effect of changes in BMI to changes in glycaemia, systolic blood pressure and total cholesterol. If an intervention is known to be effective in reducing BMI and the other metabolic risk factors, the Whitehall II model is adjusted to temporarily remove the indirect effect of the intervention through BMI. This ensures that the effectiveness of the intervention is not over-estimated. Unobservable heterogeneity between individual growth factors not explained by patient characteristics was incorporated into the growth models as random error terms. Correlation between the random error terms for glycaemia, total cholesterol, HDL cholesterol and systolic blood pressure was estimated from the Whitehall II cohort. This means that in the simulation, an individual with a higher growth rate for glycaemia is more likely to have a higher growth rate of total cholesterol and systolic blood pressure.

An advantage of our parallel growth analysis is that we were able to estimate the effect of growth in BMI on the other metabolic risk factors. We were also able to estimate correlation between changes in glycaemia, systolic blood pressure, total cholesterol and HDL cholesterol. As a consequence, the growth factor random error terms were not assumed to be independent and were sampled from a multivariate normal distribution$\boldsymbol{\upsilon}\sim N(0,\Omega)$. Estimates for the covariance matrix are derived from the covariance estimates reported in the statistical analysis.

The baseline observations for BMI, HbA1c, systolic blood pressure, cholesterol and HDL cholesterol were extracted from the Health Survey for England 2011 in order to simulate a representative sample. The predicted intercept for these metabolic risk factors was estimated using the Whitehall II analysis to give population estimates of the individuals’ starting values, conditional on their characteristics. The difference between the simulated and observed baseline risk factors was taken to estimate the individuals’ random deviation from the population expectation. The individual random error in the slope trajectory was sampled from a conditional multivariate normal distribution to allow correlation between the intercept and slope random errors.

Figure 3: Path analysis of final statistical analysis of the Whitehall II cohort

## BMI Trajectory

The Whitehall II analysis estimates longitudinal trajectories for BMI. The path diagram for BMI is illustrated in Figure 3.

Figure 4: The path diagram for BMI growth

We simulated annual changes in BMI for all individuals within the simulation. At baseline, BMI estimates from the HSE determine an individual’s BMI. BMI at any time period in the model is estimated using the following quadratic equation.

$${BMI}_{t}=\beta_{10}+\beta_{11}t+\beta_{12}t^{2}+\varepsilon_{1}$$

$$\beta_{10}=\alpha_{10}+\boldsymbol{\gamma}_{\boldsymbol{10}}\boldsymbol{X}+\upsilon_{10}$$

$$\beta_{11}=\alpha_{11}+\boldsymbol{\gamma}_{\boldsymbol{11}}\boldsymbol{X}+\upsilon_{11}$$

$$\beta_{12}=\alpha_{12}+\boldsymbol{\gamma}_{\boldsymbol{12}}\boldsymbol{X}$$

The intercept of the BMI calculation is described by $\beta_{10}$, the linear slope $\beta_{11}$ , quadratic term $\beta_{12}$ and a measurement error term $\varepsilon_{1}$. The intercept $\beta_{10}$ is conditional on the population mean intercept $\alpha_{10}$, coefficients, $\boldsymbol{\gamma}_{\boldsymbol{10}}$for patient characteristics $\boldsymbol{X}$**,** and an individual level random error term $\upsilon_{10}$. Annual change in BMI is determined by $\beta_{11}$ and $\beta_{12}$, which are also conditional on population intercepts and covariate adjustments. The linear slope includes an individual patient random error term $\upsilon_{11}$, whereas the quadratic slope term does not contain an error term. The conceptual model for BMI assumes that age at baseline, sex and family history of cardiovascular disease predict the intercept, slope and quadratic term in the BMI model. Figure 4 illustrates simulated changes in BMI over time for a man and women aged 50 at baseline.

Figure 5: Simulated data using the Whitehall II Statistical analysis to illustrate BMI over time

The growth factors are estimated in the model to be conditional on baseline BMI, age at baseline, sex, Townsend deprivation index, family history of cardiovascular disease and an error parameter to reflect unobservable variability in growth trajectories between individuals. This allows us to relate the rate of BMI growth to individual characteristics. For example, in our statistical analysis we found that the rate of BMI growth was lower in men and older adults.

## Glycaemic Trajectory in Non-Diabetics/undiagnosed Diabetes

We simulated annual changes in HbA1c, FPG, and 2-hr glucose within the simulation. We conceptualised a latent glycaemic variable to describe an individual’s underlying level of hyper-glycaemia. Latent glycaemia can be tested using 2-hr glucose, FPG or HbA1c tests, but each test is an imperfect measure of the individual’s true glycaemic status. The path diagram to describe changes in glycaemia, 2-hr glucose, FPG and HbA1c from the Whitehall II cohort is illustrated in Figure 5.

Figure 6: The path diagram for glycaemic growth

We assume that changes in latent glycaemia have a quadratic relationship with time. Latent glycaemia (glyc) at any time period in the model is estimated using the following quadratic equation.

$${glyc}_{t}=\beta_{20}+\beta_{21}t+\beta_{22}t^{2}+\varepsilon_{2}$$

$$\beta_{20}=\alpha_{20}+\boldsymbol{\gamma}_{\boldsymbol{20}}\boldsymbol{X}+{\tau_{20}\beta_{10}+\upsilon}_{20}$$

$$\beta_{21}=\alpha_{21}+\boldsymbol{\gamma}_{\boldsymbol{21}}\boldsymbol{X+}\tau_{21}\beta_{10}+\tau_{22}\beta_{11}+\upsilon_{21}$$

$$\beta_{22}=\alpha_{22}+\boldsymbol{\gamma}_{\boldsymbol{22}}\boldsymbol{X}+\upsilon_{22}$$

The intercept of the latent glycaemia is described by $\beta_{20}$, the linear slope $\beta_{21}$ , quadratic term $\beta_{22}$ and a measurement error term $\varepsilon_{2}$. The intercept $\beta_{20}$ is conditional on the population mean intercept $\alpha_{20}$, coefficients $\boldsymbol{\gamma}_{\boldsymbol{20}}$for patient characteristics $\boldsymbol{X}$**,** the growth intercept for BMI $\beta_{10}$, and an individual level random error term $\upsilon_{20}$. Annual change in latent glycaemia is determined by $\beta_{21}$ and $\beta_{22}$, which are also conditional on population intercepts, covariate adjustments and individual level random error terms. The growth factors $\beta_{20}$ and $\beta_{21}$ are also conditional on the growth factors for BMI.

The glycaemic test results (FPG, 2-hr glucose, HbA1c) were assumed to be conditional on latent glycaemia, *glyc*. The model estimates test results for each period of observation (*t=*1,2,3,4…*t*). The factor glycaemia is measured by three non-overlapping observations of 2-hr glucose, FPG and HbA1c. The scale of the factor is fixed by setting one factor loading (2-hr glucose) to 1.

$$\left[ \begin{matrix} {FPG}_{t} \\ {2HR}_{t} \\ {A1C}_{t} \end{matrix} \right]=\left[ \begin{matrix} \mu_{0} \\ \mu_{1} \\ \mu_{2} \end{matrix} \right]+\left[ \begin{matrix} \theta_{01} \\ \theta_{11} \\ \theta_{21} \end{matrix} \right]\left[ {glyc}_{t} \right]+\left[ \begin{matrix} \theta_{02} \\ \theta_{12} \\ \theta_{22} \end{matrix} \right]\left[ {AGE}_{t} \right]+\left[ \begin{matrix} \theta_{03} \\ \theta_{13} \\ \theta_{23} \end{matrix} \right]\left[ SEX \right]+\left[ \begin{matrix} \theta_{04} \\ \theta_{14} \\ \theta_{24} \end{matrix} \right]\left[ ETHN \right]+\left[ \begin{matrix} \theta_{05} \\ \theta_{15} \\ \theta_{25} \end{matrix} \right]\left[ FXT2 \right]+\left[ \begin{matrix} \varepsilon_{20} \\ \varepsilon_{21} \\ \varepsilon_{22} \end{matrix} \right]$$

We assumed that the models to predict blood glucose were constant with time. The parameters that determine an individual’s latent glycaemic growth trajectory are a function of sex, ethnicity, age at baseline, non-white ethnicity, smoking and family history of type 2 diabetes. Furthermore, the statistical analysis found that an increase in BMI will accelerate the rate of growth in latent glycaemia. The effect of BMI on simulated HbA1c for an example individual is illustrated in Figure 6. The trajectory for an individual with increasing BMI (0.21kg/m^2^ per year) is steeper than that with zero change in BMI.

Figure 7: Simulated data using the Whitehall II Statistical analysis to illustrate the effect of BMI on HbA1c trajectories

At baseline, HbA1c estimates from the HSE determine HbA1c in year 1. We used this measure of HbA1c to estimate corresponding FPG and 2-hr glucose test results. We chose to simulate all three tests to allow comparisons of screening methods within the model. We have designed the model so that the three tests are correlated, but imperfectly so. This means that it is possible that an individual will be classified as diabetic using an HbA1c test, but would be below the threshold for diabetes using the FPG test.

## HbA1c trajectory in type 2 diagnosed diabetics

Following a diagnosis of diabetes in the simulation all individuals experience an initial fall in HbA1c due to changes in diet and lifestyle as observed in the UKPDS trial (11). We have estimated the expected change in HbA1c conditional on HbA1c at diagnosis by fitting a simple linear regression to three aggregate outcomes reported in the study. These showed that the change in HbA1c increases for higher HbA1c scores at diagnosis. The regression parameters to estimate change in HbA1c are reported in Table 17.

Table 17: Estimated change in HbA1c following diabetes diagnosis

|  | **Mean** | **Standard error** |
| --- | --- | --- |
| Change in HbA1c Intercept | -2.9465 | 0.0444513 |
| HbA1c at baseline | 0.5184 | 0.4521958 |

After this initial reduction in HbA1c the longitudinal trajectory of HbA1c is estimated using the UKPDS outcomes model (12) rather than the Whitehall II statistical analysis. The UKPDs dataset is made up of a newly diagnosed diabetic population. As part of the UKPDS Outcomes model, longitudinal trial data were analysed using a random effects model, which means that unobservable differences between individuals are accounted for in the analysis. The coefficients of the model are reported in Table 18.

**Table 18: Coefficient estimates for HbA1c estimated from UKPDS data**

|  | **Mean Coefficient** | **Coefficient standard error** |
| --- | --- | --- |
| Intercept | -0.024 | 0.017 |
| Log transformation of year since diagnosis | 0.144 | 0.009 |
| Binary variable for year after diagnosis | -0.333 | 0.05 |
| HbA1c score in last period | 0.759 | 0.004 |
| HbA1c score at diagnosis | 0.085 | 0.004 |

The model can be used to predict HbA1c over time from the point of diagnosis. The model suggests that HbA1c increases with time. A graph illustrating change in HbA1c over time from two different HbA1c levels at diagnosis is illustrated in Figure 7.

Figure 8: Trajectory of HbA1c estimated from UKPDS longitudinal model

It was important to maintain heterogeneity in the individual glycaemic trajectories before and after diagnosis. Therefore, the random error terms used to determine individual trajectories in glycaemia before diagnosis were used to induce random noise in the trajectory after diagnosis. We sampled the expected random error term for each individual after diagnosis conditional on pre-diagnosis slope, assuming a 0.8 correlation between these values.

## Total Cholesterol and HDL cholesterol Trajectories in Individuals not receiving Statins

In the simulation, individuals had annual changes in total and HDL cholesterol according to the estimates from the statistical analysis of the Whitehall II cohort. The path diagram for total and HDL cholesterol is illustrated in Figure 8.

Figure 9: The path diagram for total and HDL cholesterol growth

Total cholesterol (TC) at any time period in the model is estimated using the following linear equation.

$${TC}_{t}=\beta_{40}+\beta_{41}t+\varepsilon_{4}$$

$$\beta_{40}=\alpha_{40}+\boldsymbol{\gamma}_{\boldsymbol{40}}\boldsymbol{X}+{\tau_{40}\beta_{10}+\upsilon}_{40}$$

$$\beta_{41}=\alpha_{41}+\boldsymbol{\gamma}_{\boldsymbol{41}}\boldsymbol{X+}\tau_{41}\beta_{10}\boldsymbol{+}\tau_{41}\beta_{11}+\upsilon_{41}$$

The intercept of the total cholesterol growth model is described by $\beta_{40}$, the linear slope $\beta_{41}$ and a measurement error term $\varepsilon_{4}$. The intercept $\beta_{40}$ is conditional on the population mean intercept $\alpha_{40}$, coefficients $\boldsymbol{\gamma}_{\boldsymbol{40}}$for patient characteristics $\boldsymbol{X}$**,** a factor $\tau_{40}$, describing the association with the growth intercept for BMI $\beta_{10}$, and an individual level random error term $\upsilon_{40}$. Annual change in TC is determined by $\beta_{41}$, which is also conditional on population intercepts, covariate adjustments and an individual level random error term. Growth in total cholesterol is conditional on baseline BMI and the growth rate of BMI.

HDL cholesterol (HDL) at any time period in the model is estimated using the following linear equation.

$${HDL}_{t}=\beta_{50}+\beta_{51}t+\varepsilon_{5}$$

$$\beta_{50}=\alpha_{50}+\boldsymbol{\gamma}_{\boldsymbol{50}}\boldsymbol{X}+{\tau_{51}\beta_{10}+\upsilon}_{50}$$

$$\beta_{51}=\alpha_{51}+\boldsymbol{\gamma}_{\boldsymbol{51}}\boldsymbol{X+}\tau_{51}\beta_{10}+\upsilon_{51}$$

The intercept of the HDL cholesterol growth model is described by $\beta_{50}$, the linear slope $\beta_{51}$ and a measurement error term $\varepsilon_{5}$. The intercept $\beta_{50}$ is conditional on the population mean intercept $\alpha_{50}$, coefficients $\boldsymbol{\gamma}_{\boldsymbol{50}}$for patient characteristics $\boldsymbol{X}$**,** a factor $\tau_{50}$, describing the association with the growth intercept for BMI $\beta_{10}$, and an individual level random error term $\upsilon_{50}$. Annual change in HDL cholesterol is determined by $\beta_{51}$, which is also conditional on population intercepts, covariate adjustments and an individual level random error term. Growth in HDL is conditional on baseline BMI only.

At baseline, an individual’s total and HDL cholesterol is determined from the HSE 2011 data. The slope of total and HDL cholesterol are assumed to be linear with time. These growth factors are estimated in the model to be conditional on cholesterol at baseline, age at baseline, sex, and an error parameter to reflect unobservable variability in growth trajectories between individuals. As with latent glycaemia, changes in total cholesterol are also influenced by the trajectory of BMI. Figure 9 illustrates the trajectories for total and HDL cholesterol according to changes in BMI. We did not identify if changes in BMI impact upon changes in HDL cholesterol.

Figure 10: Simulated data using the Whitehall II Statistical analysis to illustrate the effect of BMI on cholesterol

## Total Cholesterol and HDL cholesterol Trajectories in Individuals receiving Statins

During the simulation process, individuals are prescribed statins to reduce their risk of cardiovascular disease. It is assumed within the model that the statins are effective in reducing an individual’s total cholesterol, and an average effect is applied to all patients receiving statins. A recent HTA reviewed the literature on the effectiveness and cost-effectiveness of statins in individuals with acute coronary syndrome (13). This report estimated the change in LDL cholesterol for four statin treatments and doses compared with placebo from a Bayesian meta-analysis. The analysis estimated a reduction in LDL cholesterol of -1.45 for simvastatin. This estimate was used to describe the effect of statins in reducing total cholesterol. It was assumed that the effect was instantaneous upon receiving statins and maintained as long as the individual receives statins. It was also assumed that individuals receiving statins no longer experienced annual changes in cholesterol. HDL cholesterol was assumed constant over time if patients receive statins.

Non-adherence to statin treatment is a common problem. Two recent HTAs reviewed the literature on continuation and compliance with statin treatment. They both concluded that there was a lack of adequate reporting, but that the proportion of patients fully compliant with treatment appears to decrease with time, particularly in the first 12 months after initiating treatment, and can fall below 60% after five years (13;14). Although a certain amount of non-compliance is included within trial data, clinical trials are not considered to be representative of continuation and compliance in general practice. A yearly reduction in statin compliance used in the HTA analysis is reported in Table 19. It is based on the published estimate of compliance for the first five years of statin treatment for primary prevention in general clinical practice (14). Compliance declines to a minimum of 65% after five years of treatment. It is assumed that there is no further drop after five years.

Table 19: Proportion of patients assumed to be compliant with statin treatment, derived from Table 62 in (12)

| Year after statin initiation | 1 | 2 | 3 | 4 | 5 |
| --- | --- | --- | --- | --- | --- |
| Proportion compliant | 0.8 | 0.7 | 0.68 | 0.65 | 0.65 |

In the simulation, we assume in the base case that only 65% of individuals initiate statins when they are deemed eligible. However those that initiate statins remain on statins for their lifetime. Those who refuse statins may be prescribed them again at a later date.

## Systolic Blood Pressure Trajectories in Individuals not receiving Anti-hypertensive treatment

In the simulation, individuals’ systolic blood pressure changes every year according to the estimates from the statistical analysis of the Whitehall II cohort. The Path diagram for systolic blood pressure is illustrated in Figure 10.

Figure 11: Systolic blood pressure path diagram for Whitehall II Statistical analysis

Systolic blood pressure at any time period in the model is estimated using the following linear equation.

$${SBP}_{t}=\beta_{30}+\beta_{31}t+\varepsilon_{3}$$

$$\beta_{30}=\alpha_{30}+\boldsymbol{\gamma}_{\boldsymbol{30}}\boldsymbol{X}+{\tau_{30}\beta_{10}+\upsilon}_{30}$$

$$\beta_{31}=\alpha_{31}+\boldsymbol{\gamma}_{\boldsymbol{31}}\boldsymbol{X+}\tau_{31}\beta_{10}\boldsymbol{+}\tau_{32}\beta_{11}+\upsilon_{31}$$

The intercept of the systolic blood pressure growth model is described by $\beta_{30}$, the linear slope $\beta_{31}$ and a measurement error term $\varepsilon_{3}$. The intercept $\beta_{30}$ is conditional on the population mean intercept $\alpha_{30}$, coefficients $\boldsymbol{\gamma}_{\boldsymbol{30}}$for patient characteristics $\boldsymbol{X}$**,** the growth intercept for BMI, and an individual level random error term $\upsilon_{30}$. Annual change in SBP is determined by $\beta_{31}$, which is also conditional on population intercepts, covariate adjustments and an individual level random error term. Growth in SBP is also conditional on baseline BMI and the growth rate of BMI.

The annual change in systolic blood pressure is assumed to be linear with time. At baseline an individual’s systolic blood pressure is determined from the HSE 2011 data. The growth factors are estimated in the model to be conditional on systolic blood pressure at baseline, age at baseline, sex, ethnicity, family history of cardiovascular disease, smoking and an error parameter to reflect unobservable variability in growth trajectories between individuals. Changes in systolic blood pressure are also influenced by the trajectory of BMI as illustrated in Figure 11.

Figure 12: Simulated data using the Whitehall II Statistical analysis to illustrate the effect of BMI on blood pressure

## Systolic Blood Pressure Trajectories in Individuals receiving Anti-hypertensive treatment

During the simulation process, if individuals are identified as having systolic blood pressure higher than 160mm Hg, or higher than 140mm Hg with comorbid diabetes, cardiovascular disease, or 10 year risk of cardiovascular disease greater than 20%, they will be prescribed anti-hypertensive treatment (15). The change in systolic blood pressure following initiation of calcium channel blockers was estimated in a meta-analysis of anti-hypertensive treatments (16). This study identified an average change in systolic blood pressure of -8.4 for monotherapy with calcium channel blockers. It is assumed that this reduction in systolic blood pressure is maintained for as long as the individual receives anti-hypertensive treatment. For simplicity we do not assume that the individual switches between anti-hypertensive treatments over time. Once an individual is receiving anti-hypertensive treatment it is assumed that their systolic blood pressure is stable and does not change over time.

## Metabolic Risk factor screening

We assume that individuals eligible for anti-hypertensive treatment or statins will be identified through opportunistic screening if they meet certain criteria and attend the GP for at least one visit in the simulation period.

1. Individuals with a history of cardiovascular disease;
2. Individuals with a major microvascular event (foot ulcer, blindness, renal failure or amputation);
3. Individuals with diagnosed diabetes;
4. Individuals identified with Impaired Glucose Regulation;
5. Individuals with systolic blood pressure greater than 160mmHg.

Individuals may also be detected for diabetes through opportunistic screening if the following criteria are met.

1. Individuals with a history of cardiovascular disease;
2. Individuals with a major microvascular event (foot ulcer, blindness, renal failure or amputation);
3. Individuals identified with impaired glucose regulation;
4. At baseline individuals are assigned an HbA1c threshold above which diabetes is detected opportunistically, individuals with an HbA1c above their individual threshold will attend the GP to be diagnosed with diabetes. The threshold is sampled from the distribution of HbA1c tests in a cohort of recently diagnosed patients in clinical practice (17).

The base case has been designed to represent a health system with moderate levels of screening for hypertension, diabetes, and dyslipidaemia. Alternative assumptions for more or less intensive opportunistic screening can be assumed.

## Diagnosis and Treatment Initiation

It is assumed that there are three, non-mutually exclusive outcomes from the vascular checks or opportunistic screening. Firstly, that the patient receives statins to reduce cardiovascular risk. Secondly, that the patient has high blood pressure and should be treated with anti-hypertensive medication. Thirdly, the model evaluates whether the blood glucose test indicates a diagnosis with type 2 diabetes. The following threshold estimates were used to determine these outcomes.

1. Statins are initiated if the individual has greater than or equal to 20% 10 year CVD risk estimated from the QRISK2 2012 algorithm (18).
2. Anti-hypertensive treatment is initiated if systolic blood pressure is greater than 160. If the individual has a history of CVD, diabetes or a CVD risk >20%, the threshold for systolic blood pressure is 140 (15).
3. Type 2 diabetes is diagnosed if the individual has two HbA1c tests greater than 48 mmol/mol (6.5%). In the base case it is assumed that FPG and 2-hr glucose are not used for diabetes diagnosis. However, future adaptations of the model could use these tests for diagnosis.

# Comorbid Outcomes and Mortality

In every model cycle individuals within the model are evaluated to determine whether they have a clinical event, including mortality, within the cycle period. In each case the simulation estimates the probability that an individual has the event and uses a random number draw to determine whether the event occurred.

## Cardiovascular Disease

### First Cardiovascular event

Several statistical models for cardiovascular events were identified in a review of economic evaluations for diabetes prevention (1). The UKPDS outcomes model (20), Framingham risk equation (21) and QRISK2 (22) have all been used in previous models to estimate cardiovascular events. The Framingham risk equation was not adopted because, unlike the QRISK2 model, it is not estimated from a UK population. The UKPDS outcomes model would be ideally suited to estimate the risk of cardiovascular disease in a population diagnosed with type 2 diabetes. Whilst this is an important outcome of the cost-effectiveness model, there was concern that it would not be representative of individuals with normal glucose tolerance or impaired glucose regulation. Recent analyses show that the UKPDS over-predicts cardiovascular outcomes in newly diagnosed diabetes patients (23). It was important that reductions in cardiovascular disease risk in these populations were represented to capture the population-wide benefits of public health interventions. The QRISK2 model was selected for use in the cost-effectiveness model because it is a validated model of cardiovascular risk in an up to date UK population that could be used to generate probabilities for diabetic and non-diabetic populations. We considered using the UKPDS outcomes model specifically to estimate cardiovascular risk in patients with type 2 diabetes. However, it would not be possible to control for shifts in absolute risk generated by the different risk scores due to different baselines and covariates. This would lead to some individuals experiencing counterintuitive and favourable shifts in risk after onset of type 2 diabetes. Therefore, we decided to use diabetes as a covariate adjustment to the QRISK2 model to ensure that the change in individual status was consistent across individuals.

The probability of the first cardiovascular event is estimated from the QRISK2 predicted model of cardiovascular disease (20). The QRISK2 is a validated risk prediction algorithm to identify individuals at high risk of cardiovascular disease. The algorithm was developed from UK data and incorporates social deprivation and ethnicity. We accessed the 2012 version from the online QRISK (24). The QRISK2 equation estimates the probability of a cardiovascular event in the next year conditional on ethnicity, smoking status, age, BMI, ratio of total/HDL cholesterol, Townsend score, atrial fibrillation, rheumatoid arthritis, renal disease, hypertension, diabetes, and family history of cardiovascular disease. Data on all these variables was available from the HSE 2011. Table 20 reports the coefficient estimates for the QRISK2 algorithm. The standard errors were not reported within the open source code. Where possible, standard errors were imputed from a previous publication of the risk equation (25). Coefficients that were not reported in this publication were assumed to have standard errors of 20%.

Table 20: Coefficients from the 2012 QRISK2 risk equation and estimate standard errors

|  | **Estimated coefficients adjusting for individual characteristics** | | | | | | | | |
| --- | --- | --- | --- | --- | --- | --- | --- | --- | --- |
|  | Women | | Men | |  | Women | | Men | |
| Covariates | Mean | Standard error | Mean | Mean | Interaction terms | Mean | Standard error | Mean | Standard error |
| White | 0.0000 | 0.0000 | 0.0000 | 0.0000 | Age1*former smoker | 0.1774 | 0.035 | -3.881 | 0.776 |
| Indian | 0.2163 | 0.0537 | 0.3163 | 0.0425 | Age1*light smoker | -0.3277 | 0.066 | -16.703 | 3.341 |
| Pakistani | 0.6905 | 0.0698 | 0.6092 | 0.0547 | Age1*moderate smoker | -1.1533 | 0.231 | -15.374 | 3.075 |
| Bangladeshi | 0.3423 | 0.1073 | 0.5958 | 0.0727 | Age1*Heavy smoker | -1.5397 | 0.308 | -17.645 | 3.529 |
| Other Asian | 0.0731 | 0.1071 | 0.1142 | 0.0845 | Age1*AF | -4.6084 | 0.922 | -7.028 | 1.406 |
| Caribbean | -0.0989 | 0.0619 | -0.3489 | 0.0641 | Age1*renal disease | -2.6401 | 0.528 | -17.015 | 3.403 |
| Black African | -0.2352 | 0.1275 | -0.3604 | 0.1094 | Age1*hypertension | -2.2480 | 0.450 | 33.963 | 6.793 |
| Chinese | -0.2956 | 0.1721 | -0.2666 | 0.1538 | Age1*Diabetes | -1.8452 | 0.369 | 12.789 | 2.558 |
| Other | -0.1010 | 0.0793 | -0.1208 | 0.0734 | Age1*BMI | -3.0851 | 0.617 | 3.268 | 0.654 |
| Non-smoker | 0.0000 | 0.0000 | 0.0000 | 0.0000 | Age1*family history CVD | -0.2481 | 0.050 | -17.922 | 3.584 |
| Former smoker | 0.2033 | 0.0152 | 0.2684 | 0.0108 | Age1*SBP | -0.0132 | 0.003 | -0.151 | 0.030 |
| Light smoker | 0.4820 | 0.0220 | 0.5005 | 0.0166 | Age1*Townsend | -0.0369 | 0.007 | -2.550 | 0.510 |
| Moderate smoker | 0.6126 | 0.0178 | 0.6375 | 0.0148 | Age2*former smoker | -0.0051 | 0.001 | 7.971 | 1.594 |
| Heavy smoker | 0.7481 | 0.0194 | 0.7424 | 0.0143 | Age2*light smoker | -0.0005 | 0.000 | 23.686 | 4.737 |
| Age 1* | 5.0327 |  | 47.3164 |  | Age2*moderate smoker | 0.0105 | 0.002 | 23.137 | 4.627 |
| Age 2* | -0.0108 |  | -101.2362 |  | Age2*Heavy smoker | 0.0155 | 0.003 | 26.867 | 5.373 |
| BMI* | -0.4724 | 0.0423 | 0.5425 | 0.0299 | Age2*AF | 0.0507 | 0.010 | 14.452 | 2.890 |
| Ratio Total / HDL chol | 0.1326 | 0.0044 | 0.1443 | 0.0022 | Age2*renal disease | 0.0343 | 0.007 | 28.270 | 5.654 |
| SBP | 0.0106 | 0.0045 | 0.0081 | 0.0046 | Age2*hypertension | 0.0258 | 0.005 | -18.817 | 3.763 |
| Townsend | 0.0597 | 0.0068 | 0.0365 | 0.0048 | Age2*Diabetes | 0.0180 | 0.004 | 0.963 | 0.193 |
| AF | 1.3261 | 0.0310 | 0.7547 | 0.1018 | Age2*BMI | 0.0345 | 0.007 | 10.551 | 2.110 |
| Rheumatoid arthritis | 0.3626 | 0.0319 | 0.3089 | 0.0445 | Age2*family history CVD | -0.0062 | 0.001 | 26.605 | 5.321 |
| Renal disease | 0.7636 | 0.0639 | 0.7441 | 0.0702 | Age2*SBP | 0.0000 | 0.000 | 0.291 | 0.058 |
| Hypertension | 0.5421 | 0.0115 | 0.4978 | 0.0112 | Age2*Townsend | -0.0011 | 0.000 | 3.007 | 0.601 |
| Diabetes | 0.8940 | 0.0199 | 0.7776 | 0.0175 |  |  |  |  |  |
| Family history of CVD | 0.5997 | 0.0122 | 0.6965 | 0.0111 |  |  |  |  |  |
| AF Atrial Fibrillation CVD Cardiovascular disease SBP systolic blood pressure * covariates transformed with fractional polynomials | | | | | | | | | |

The QRISK2 risk equation can be used to calculate the probability of a cardiovascular event including coronary heart disease (angina or myocardial infarction), stroke, transient ischaemic attacks and fatality due to cardiovascular disease. The equation estimates the probability of a cardiovascular event in the next period conditional on the coefficients listed in Table 20. The equation for the probability of an event in the next period is calculated as

$$p\left( Y=1 \right)=1-{S(1)}^{\theta}$$

$$\theta=\sum\beta X$$

The probability of an event is calculated from the survival function at 1 year raised to the power of $\theta$, where $\theta$ is the sum product of the coefficients reported in Table 20 multiplied by the individual’s characteristics. Underlying survival curves for men and women were extracted from the QRISK2 open source file. Mean estimates for the continuous variables were also reported in the open source files.

We modified the QRISK assumptions regarding the relationship between IGR, diabetes and cardiovascular disease. Firstly, we assumed that individuals with HbA1c>48 mmol/mol (6.5%) have an increased risk of cardiovascular disease even if they have not received a formal diagnosis. Secondly, risk of cardiovascular disease was assumed to increase with HbA1c for test results greater than 48 mmol/mol (6.5%) to reflect observations from the UKPDS that HbA1c increases the risk of MI and Stroke (20). Thirdly, prior to type 2 diabetes (HbA1c>48 mmol/mol (6.5%)) HbA1c is linearly associated with cardiovascular disease. A study from the EPIC Cohort ((26)) has found that a unit increase in HbA1c increases the risk of coronary heart disease by a hazard ratio of 1.25, after adjustment for other risk factors. We apply this risk ratio to linearly increase risk above the mean HBA1c observed in the HSE 2011 cohort. A linear risk reduction was applied at HbA1c levels below the HSE mean.

The QRISK2 algorithm identifies which individuals experience a cardiovascular event but does not specify the nature of the event. The nature of the cardiovascular event was determined independently. A targeted search of recent Health Technology appraisals of cardiovascular disease was performed to identify a model for the progression of cardiovascular disease following a first event. All QRISK events are assigned to a specific diagnosis according to age and sex specific distributions of cardiovascular events used in a previous Health Technology Assessment (HTA) (14). Table 21 reports the probability of cardiovascular outcomes by age and gender. Stakeholders suggested that there may be different relationships between the risk factors and the different types of CVD (eg. hypertension is more of a risk factor for stroke). However, we decided not to incorporate these factors in evaluating the risk of cardiovascular event types due to a lack of evidence.

Table 21: The probability distribution of cardiovascular events by age and gender

|  | **Age** | **Stable angina** | **Unstable angina** | **MI rate** | **Fatal CHD** | **TIA** | **Stroke** | **Fatal CVD** |
| --- | --- | --- | --- | --- | --- | --- | --- | --- |
| Men | 45-54 | 0.307 | 0.107 | 0.295 | 0.071 | 0.060 | 0.129 | 0.030 |
|  | 55-64 | 0.328 | 0.071 | 0.172 | 0.086 | 0.089 | 0.206 | 0.048 |
|  | 65-74 | 0.214 | 0.083 | 0.173 | 0.097 | 0.100 | 0.270 | 0.063 |
|  | 75-84 | 0.191 | 0.081 | 0.161 | 0.063 | 0.080 | 0.343 | 0.080 |
|  | 85+ | 0.214 | 0.096 | 0.186 | 0.055 | 0.016 | 0.351 | 0.082 |
| Women | 45-54 | 0.325 | 0.117 | 0.080 | 0.037 | 0.160 | 0.229 | 0.054 |
|  | 55-64 | 0.346 | 0.073 | 0.092 | 0.039 | 0.095 | 0.288 | 0.067 |
|  | 65-74 | 0.202 | 0.052 | 0.121 | 0.081 | 0.073 | 0.382 | 0.090 |
|  | 75-84 | 0.149 | 0.034 | 0.102 | 0.043 | 0.098 | 0.464 | 0.109 |
|  | 85+ | 0.136 | 0.029 | 0.100 | 0.030 | 0.087 | 0.501 | 0.117 |

### Subsequent Cardiovascular events

After an individual has experienced a cardiovascular event, it is not possible to predict the transition to subsequent cardiovascular events using QRISK2. Instead, as with assigning first CVD events, the probability of subsequent events was estimated from the HTA evaluating statins (14). This study reported the probability of future events, conditional on the nature of the previous event. Table 22 to Table 26 report the probabilities within a year of transitioning from stable angina, unstable angina, myocardial infarction (MI), transient ischemic attack (TIA) or stroke for individuals in different age groups. The tables suggests that, for example 99.46% of individuals with stable angina will remain in the stable angina state, but 0.13%, 0.32% and 0.01% will progress to unstable angina, MI or death from coronary heart disease (CHD) respectively.

Table 22: Probability of cardiovascular event conditional on age and status of previous event (age 45-54)

| **Age 45-54** | | To | | | | | | | | | |
| --- | --- | --- | --- | --- | --- | --- | --- | --- | --- | --- | --- |
|  |  | Stable angina | Unstable angina 1 | Unstable angina 2 | MI 1 | MI 2 | TIA | Stroke 1 | Stroke 2 | CHD death | CVD death |
|  |  |  |  |  |  |  |  |  |  |  |  |
| From | Stable angina | 0.9946 | 0.0013 | 0 | 0.0032 | 0 | 0 | 0 | 0 | 0.0009 | 0 |
|  | Unstable angina (1^st^ yr) | 0 | 0 | 0.9127 | 0.0495 | 0 | 0 | 0 | 0 | 0.0362 | 0.0016 |
|  | Unstable angina (subsequent) | 0 | 0 | 0.9729 | 0.0186 | 0 | 0 | 0 | 0 | 0.0081 | 0.0004 |
|  | MI (1^st^ yr) | 0 | 0 | 0 | 0.128 | 0.8531 | 0 | 0.0015 | 0 | 0.0167 | 0.0007 |
|  | MI (subsequent) | 0 | 0 | 0 | 0.0162 | 0.978 | 0 | 0.0004 | 0 | 0.0052 | 0.0002 |
|  | TIA | 0 | 0 | 0 | 0.0016 | 0 | 0.9912 | 0.0035 | 0 | 0.0024 | 0.0013 |
|  | Stroke (1^st^ yr) | 0 | 0 | 0 | 0.0016 | 0 | 0 | 0.0431 | 0.9461 | 0.0046 | 0.0046 |
|  | Stroke (subsequent) | 0 | 0 | 0 | 0.0016 | 0 | 0 | 0.0144 | 0.9798 | 0.0021 | 0.0021 |
| MI Myocardial Infarction; TIA Transient Ischemic Attack; CHD Coronary Heart Disease; CVD Cerebrovascular disease | | | | | | | | | | | |

Table 23: Probability of cardiovascular event conditional on age and status of previous event (age 55-64)

| **Age 55-64** | | To | | | | | | | | | |
| --- | --- | --- | --- | --- | --- | --- | --- | --- | --- | --- | --- |
|  |  | Stable angina | Unstable angina 1 | Unstable angina 2 | MI 1 | MI 2 | TIA | Stroke 1 | Stroke 2 | CHD death | CVD death |
|  |  |  |  |  |  |  |  |  |  |  |  |
| From | Stable angina | 0.9880 | 0.0033 | 0 | 0.0057 | 0 | 0 | 0 | 0 | 0.0030 | 0 |
|  | Unstable angina (1^st^ yr) | 0 | 0 | 0.8670 | 0.0494 | 0 | 0 | 0 | 0 | 0.0800 | 0.0036 |
|  | Unstable angina (subsequent) | 0 | 0 | 0.9415 | 0.0471 | 0 | 0 | 0 | 0 | 0.0109 | 0.0005 |
|  | MI (1^st^ yr) | 0 | 0 | 0 | 0.1087 | 0.8409 | 0 | 0.0047 | 0 | 0.0439 | 0.0019 |
|  | MI (subsequent) | 0 | 0 | 0 | 0.0183 | 0.9678 | 0 | 0.0015 | 0 | 0.0119 | 0.0005 |
|  | TIA | 0 | 0 | 0 | 0.0029 | 0 | 0.9666 | 0.0159 | 0 | 0.0079 | 0.0068 |
|  | Stroke (1^st^ yr) | 0 | 0 | 0 | 0.0029 | 0 | 0 | 0.0471 | 0.9159 | 0.0171 | 0.0171 |
|  | Stroke (subsequent) | 0 | 0 | 0 | 0.0029 | 0 | 0 | 0.0205 | 0.9622 | 0.0072 | 0.0072 |
| MI Myocardial Infarction; TIA Transient Ischemic Attack; CHD Coronary Heart Disease; CVD Cerebrovascular disease | | | | | | | | | | | |

Table 24: Probability of cardiovascular event conditional on age and status of previous event (age 65-74)

| **Age 65-74** | | To | | | | | | | | | |
| --- | --- | --- | --- | --- | --- | --- | --- | --- | --- | --- | --- |
|  |  | Stable angina | Unstable angina 1 | Unstable angina 2 | MI 1 | MI 2 | TIA | Stroke 1 | Stroke 2 | CHD death | CVD death |
|  |  |  |  |  |  |  |  |  |  |  |  |
| From | Stable angina | 0.9760 | 0.0060 | 0 | 0.0110 | 0 | 0 | 0 | 0 | 0.0070 | 0 |
|  | Unstable angina (1^st^ yr) | 0 | 0 | 0.8144 | 0.0479 | 0 | 0 | 0 | 0 | 0.1319 | 0.0059 |
|  | Unstable angina (subsequent) | 0 | 0 | 0.9021 | 0.0844 | 0 | 0 | 0 | 0 | 0.0129 | 0.0006 |
|  | MI (1^st^ yr) | 0 | 0 | 0 | 0.0948 | 0.8106 | 0 | 0.0098 | 0 | 0.0811 | 0.0036 |
|  | MI (subsequent) | 0 | 0 | 0 | 0.0183 | 0.9585 | 0 | 0.0032 | 0 | 0.0191 | 0.0008 |
|  | TIA | 0 | 0 | 0 | 0.0055 | 0 | 0.9174 | 0.0423 | 0 | 0.0185 | 0.0163 |
|  | Stroke (1^st^ yr) | 0 | 0 | 0 | 0.0055 | 0 | 0 | 0.0485 | 0.8673 | 0.0393 | 0.0393 |
|  | Stroke (subsequent) | 0 | 0 | 0 | 0.0055 | 0 | 0 | 0.0237 | 0.9412 | 0.0148 | 0.0148 |
| MI Myocardial Infarction; TIA Transient Ischemic Attack; CHD Coronary Heart Disease; CVD Cerebrovascular disease | | | | | | | | | | | |

Table 25: Probability of cardiovascular event conditional on age and status of previous event (age 75-84)

| **Age 75-84** | | To | | | | | | | | | |
| --- | --- | --- | --- | --- | --- | --- | --- | --- | --- | --- | --- |
|  |  | Stable angina | Unstable angina 1 | Unstable angina 2 | MI 1 | MI 2 | TIA | Stroke 1 | Stroke 2 | CHD death | CVD death |
|  |  |  |  |  |  |  |  |  |  |  |  |
| From | Stable angina | 0.9680 | 0.0087 | 0 | 0.0163 | 0 | 0 | 0 | 0 | 0.0070 | 0 |
|  | Unstable angina (1^st^ yr) | 0 | 0 | 0.7366 | 0.0448 | 0 | 0 | 0 | 0 | 0.2093 | 0.0093 |
|  | Unstable angina (subsequent) | 0 | 0 | 0.8360 | 0.1484 | 0 | 0 | 0 | 0 | 0.0149 | 0.0007 |
|  | MI (1^st^ yr) | 0 | 0 | 0 | 0.0794 | 0.7502 | 0 | 0.0200 | 0 | 0.1440 | 0.0064 |
|  | MI (subsequent) | 0 | 0 | 0 | 0.0171 | 0.9466 | 0 | 0.0066 | 0 | 0.0286 | 0.0013 |
|  | TIA | 0 | 0 | 0 | 0.0082 | 0 | 0.8514 | 0.0878 | 0 | 0.0185 | 0.0342 |
|  | Stroke (1^st^ yr) | 0 | 0 | 0 | 0.0082 | 0 | 0 | 0.0471 | 0.7736 | 0.0856 | 0.0856 |
|  | Stroke (subsequent) | 0 | 0 | 0 | 0.0082 | 0 | 0 | 0.0251 | 0.9107 | 0.0280 | 0.0280 |
| MI Myocardial Infarction; TIA Transient Ischemic Attack; CHD Coronary Heart Disease; CVD Cerebrovascular disease | | | | | | | | | | | |

Table 26: Probability of cardiovascular event conditional on age and status of previous event (age 85-94)

| **Age 85-94** | | To | | | | | | | | | |
| --- | --- | --- | --- | --- | --- | --- | --- | --- | --- | --- | --- |
|  |  | Stable angina | Unstable angina 1 | Unstable angina 2 | MI 1 | MI 2 | TIA | Stroke 1 | Stroke 2 | CHD death | CVD death |
|  |  |  |  |  |  |  |  |  |  |  |  |
| From | Stable angina | 0.9600 | 0.0114 | 0 | 0.0216 | 0 | 0 | 0 | 0 | 0.0070 | 0 |
|  | Unstable angina (1^st^ yr) | 0 | 0 | 0.6315 | 0.0396 | 0 | 0 | 0 | 0 | 0.3149 | 0.0140 |
|  | Unstable angina (subsequent) | 0 | 0 | 0.7255 | 0.2568 | 0 | 0 | 0 | 0 | 0.0170 | 0.0008 |
|  | MI (1^st^ yr) | 0 | 0 | 0 | 0.0623 | 0.6498 | 0 | 0.0380 | 0 | 0.2393 | 0.0106 |
|  | MI (subsequent) | 0 | 0 | 0 | 0.0148 | 0.9311 | 0 | 0.0124 | 0 | 0.0399 | 0.0018 |
|  | TIA | 0 | 0 | 0 | 0.0108 | 0 | 0.7967 | 0.1286 | 0 | 0.0185 | 0.0453 |
|  | Stroke (1^st^ yr) | 0 | 0 | 0 | 0.0108 | 0 | 0 | 0.0409 | 0.6153 | 0.1665 | 0.1665 |
|  | Stroke (subsequent) | 0 | 0 | 0 | 0.0108 | 0 | 0 | 0.0248 | 0.8655 | 0.0494 | 0.0494 |
| MI Myocardial Infarction; TIA Transient Ischemic Attack; CHD Coronary Heart Disease; CVD Cerebrovascular disease | | | | | | | | | | | |

### Congestive Heart Failure

The review of previous economic evaluations of diabetes prevention cost-effectiveness studies found that only a small number of models had included congestive heart failure as a separate outcome. Discussion with the stakeholder group identified that the UKPDS Outcomes model would be an appropriate risk model for congestive heart failure in type 2 diabetes patients. However, it was suggested that this would not be an appropriate risk equation for individuals with normal glucose tolerance or impaired glucose tolerance. The Framingham risk equation was suggested as an alternative. As described above, switching from the framgingam risk score to the UKPDS was not possible due to differences in covariate selection. The main limitations of this equation is that it is quite old, based on a non-UK population, and include diabetes as a discrete health state rather than on a continuous scale. However, a citation search of this article did not identify a more recent or UK based alternative.

Congestive heart failure was included as a separate cardiovascular event because it was not included as an outcome of the QRISK2. The Framingham Heart Study has reported logistic regressions to estimate the 4 year probability of congestive heart failure for men and women (27). The equations included age, diabetes diagnosis (either formal diagnosis or a HbA1c>48 mmol/mol (6.5%)), BMI and systolic blood pressure to adjust risk based on individual characteristics. We used this risk equation to estimate the probability of congestive heart failure in the SPHR diabetes prevention model. Table 27 describes the covariates for the logit models to estimate the probability of congestive heart failure in men and women.

Table 27: Logistic regression coefficients to estimate the 4-year probability of congestive heart failure from the Framingham study

| **Variables** | **Units** | **Regression**  **Coefficient** | **OR (95% CI)** | **P** |
| --- | --- | --- | --- | --- |
| Men | | | | |
| Intercept |  | -9.2087 |  |  |
| Age | 10 y | 0.0412 | 1.51 (1.31-1.74) | <.001 |
| Left ventricular hypertrophy | Yes/no | 0.9026 | 2.47 (1.31-3.77) | <.001 |
| Heart rate | 10 bpm | 0.0166 | 1.18 (1.08-1.29) | <.001 |
| Systolic blood pressure | 20 mm Hg | 0.00804 | 1.17 (1.04-1.32) | 0.007 |
| Congenital heart disease | Yes/no | 1.6079 | 4.99 (3.80-6.55) | <.001 |
| Valve disease | Yes/no | 0.9714 | 2.64 (1.89-3.69) | <.001 |
| Diabetes | Yes/no | 0.2244 | 1.25 (0.89-1.76) | 0.2 |
| Women | | | | |
| Intercept |  | -10.7988 |  |  |
| Age | 10 y | 0.0503 | 1.65 (1.42-1.93) | <.001 |
| left ventricular hypertrophy | Yes/no | 1.3402 | 3.82 (2.50-5.83) | <.001 |
| Heart rate | 100 cL | 0.0105 | 1.11 (1.01-1.23) | 0.03 |
| Systolic blood pressure | 10 bpm | 0.00337 | 1.07 (0.96-1.20) | 0.24 |
| congenital heart disease | 20 mm Hg | 1.5549 | 4.74 (3.49-6.42) | <.001 |
| Valve disease | Yes/no | 1.3929 | 4.03 (2.86-5.67) | <.001 |
| Diabetes | Yes/no | 1.3857 | 4.00 (2.78-5.74) | <.001 |
| BMI | kg/m2 | 0.0578 | 1.06 (1.03-1.09) | <.001 |
| Valve disease and diabetes | Yes/no | -0.986 | 0.37 (0.18-0.78) | 0.009 |
| *OR indicates odds ratio; CI, confidence interval; LVH, left ventricular hypertrophy; CHD, congenital heart disease; and BMI, body mass index. Predicted probability of heart failure can be calculated as: p = 1/(1+exp(-xbeta)), where xbeta = Intercept + Sum (of regression coefficient*value of risk factor) | | | | |

Many of the risk factors included in this risk equation were not simulated in the diabetes model, therefore they could not be included in the model to predict CHD. We adjusted the baseline odds of CHD to reflect the expected prevalence of these symptoms in a UK population.

The proportion of the UK population with left ventricular hypertrophy was assumed to be 5% in line with previous analyses of the Whitehall II cohort (28). The heart rate for men was assumed to be 63.0bpm and for women 65.6bpm based on data from previous Whitehall II cohort analyses (29). The prevalence of congenital heart disease was estimated from an epidemiology study in the North of England. The study reports the prevalence of congenital heart disease among live births which was used to estimate the adult prevalence (30). This may over-estimate the prevalence, because the life expectancy of births with congenital heart disease is reduced compared with the general population. However, given the low prevalence it is unlikely to impact on the results. The prevalence of valve disease was estimated from the Echocardiographic Heart of England Screening study (31).

Using the estimated population values we adjusted the intercept values to account for the population risk in men and women. This resulted in a risk equation with age, systolic blood pressure, diabetes, and BMI in women to describe the risk of congestive heart failure for the policy analysis model.

## Microvascular Complications

The review of previous economic evaluations identified that the UKPDS data was commonly used to estimate the incidence of microvascular complications (1). This data has the advantage of being estimated from a UK diabetic population. Given that the events described in the UKPDS outcomes model are indicative of late stage microvascular complications, we did not believe it was necessary to seek an alternative model that would be representative of an impaired glucose tolerance population.

We adopted a simple approach to modelling microvascular complications. We used both versions of the UKPDS Outcomes model to estimate the occurrence of major events relating to these complications, including renal failure, amputation, foot ulcer, and blindness (12;20). These have the greatest cost and utility impact compared with earlier stages of microvascular complications, so are more likely to have an impact on the SPHR diabetes prevention outcomes. As a consequence, we assumed that microvascular complications only occur in individuals with HbA1c>48 mmol/mol (6.5%). Whilst some individuals with hyperglycaemia (HbA1c>42 mmol/mol (6.5%)) may be at risk of developing microvascular complications, it is unlikely that they will progress to renal failure, amputation or blindness before a diagnosis of diabetes. Importantly, we did not assume that only individuals who have a formal diagnosis of diabetes are at risk of these complications. This allows us to incorporate the costs of undetected diabetes into the simulation.

The UKPDS includes four statistical models to predict foot ulcers, amputation with no prior ulcer, amputation with prior ulcer and a second amputation (20). In order to simplify the simulation of neuropathy outcomes we consolidated the models for first amputation with and without prior ulcer into a single equation. The parametric survival models were used to generate estimates of the cumulative hazard in the current and previous period. From which the probability of organ damage being diagnosed was estimated.

|  | $p\left( Death \right)=1-exp(H\left( t \right)-H\left( t-1 \right))$ |  |
| --- | --- | --- |

The functional form for the microvascular models included exponential and Weibull. The logistic model was also used to estimate the probability of an event over the annual time interval.

### Retinopathy

We used the UKPDS outcomes model v2 to estimate the incidence of blindness in individuals with HbA1c>48 mmol/mol (6.5%). The exponential model assumes a baseline hazard $\lambda$, which can be calculated from the model coefficients reported in Table 28 and the individual characteristics for $\boldsymbol{X}$.

$$\lambda=exp\left( \beta_{0}\boldsymbol{+X}\boldsymbol{\beta}_{\boldsymbol{k}} \right)$$

Table 28: Parameters of the UKPDS2 Exponential Blindness survival model

|  | **Mean coefficient** | **Standard error** | **Modified mean coefficient** |
| --- | --- | --- | --- |
| Lambda | -11.607 | 0.759 | -10.967 |
| Age at diagnosis | 0.047 | 0.009 | 0.047 |
| HbA1c | 0.171 | 0.032 | 0.171 |
| Heart rate | 0.080 | 0.039 |  |
| SBP | 0.068 | 0.032 | 0.068 |
| White Blood Count | 0.052 | 0.019 |  |
| CHF History | 0.841 | 0.287 | 0.841 |
| IHD History | 0.0610 | 0.208 | 0.061 |

The age at diagnosis coefficient was multiplied by age in the current year if the individual had not been diagnosed with diabetes or by the age at diagnosis if the individual had received a diagnosis. The expected values for the risk factors not included in the SPHR model (heart rate and white blood count) were taken from Figure 3 of the UKPDS publication in which these are described (20). Assuming these mean values, it was possible to modify the baseline risk without simulating heart rate and white blood cell count.

### Neuropathy

We used the UKPDS outcomes model v2 to estimate the incidence of ulcer and amputation in individuals with HbA1c>48 mmol/mol (6.5%). The parameters of the ulcer and first amputation models are reported in Table 29.

Table 29: Parameters of the UKPDS2 Exponential model for Ulcer, Weibull model for first amputation with no prior ulcer and exponential model for 1^st^ amputation with prior ulcer

|  | Ulcer | | 1^st^ Amputation no prior ulcer | | 1^st^ Amputation prior ulcer | | 2^nd^ Amputation | |
| --- | --- | --- | --- | --- | --- | --- | --- | --- |
|  | Logistic | | Weibull | | Exponential | | Exponential | |
|  | Mean | Standard error | Mean | Standard error | Mean | Standard error | Mean | Standard error |
| lambda | -11.295 | 1.130 | -14.844 | 1.205 | -0.881 | 1.39 | -3.455 | 0.565 |
| Rho |  |  | 2.067 | 0.193 |  |  |  |  |
| Age at diagnosis | 0.043 | 0.014 | 0.023 | 0.011 | -0.065 | 0.027 |  |  |
| Female | -0.962 | 0.255 | -0.0445 | 0.189 |  |  |  |  |
| Atrial fibrillation |  |  | 1.088 | 0.398 |  |  |  |  |
| BMI | 0.053 | 0.019 |  |  |  |  |  |  |
| HbA1c | 0.160 | 0.056 | 0.248 | 0.042 |  |  | 0.127 | 0.06 |
| HDL |  |  | -0.059 | 0.032 |  |  |  |  |
| Heart rate |  |  | 0.098 | 0.050 |  |  |  |  |
| MMALB |  |  | 0.602 | 0.180 |  |  |  |  |
| PVD | 0.968 | 0.258 | 1.010 | 0.189 | 1.769 | 0.449 |  |  |
| SBP |  |  | 0.086 | 0.043 |  |  |  |  |
| WBC |  |  | 0.040 | 0.017 |  |  |  |  |
| Stroke History |  |  | 1.299 | 0.245 |  |  |  |  |

The exponential model assumes a baseline hazard $\lambda$, which can be calculated from the model coefficients reported in Table 29 and the individual characteristics for $\boldsymbol{X}$.

$$\lambda=exp\left( \beta_{0}\boldsymbol{+X\beta} \right)$$

The Weibull model for amputation assumes a baseline hazard:

$$h\left( t \right)=\rho t^{\rho-1}exp(\lambda)$$

where $\lambda$is also conditional on the coefficients and individual characteristics at time t. The logistic model for ulcer is described below.

$$\Pr\left( y=1 | \mathbf{X} \right)=\frac{\exp(\mathbf{X}\boldsymbol{\beta})}{1+exp(\mathbf{X}\boldsymbol{\beta}))}$$

The ulcer and amputation models include a number of covariates that were not included in the simulation. As such it was necessary to adjust the statistical models to account for these measures. We estimated a value for the missing covariates and added the value multiplied by the coefficient to the baseline hazard.

The expected values for the risk factors not included in the SPHR model (heart rate, white blood count, micro-/macroalbuminurea, peripheral vascular disease and atrial fibrillation) were taken from Figure 3 of the UKPDS publication in which these are described (20). In the ulcer model we assumed that 2% of the population had peripheral vascular disease.

The amputation risk model with a history of ulcer was not included in the simulation, but was used to estimate an additional log hazard ratio to append onto the amputation model without a history of ulcer. The log hazard was estimated for each model assuming the same values for other covariates. The difference in the log hazard between the two models was used to approximate the log hazard ratio for a history of ulcer in the amputation model (10.241). The final model specifications are reported in Table 30.

Table 30: Coefficients estimates for Ulcer and 1^st^ Amputation

|  | **Ulcer** | | **1^st^ Amputation** | | **2^nd^ Amputation** | |
| --- | --- | --- | --- | --- | --- | --- |
|  | Logistic | | Weibull | | Exponential | |
|  | Mean | Standard error | Mean | Standard error | Mean | Standard error |
| Lambda | -11.276 | 1.13 | -13.954 | 1.205 | -3.455 | 0.565 |
| Rho |  |  | 2.067 | 0.193 |  |  |
| Age at Diagnosis | 0.043 | 0.014 | 0.023 | 0.011 |  |  |
| Female | -0.962 | 0.255 | -0.445 | 0.189 |  |  |
| BMI | 0.053 | 0.019 |  |  |  |  |
| HbA1c | 0.160 | 0056 | 0.248 | 0.042 | 0.127 | 0.06 |
| HDL |  |  | -0.059 | 0.032 |  |  |
| Stroke |  |  | 1.299 | 0.245 |  |  |
| Foot Ulcer |  |  | 10.241 |  |  |  |

### Nephropathy

We used the UKPDS outcomes model v1 to estimate the incidence of renal failure in individuals with HbA1c>48 mmol/mol (6.5%). Early validation analyses identified that the UKPDS v2 model implements in the SPHR model substantially overestimated the incidence of renal failure. The Weibull model for renal failure assumes a baseline hazard:

$$h\left( t \right)=\rho t^{\rho-1}exp(\lambda)$$

where $\lambda$is also conditional on the coefficients and individual characteristics at time t. The parameters of the renal failure risk model are reported in Table 31.

Table 31: Parameters of the UKPDS2 Weibull renal failure survival model

|  | **Mean** | **Standard error** |
| --- | --- | --- |
| Lambda | -10.016 | 0.939 |
| Shape parameter | 1.865 | 0.387 |
| SBP | 0.404 | 0.106 |
| BLIND History | 2.082 | 0.551 |

## Cancer

The conceptual model identified breast cancer and colorectal cancer risk as being related to BMI. However, these outcomes were not frequently included in previous cost-effectiveness models for diabetes prevention. Discussion with stakeholders identified the EPIC Norfolk epidemiology cohort study as a key source of information about cancer risk in a UK population. Therefore, we searched publications from this cohort to identify studies reporting the incidence of these risks. In order to obtain the best quality evidence for the relationship between BMI and cancer risk we searched for a recent systematic review and meta-analysis using key terms ‘Body Mass Index’ and ‘Cancer’, filtering for meta-analysis studies.

### Breast cancer

Incidence rates for breast cancer in the UK were estimated from the European Prospective Investigation of Cancer (EPIC) cohort. This is a large multi-centre cohort study looking at diet and cancer. In 2004 the UK incidence of breast cancer by menopausal status was reported in a paper from this study investigating the relationship between body size and breast cancer (32). The estimates of the breast cancer incidence in the UK are reported in Table 32.

Table 32: UK breast cancer incidence

|  | **Number of Cases** | **Person Years** | **Mean BMI** | **Incidence Rate of per person-year** | **Reference** |
| --- | --- | --- | --- | --- | --- |
| UK pre-menopause | 102 | 103114.6 | 24 | 0.00099 | (32) |
| UK post-menopause | 238 | 84214.6 | 24 | 0.00283 | (32) |

A large meta-analysis that included 221 prospective observational studies has reported relative risks of cancers per unit increase in BMI, including breast cancer by menopausal status (33). We included a risk adjustment in the model so that individuals with higher BMI have a higher probability of pre-and post-menopausal breast cancer (33). In the simulation we adjusted the incidence of breast cancer by multiplying the linear relative risk by the difference in the individual’s BMI and the average BMI reported in the EPIC cohort. The relative risk and confidence intervals per 5mg/m^2^ increase in BMI are reported in Table 33.

Table 33: Relative risk of Breast cancer by BMI

|  | **Mean Relative risk** | **2.5^th^ Confidence Interval** | **97.5^th^ Confidence Interval** | **Reference** |
| --- | --- | --- | --- | --- |
| UK pre-menopause | 0.89 | 0.84 | 0.94 | (33) |
| UK post-menopause | 1.09 | 1.04 | 1.14 | (33) |

### Colorectal cancer

Incidence rates for colorectal cancer in the UK were reported from the European Prospective Investigation of Cancer (EPIC) cohort. The UK incidence of colorectal cancer is reported by gender in a paper from this study investigating the relationship between body size and colon and rectal cancer (32). The estimates of the colorectal cancer incidence are reported in Table 34.

Table 34: UK colorectal cancer incidence

|  | **Number of Cases** | **Person Years** | **Mean Age** | **Mean BMI** | **Incidence Rate of per person-year** | **Reference** |
| --- | --- | --- | --- | --- | --- | --- |
| Male | 125 | 118468 | 53.1 | 25.4 | 0.00106 | (34) |
| Female | 145 | 277133 | 47.7 | 24.5 | 0.00052 | (34) |

The risk of colorectal cancer has been linked to obesity. We included a risk adjustment in the model to reflect observations that the incidence of breast cancer is increased in individuals with higher BMI. A large meta-analysis that included 221 prospective observational studies has reported relative risks of BMI and cancers, including colon cancer by gender (33). We selected linear relative risk estimates estimated from pooled European and Australian populations. In the simulation we adjusted the incidence of colorectal cancer by multiplying the relative risk by the difference in the individual’s BMI and the average BMI reported in the EPIC cohort. The relative risk and confidence intervals per 5mg/m^2^ increase in BMI are reported in Table 35.

Table 35: Relative risk of colon cancer by BMI

|  | **Mean Relative risk** | **2.5^th^ Confidence Interval** | **97.5^th^ Confidence Interval** | **Reference** |
| --- | --- | --- | --- | --- |
| UK pre-menopause | 1.21 | 1.18 | 1.24 | (33) |
| UK post-menopause | 1.04 | 1 | 1.07 | (33) |

## Osteoarthritis

The stakeholder group requested that BMI and diabetes be included as independent risk factors for osteoarthritis based on recent evidence (5). Osteoarthritis had not been included as a health state in previous cost-effectiveness models. A search for studies using key words ‘Diabetes’, ‘Osteoarthritis’ and ‘Cohort Studies’ did not identify a UK based study with diabetes and BMI included as independent covariates in the risk model. The Bruneck cohort, a longitudinal study of inhabitants of a town in Italy reported diabetes and BMI as independent risk factors for osteoarthritis (5). The cohort may not be representative of the UK. However, the individuals are from a European country, the study has a large sample size and has estimated the independent effects of BMI and diabetes on the risk of osteoarthritis. No UK based studies identified in our searches met these requirements. We did not identify any studies that described diabetes risk on a continuous scale. The data used to estimate the incidence of osteoarthritis is reported in Table 36.

Table 36: Incidence of osteoarthritis and estimated risk factors

|  | No cases | Person years | Mean BMI | Incidence rate | Reference |
| --- | --- | --- | --- | --- | --- |
| No diabetes | 73 | 13835 | 24.8 | 0.0053 | (5) |
|  | Hazard ratio | 2.5th | 97.5th |  | Reference |
| HR Diabetes | 2.06 | 1.11 | 3.84 |  | (5) |
| HR BMI | 1.076 | 1.023 | 1.133 |  | (5) Personal communication |

## Depression

Depression was not included as a health state in previous cost-effectiveness models for diabetes prevention. However, a member of the stakeholder group identified that a relationship between diabetes and depression was included in the CORE diabetes treatment model (35). With this in mind, we decided to include depression as a health state in the model, but not to model its severity.

Some individuals enter the simulation with depression at baseline according to individual responses in the Health Survey for England 2011 questionnaire. Depression is described as a chronic state from which individuals do not completely remit. We did not estimate the effect of depression on the longitudinal changes for BMI, glycaemia, systolic blood pressure and cholesterol. As a consequence it was not possible to relate the impact of depression to the incidence of diabetes and cardiovascular risk.

In the simulation, individuals can develop depression in any cycle of the model. The baseline incidence of depression among all individuals without a history of depression was estimated from a study examining the bidirectional association between depressive symptoms and type 2 diabetes (36). Although the study was not from a UK population, the US cohort included ethnically diverse men and women aged 45 to 84 years. We assumed that diagnosis of diabetes and/or cardiovascular disease increases the incidence of depression in individuals who do not have depression at baseline. We identified a method for inflating risk of depression for individuals with diabetes from the US cohort study described above (36). The risk of depression in individuals who have had a stroke was also inflated according to a US cohort study (37). Odds of depression and odds ratios for inflated risk of depression due to diabetes or stroke are presented in Table 37.

Table 37: Baseline incidence of depression

| Baseline Risk of depression | | | |
| --- | --- | --- | --- |
|  | Mean | 2.5^th^ CI | 97.5th |
| Depression cases in NGT | 336 |  |  |
| Person years | 9139 |  |  |
| Odds of depression | 0.0382 |  |  |
| Log odds of depression | -3.266 |  |  |
| Inflated risk for Diabetes | | | |
| Odds ratio of diabetes | 1.52 | 1.09 | 2.12 |
| Log odds ratio of diabetes | 0.419 |  |  |
| Inflate risk of stroke | | | |
| Odds ratio of stroke | 6.3 | 1.7 | 23.2 |
| Log odds ratio stroke | 1.8406 |  |  |
| NGT Normal Glucose Tolerance | | | |

## Mortality

### Cardiovascular Mortality

Cardiovascular mortality is included as an event within the QRISK2 and the probability of subsequent cardiovascular events obtained from an HTA assessing statins (14) as described in the cardiovascular disease section above.

### Cancer Mortality

Cancer mortality rates were obtained from the Office of National statistics (38). The ONS report one and five year net survival rates for various cancer types, by age group and gender. Net survival was an estimate of the probability of survival from the cancer alone. It can be interpreted as the survival of cancer patients after taking into account the background mortality that the patients would have experienced if they had not had cancer.

The age-adjusted 5-year survival rate for breast cancer and colorectal cancer were used to estimate an annual risk of mortality assuming a constant rate of mortality. We assume that the mortality rate does not increase due to cancer beyond 5 years after cancer diagnosis. The five year survival rate for breast cancer is 84.3%, which translated into a 3.37% annual probability of death from breast cancer. The five year survival rate for persons with colorectal cancer is 55.3%, which translated into an 11.16% annual probability of death from colorectal cancer.

#### Other cause Mortality (including diabetes risk)

Other cause mortality describes the risk of death from any cause except cardiovascular disease and cancer. All-cause mortality rates by age and sex were extracted from the Office of National Statistics (39). The mortality statistics report the number of deaths by ICD codes for 5-year age groups. We subtracted the number of cardiovascular disease, breast and colorectal cancer related deaths from the all-cause mortality total to estimate other cause mortality rates by age and sex (Table 35).

Table 38: All cause and derived other cause mortality from the Office of National statistics

|  | All cause | All cause | Other cause | Other cause |  | All cause | All cause | Other cause | Other cause |
| --- | --- | --- | --- | --- | --- | --- | --- | --- | --- |
|  | Men | Women | Men | Women |  | Men | Women | Men | Women |
| 1 | 0.0004 | 0.0003 | 0.0003 | 0.0003 | 51 | 0.0034 | 0.0024 | 0.0025 | 0.0017 |
| 2 | 0.0002 | 0.0002 | 0.0002 | 0.0002 | 52 | 0.0039 | 0.0026 | 0.0029 | 0.0019 |
| 3 | 0.0001 | 0.0001 | 0.0001 | 0.0001 | 53 | 0.0044 | 0.0028 | 0.0032 | 0.0020 |
| 4 | 0.0001 | 0.0001 | 0.0001 | 0.0001 | 54 | 0.0045 | 0.0032 | 0.0034 | 0.0022 |
| 5 | 0.0001 | 0.0001 | 0.0001 | 0.0001 | 55 | 0.0051 | 0.0033 | 0.0037 | 0.0024 |
| 6 | 0.0001 | 0.0001 | 0.0001 | 0.0001 | 56 | 0.0057 | 0.0037 | 0.0041 | 0.0027 |
| 7 | 0.0001 | 0.0001 | 0.0001 | 0.0000 | 57 | 0.0061 | 0.0041 | 0.0044 | 0.0030 |
| 8 | 0.0001 | 0.0001 | 0.0001 | 0.0000 | 58 | 0.0069 | 0.0041 | 0.0050 | 0.0030 |
| 9 | 0.0001 | 0.0001 | 0.0001 | 0.0001 | 59 | 0.0071 | 0.0050 | 0.0052 | 0.0036 |
| 10 | 0.0001 | 0.0000 | 0.0001 | 0.0000 | 60 | 0.0081 | 0.0054 | 0.0059 | 0.0040 |
| 11 | 0.0001 | 0.0001 | 0.0001 | 0.0001 | 61 | 0.0086 | 0.0057 | 0.0063 | 0.0042 |
| 12 | 0.0001 | 0.0001 | 0.0001 | 0.0001 | 62 | 0.0096 | 0.0062 | 0.0070 | 0.0046 |
| 13 | 0.0001 | 0.0001 | 0.0001 | 0.0001 | 63 | 0.0104 | 0.0067 | 0.0076 | 0.0050 |
| 14 | 0.0001 | 0.0001 | 0.0001 | 0.0001 | 64 | 0.0108 | 0.0072 | 0.0079 | 0.0053 |
| 15 | 0.0002 | 0.0001 | 0.0002 | 0.0001 | 65 | 0.0125 | 0.0082 | 0.0091 | 0.0061 |
| 16 | 0.0002 | 0.0001 | 0.0002 | 0.0001 | 66 | 0.0141 | 0.0090 | 0.0103 | 0.0067 |
| 17 | 0.0003 | 0.0002 | 0.0003 | 0.0002 | 67 | 0.0148 | 0.0097 | 0.0108 | 0.0072 |
| 18 | 0.0004 | 0.0002 | 0.0004 | 0.0002 | 68 | 0.0162 | 0.0107 | 0.0118 | 0.0079 |
| 19 | 0.0004 | 0.0002 | 0.0004 | 0.0002 | 69 | 0.0181 | 0.0118 | 0.0132 | 0.0087 |
| 20 | 0.0005 | 0.0002 | 0.0005 | 0.0002 | 70 | 0.0218 | 0.0138 | 0.0157 | 0.0101 |
| 21 | 0.0005 | 0.0002 | 0.0005 | 0.0002 | 71 | 0.0234 | 0.0145 | 0.0168 | 0.0106 |
| 22 | 0.0005 | 0.0002 | 0.0005 | 0.0002 | 72 | 0.0252 | 0.0167 | 0.0182 | 0.0122 |
| 23 | 0.0005 | 0.0002 | 0.0005 | 0.0002 | 73 | 0.0269 | 0.0173 | 0.0193 | 0.0127 |
| 24 | 0.0005 | 0.0002 | 0.0005 | 0.0002 | 74 | 0.0310 | 0.0200 | 0.0223 | 0.0147 |
| 25 | 0.0006 | 0.0003 | 0.0006 | 0.0002 | 75 | 0.0327 | 0.0222 | 0.0233 | 0.0157 |
| 26 | 0.0006 | 0.0003 | 0.0005 | 0.0002 | 76 | 0.0375 | 0.0249 | 0.0267 | 0.0176 |
| 27 | 0.0006 | 0.0004 | 0.0005 | 0.0003 | 77 | 0.0411 | 0.0284 | 0.0293 | 0.0202 |
| 28 | 0.0007 | 0.0003 | 0.0006 | 0.0003 | 78 | 0.0458 | 0.0321 | 0.0326 | 0.0228 |
| 29 | 0.0007 | 0.0003 | 0.0006 | 0.0003 | 79 | 0.0523 | 0.0358 | 0.0372 | 0.0254 |
| 30 | 0.0007 | 0.0004 | 0.0006 | 0.0003 | 80 | 0.0585 | 0.0411 | 0.0418 | 0.0289 |
| 31 | 0.0008 | 0.0004 | 0.0007 | 0.0004 | 81 | 0.0652 | 0.0456 | 0.0465 | 0.0321 |
| 32 | 0.0007 | 0.0005 | 0.0007 | 0.0004 | 82 | 0.0745 | 0.0530 | 0.0531 | 0.0372 |
| 33 | 0.0008 | 0.0005 | 0.0007 | 0.0004 | 83 | 0.0833 | 0.0606 | 0.0594 | 0.0426 |
| 34 | 0.0009 | 0.0005 | 0.0008 | 0.0004 | 84 | 0.0931 | 0.0678 | 0.0664 | 0.0476 |
| 35 | 0.0010 | 0.0006 | 0.0008 | 0.0005 | 85 | 0.1040 | 0.0760 | 0.0738 | 0.0537 |
| 36 | 0.0011 | 0.0006 | 0.0010 | 0.0005 | 86 | 0.1147 | 0.0872 | 0.0814 | 0.0617 |
| 37 | 0.0013 | 0.0006 | 0.0011 | 0.0005 | 87 | 0.1300 | 0.0977 | 0.0923 | 0.0692 |
| 38 | 0.0013 | 0.0007 | 0.0011 | 0.0006 | 88 | 0.1468 | 0.1106 | 0.1042 | 0.0782 |
| 39 | 0.0013 | 0.0007 | 0.0011 | 0.0006 | 89 | 0.1643 | 0.1242 | 0.1166 | 0.0879 |
| 40 | 0.0015 | 0.0009 | 0.0012 | 0.0006 | 90 | 0.2285 | 0.1982 | 0.1660 | 0.1425 |
| 41 | 0.0016 | 0.0010 | 0.0013 | 0.0007 | 91 | 0.2285 | 0.1982 | 0.1660 | 0.1425 |
| 42 | 0.0018 | 0.0010 | 0.0015 | 0.0008 | 92 | 0.2285 | 0.1982 | 0.1660 | 0.1425 |
| 43 | 0.0018 | 0.0012 | 0.0015 | 0.0009 | 93 | 0.2285 | 0.1982 | 0.1660 | 0.1425 |
| 44 | 0.0020 | 0.0012 | 0.0017 | 0.0009 | 94 | 0.2285 | 0.1982 | 0.1660 | 0.1425 |
| 45 | 0.0022 | 0.0014 | 0.0017 | 0.0010 | 95 | 0.2285 | 0.1982 | 0.1751 | 0.1509 |
| 46 | 0.0023 | 0.0016 | 0.0018 | 0.0011 | 96 | 0.2285 | 0.1982 | 0.1751 | 0.1509 |
| 47 | 0.0023 | 0.0015 | 0.0018 | 0.0011 | 97 | 0.2285 | 0.1982 | 0.1751 | 0.1509 |
| 48 | 0.0027 | 0.0017 | 0.0021 | 0.0012 | 98 | 0.2285 | 0.1982 | 0.1751 | 0.1509 |
| 49 | 0.0028 | 0.0019 | 0.0022 | 0.0014 | 99 | 0.2285 | 0.1982 | 0.1751 | 0.1509 |
| 50 | 0.0030 | 0.0021 | 0.0023 | 0.0015 | 100 | 0.2285 | 0.1982 | 0.1751 | 0.1509 |

The rate of other cause mortality by age and sex was treated as the baseline hazard. Following input from stakeholders, an increased risk of mortality was assigned to individuals with diabetes using data from a published meta-analysis (40). This study used data from 820,900 people from 97 prospective studies to calculate hazard ratios for cause-specific death, according to baseline diabetes status (40). Cause of death was separated into vascular disease, cancer and other cause mortality. From this study we estimated that individuals with a diagnosis of diabetes have a fixed increased risk of other cause mortality (Hazard ratio 1.8 (95% CI 1.71-1.9)). The estimates reported in the meta-analysis include increased risk of death from renal disease, therefore mortality from renal disease was not simulated separately to avoid double counting of benefits.

# Direct Health Care Costs

At any given time period of the model individuals can have multiple health complications that incur direct healthcare costs. Some of the health states are mutually exclusive; however an individual can accrue multiple complications within the model. Each health state is associated with an average cost, which is accrued by all individuals for every time period for which the state is indicated. Resource use for each comorbidity is added together and no savings are assumed to be made from the use of the same resources for two or more comorbidities for an individual. An exception to this is an assumed adjustment to the utilisation of GP services for individuals with chronic diseases.

In some instances we have adopted costs and prices from old studies. We have inflated all prices and costs to 2013 prices using inflation indices reported in the Personal Social Services Research Unit (PSSRU) (41). This documents health related inflation up to 2014/15 prices.

Primary care and community care costs were sought from the Personal Social Services Research Unit (PSSRU) (41), and secondary care costs from UK reference costs (42). Drug costs were obtained from the British National Formulary (43). In most instances costs for long term health outcomes were sought from recent Health Technology Appraisals as this was thought to be the best source of evidence for costs and resource use by disease area in the UK. If an HTA appraisal were not identified, searches for good quality cost-effectiveness analyses for the relevant disease area were conducted to identify the appropriate UK costs.

## GP attendance

The costs of each visit to a General Practitioner were estimated at £46 from the Personal Social Services Research Unit (PSSRU) (41).

Diabetes diagnosis incurred a cost of £14 in line with costs used for a previous evaluation of a Diabetes Prevention Programme (44).

Recent guidelines for hypertension have recommended that hypertension be confirmed with ambulatory blood pressure monitoring (ABPM) (15). The cost of ABPM assessment is included in the cost of diagnosis (£53.40) (19), however, we assume that the test does not alter the initial diagnosis.

The cost of identifying individuals to receive statins is assumed to be negligible because cases are detected using existing cardiovascular risk programmes used by the GP.

## Diabetes

We were advised by stakeholders to model a simplified diabetes treatment pathway. It was recommended that a single annual cost of prescriptions be applied to all patients diagnosed with diabetes. Initially we explored this as an option but concluded that the timing of more costly treatments for type 2 diabetes is important because treatment costs will be discounted. The model assesses interventions that lower HbA1c and so have the potential to impact on the level of treatment required.

We decided to implement a three stage treatment regimen as a trade-off between model simplicity and capturing key cost differences between the interventions. At diagnosis all patients are prescribed low cost treatments, such as Metformin and Sulfonylurea. We chose Metformin, 500mg/day to describe the average cost of these medications. If HbA1c increases above a threshold the individual is prescribed a more expensive Gliptins in addition to Metformin. The individual continues to receive Metformin plus Gliptins for a period of time until they require insulin.

### Metformin Monotherapy

Cost estimates from the British National Formulary indicate that the cost of Metformin is approximately £19 per tablet. Other resource use costs and utilisation assumptions for diabetics receiving Metformin monotherapy are detailed in Table 39.

Table 39: Drug costs and resource utilisation costs for low cost diabetes monotherapy

| Resource | Assumption for costs | Unit cost | Source | Inflation | Annual utilisation | Source | Cost per year |
| --- | --- | --- | --- | --- | --- | --- | --- |
| Metformin | 500mg *bid* standard (85% of patients) or modified release (15%) tablets | £18.83 per annum | (43) | 1 | 1 | Assumption | £18.83 |
| Nurse at GP | Nurse advanced per surgery consultation with qualifications | £25.52 | (41) | 1 | 1 | Stakeholder workshop | £25.52 |
| Health care assistant | Clinical support worker patient work 10 mins | £3.40 | (41) | 1 | 1 | Stakeholder workshop | £3.40 |
| Urine sample | Biochemistry | £1 |  | 1 | 3 | Stakeholder workshop | £1 |
| Eye screening | Optometrist test 2006 price | £18.39 | (45) | 1.322 | 1 | Stakeholder workshop | £24.31 |
| HbA1c | Haematology | £3 |  | 1 | 1 | Stakeholder workshop | £3.00 |
| Lipids | Chemistry | £1 |  | 1 | 1 | Stakeholder workshop | £1.00 |
| Liver function | Chemistry | £1 |  | 1 | 1 | Stakeholder workshop | £1.00 |
| B12 | Chemistry | £1 |  | 1 | 1 | Stakeholder workshop | £1.00 |
|  | | | | | | | £79.06 |

The cost of diabetes in the year after diagnosis is assumed to be greater than subsequent years because the individual will receive more contact time whilst their diabetes is being controlled. The additional costs of diabetes in the year after diagnosis are reported in Table 40.

Table 40: Drug costs and resource utilisation costs for the first year of low cost diabetes treatment

| Resource | Assumption for costs | Unit cost | Source | Inflation | Annual utilisation | Source | Cost per year |
| --- | --- | --- | --- | --- | --- | --- | --- |
| Nurse at GP | Nurse advanced per surgery consultation with qualifications | £25.52 | (41) | 1 | 1 | Stakeholder workshop | £51.04 |
| Health care assistant | Clinical support worker patient work 10 mins | £3.40 | (41) | 1 | 1 | Stakeholder workshop | £6.80 |
| Urine sample | Biochemistry | £1 |  | 1 | 3 | Stakeholder workshop | £2 |
| HbA1c | Haematology | £3 |  | 1 | 1 | Stakeholder workshop | £6.00 |
| Lipids | Chemistry | £1 |  | 1 | 1 | Stakeholder workshop | £2.00 |
| Liver function | Chemistry | £1 |  | 1 | 1 | Stakeholder workshop | £2.00 |
| B12 | Chemistry | £1 |  | 1 | 1 | Stakeholder workshop | £2.00 |
|  | | | | | | | £103 |

### Metformin plus Gliptins

Simulated individuals experience an annual increase in HbA1c. Gillett et al. (2012) assume that individuals switch to dual treatment if HbA1c increases above 7.4% (6). Within the model, the individual is switched to a dual treatment in the first annual cycle in which HbA1c exceeds 7.4%. For costing purposes the second drug to be added to Metformin was Sitagliptin, which is reported in the British National Formulary to cost £1.41 per day. Belsey et al. (2009) report that 48% of patients used monitoring strips at a mean weekly consumption of 3.3 (46). Table 41 reports the other resource use costs and utilisation assumptions for diabetics receiving Metformin plus Gliptins.

Table 41: Drug costs and resource utilisation costs for Metformin and Gliptins

| Resource | Assumption for costs | Unit cost | Source | Inflation | Annual utilisation | Source | Cost per year |
| --- | --- | --- | --- | --- | --- | --- | --- |
| Sitagliptin | 100mg per day 28 tab pack | £1.19 | (43) | 1 | 360 | Assumption | £434 |
| Metformin | 500mg *bid* standard (85% of patients) or modified release (15%) tablets | £18.83 per annum | (43) | 1 | 1 | Assumption | £18.83 |
| Self-monitoring strips | 50 strip pack Active® | £0.20 | (43) | 1 | 82.20 | (46) | £16.36 |
| Nurse at GP | Nurse advanced per surgery consultation with qualifications | £25.52 | (41) | 1 | 1 | Stakeholder workshop | £25.00 |
| Health care assistant | Clinical support worker patient work 10 mins | £3.40 | (41) | 1 | 1 | Stakeholder workshop | £3.40 |
| Urine sample | Biochemistry | £1 |  | 1 | 1 | Stakeholder workshop | £1 |
| Eye screening | Optometrist test 2006 price | £18.39 | (45) | 1.322 | 1 | Stakeholder workshop | £24.31 |
| HbA1c | Haematology | £3 |  | 1 | 3 | Stakeholder workshop | £3.00 |
| Lipids | Chemistry | £1 |  | 1 | 3 | Stakeholder workshop | £1.00 |
| Liver function | Chemistry | £1 |  | 1 | 3 | Stakeholder workshop | £1.00 |
| B12 | Chemistry | £1 |  | 1 | 3 | Stakeholder workshop | £1.00 |
|  | | | | | | | £529 |

### Insulin plus Oral Anti-diabetics

The second major treatment change is assumed to be initiation of insulin. Gillett et al. (2012) assumed that individuals switch to insulin if HbA1c increases above 8.5% (6). Within the model the individual is switched to insulin in the first annual cycle at which HbA1c exceeds 8.5%. The insulin Glargine was chosen to represent insulin treatment in the UK and is consistent with Gillett et al. (2012) (6). Furthermore, recent cost studies from the UK have promoted the use of Glargine to reduce costs (47). The total resource use and costs of this health state are reported in Table 42 and Table 43.

Table 42: Costs of insulin treatment

|  | Price | Source |
| --- | --- | --- |
| Glargine | £628.44 | (47)(2006 prices) |
| Oral anti-diabetics | £43.68 | (47) (2006 prices) |
| Reagent test strips | £221.43 | (47) (2006 prices) |
| Hypoglycaemic rescue | £23.43 | (47) (2006 prices) |
| Pen delivery devices | £54.79 | (47) (2006 prices) |
| Sharps | £68.82 | (47) (2006 prices) |
| Total cost per year | £1,013.51 |  |

Table 43: Drug costs and resource utilisation costs for insulin and oral anti-diabetics

| **Resource** | **Assumption for costs** | **Unit cost** | **Source** | **Inflation (2013)** | **Annual utilisation** | **Source** | **Cost per year** |
| --- | --- | --- | --- | --- | --- | --- | --- |
| Insulin treatment costs | Total annual cost | £1,013.51 | (47) | 1.322 | NA | N/A | £1376 |
| Nurse at GP | Nurse advanced per surgery consultation with qualifications | £25 | (41) | 1 | 3 | Stakeholder workshop | £76.55 |
| Health care assistant | Clinical support worker patient work 10 mins | £3.40 | (41) | 1 | 3 | Stakeholder workshop | £10.20 |
| Urine sample | Biochemistry | £1 |  | 1 | 3 | Stakeholder workshop | £3.00 |
| Eye screening | Optometrist test 2006 price | £18.39 | (45) | 1.322 | 1 | Stakeholder workshop | £24.31 |
| HbA1c | Haematology | £3 |  | 1 | 3 | Stakeholder workshop | £9.00 |
| Lipids | Chemistry | £1 |  | 1 | 3 | Stakeholder workshop | £3.00 |
| Liver function | Chemistry | £1 |  | 1 | 3 | Stakeholder workshop | £3.00 |
| B12 | Chemistry | £1 |  | 1 | 3 | Stakeholder workshop | £3.00 |
|  | | | | | | | £1503 |

## Statins

We assumed that individuals who are prescribed statins receive a daily dose of 40mg of generic Simvastatin. The British National Formulary reports a cost of approximately 3p per day. The individual remains on statins for the rest of their life. Table 44 reports the derived annual costs for statins. We assumed that individual’s cholesterol is monitored whilst on statins and patients receive two lipid tests per year. The cost of GP attendance was not included in the cost of statins to avoid double counting of GP attendance.

Table 44: Annual treatment costs of statins

|  | **Assumption for costs** | **Unit cost** | **Source** | **Inflation** | **Annual utilisation** | **Cost per year** |
| --- | --- | --- | --- | --- | --- | --- |
| Statins | Simvastatin 20mg | £0.0325 | (43) | 1 | 730 | £26.59 |
| Statins | Monitoring | £1 | Add reference | 1 | 2 | £2.00 |
|  |  |  |  |  |  | £28.59 |

## Anti-hypertensives

A search of the literature did not identify any recent publications of anti-hypertensive prescriptions in the UK. As a consequence the best estimates of cost of anti-hypertensive treatment dated from 2004 (48). These were inflated to current prices. Due to the number of different anti-hypertensive treatments available and possibilities for combination therapies, using the cost from this study of prescriptions was preferred to using costs directly from the BNF.

Table 45: Annual cost of anti-hypertensive prescription expenditure per patient

|  | **Price** | **Inflation** | **Cost per year** | **Source** |
| --- | --- | --- | --- | --- |
| Anti-hypertensive prescriptions | £144 | 1.322 | £195.94 | (48) |

Recent guidelines for hypertension have recommended that hypertension be confirmed with ambulatory blood pressure monitoring (ABPM) (15). The cost of ABPM assessment is included in the cost of diagnosis (£53.40) (19), however, we assume that the test does not alter the initial diagnosis.

## Cardiovascular Events

Costs for coronary heart disease were obtained from a 2009 HTA for high dose lipid-lowering therapy (13). The costs of stroke were obtained from a study estimating costs from the Oxford vascular cohort (49). Table 46 describes the costs and resource use assumptions that were used for this study. It also reports the health states to which we have applied each cost in the model. The costs of congestive heart failure were estimated from the UKPDS costing study for complications related to diabetes (50). The unit costs for cardiovascular events are detailed in Table 47.

Table 46: Resources use assumptions and costs for cardiovascular outcomes

|  | Resource assumptions | Cost (2009) | Cost (2014/15) | Health States applied |
| --- | --- | --- | --- | --- |
| Unstable Angina year 1 | Secondary care costs: 100% hospitalisation, 50% revascularisation procedure, three outpatient appointments).  Primary care costs (three GP visits) and medications | £3880 | £4,674 | UANG1 |
| MI year 1 | Secondary care costs: 100% hospitalisation,  50% revascularisation procedure, three outpatient appointments)  Primary care costs (three GP visits) and medications. | £3996 | £4,813 | MI1 |
| Subsequent ACS care costs | Secondary care costs (one outpatient appointment).  Primary care costs (three GP visits) and medications. | £340 | £410 | SANG, UANG, MI |
| Stroke year 1 | Costs of first year post stroke (49) | £10,524 | £12,677 | STRO1 |
| Stroke subsequent costs | Average costs in years 2-5 following stroke (49) | £1,444 | £1,740 | STRO2 |
| Transient Ischemic Attack | Hospital costs from 5 year study | £2,260 | £2,723 | TIA |
| Fatal CHD | Palmer et al. (51). Assumed that 50% of fatalities incurred cost. | £592 | £665.50 |  |
| Fatal non cardiac vascular event | Youman et al. (52). Assumed 50% fatalities incurred cost | £3688 | £4149.52 |  |
|  | Source | Cost (2012) | Cost (2014/15) |  |
| Congestive heart failure year 1 | UKPDS (50) | £3,191 | £3,091 |  |
| Congestive Heart Failure subsequent years | UKPDS (50) | £1,473 | £1,818 |  |

Table 47: Unit costs for Cardiovascular cost estimates taken from HTA report (13)

| **Unit Cost** | **Mean** | **Inflation** | **Mean (2014/15)** | **Standard error** | **Distribution** |
| --- | --- | --- | --- | --- | --- |
| Unstable Angina hospital: EB05SZ | £1059 | 1.2045 | £1275 | 120.5447 | GAMMA |
| Revasc. Hospital mixture of HRG codes | £5011.81 | 1.2045 | £6037 | 570.4883 | GAMMA |
| MI Hospital: EB107 | £1290.88 | 1.2045 | £1555 | 146.9393 | GAMMA |
| First Outpatient | £137.28 | 1.2045 | £165 | 15.62642 | GAMMA |
| Subsequent appointment | £91.37 | 1.2045 | £110 | 10.40054 | GAMMA |
| GP visit year1 | £102 | 1.2045 | £123 |  | CONSTANT |
| GP visit year 2 | £91.37 | 1.2045 | £110 |  | CONSTANT |
| Fatal CHD (Palmer (51) Inflated) | £591.52 | 1.2045 | £713 | 67.332 | GAMMA |
| Fatal stroke (Youman (52) inflated) | £3688.23 | 1.2045 | £4443 | 419.8267 | GAMMA |
| Glytrin Spray | £10.47 | 1.2045 | £12.61 |  | CONSTANT |
| Isosorbide mononitrate | £11.24 | 1.2045 | £13.54 |  | CONSTANT |
| Verapamil | £41.98 | 1.2045 | £50.57 |  | CONSTANT |
| Atenolol | £30.24 | 1.2045 | £36.42 |  | CONSTANT |
| Aspirin | £6.65 | 1.2045 | £8.01 |  | CONSTANT |
| Ramipril | £75.09 | 1.2045 | £90.45 |  | CONSTANT |
| ARB | £210.27 | 1.2045 | £253 |  | CONSTANT |
| Clopidogrel | £460.27 | 1.2045 | £554 |  | CONSTANT |

## Microvascular Events

### Renal Failure

The cost of renal failure was estimated for the UK using relevant published studies. A recent costing study reported the costs of dialysis types (53). The prevalence of dialysis and transplants were taken from a second study reporting the prevalence of renal failure in the UK in 2008 (54). The cost of renal transplantation was taken from a costing study investigating the cost-effectiveness of renal transplantation (55). The overall cost was estimated as a weighted average of the treatment outcomes. All costs were inflated to 2012/13 prices.

Table 48: Unit costs for renal failure

|  | **Cost (£)** | **Source** | **Inflation** | **Cost (2014/15)** | **Proportion** |
| --- | --- | --- | --- | --- | --- |
| Haemodialysis with overheads | 34,236 | (53) | 1.2282 | £42,049 | 0.469 |
| Automated peritoneal dialysis (APD) | 22,160 | (53) | 1.2282 | £27,217 | 0.045* |
| Continuous ambulatory peritoneal dialysis (CAPD) | 16,074 | (53) | 1.2282 | £19742 | 0.045* |
| Transplant | 17,000 | (55) | 1.3918 | £23,660 | 0.442 |
| Immunosuppressant | 5000 | (55) | 1.3918 | £6,959 |  |
| * Assumed 50% split of peritoneal dialysis types | | | | | |

### Foot Ulcers

A search of the literature did not identify any studies for foot ulcer for the UK or a health system comparable to the UK. The cost of foot ulcers was estimated from a US Cost of Illness study (56). We acknowledge that this is a limitation of the analysis, because US costs may not be representative of care in the UK. The costs were converted from dollars to pounds using Purchasing Power Parities reported by the OECD (57). The costs were also inflated to UK 2012/13 prices.

Table 49: Estimated cost of foot ulcers

| **Resource component** | **Not Infected** | **With Cellulitis** | **With Osteomyelitis** |
| --- | --- | --- | --- |
| Prevalence | 0.874 | 0.09 | 0.036 |
| Mean cost per patient | $178.97 | $472.73 | $876.52 |
| Mean cost per patient (2012/13 £) | £158.53 | £418.73 | £776.40 |
| Standard error | 15.85 | 41.87 | 77.64 |
| Total Cost PPP (2012/13 £) | | | £204.19 |

### Amputation

The cost of amputation in the first year of surgery and subsequent years has been reported In a UKPDS costing study (50). The costs were extracted and inflated to 2014/15 prices. The cost of amputation in the first year was £10,101 (standard error £2,295) and in subsequent years was £1,896 (standard error £884).

### Blindness

The cost of blindness in the first year of surgery and subsequent years has been reported In a UKPDS costing study (50). The costs were extracted and inflated to 2014/15 prices. The cost of blindness in the first year was £1,433 (standard error £448) and in subsequent years was £479 (standard error £143).

## Cancer

The cost of breast and colorectal cancer is estimated as a one-off fixed cost at diagnosis in the model. This simplifying assumption means that the cost of cancer treatment is independent of survival. We acknowlegde that this assumption will affect the timing of costs because all costs are imposed in the first year and subject to less discounting. However, we anticipate that the impact on overall outcomes will not be substantial. A large proportion of costs are will be incurred in the first year of treatment (surgery, chemotherapy, radiotherapy). Costs in subsequent years will be lower for patients who achieve remission and survival will be short in patients who relapse. Therefore, the costs are likely to be skewed to the early years post diagnosis.

A recent appraisal for cancer screening estimated the overall cost of breast cancer as a weighted average depending on the prognosis at diagnosis to be £10,452 in 2006/7 prices and £13,818 when inflated to 2014/15 prices (58).

The cost of colorectal cancer was taken from a screening appraisal which reported the lifetime costs of colorectal cancer according to the Dukes stage of the tumour (59). The appraisal also reported the proportion of cancers identified at each stage, which allowed us to estimate the weighted average cost of colorectal cancer. Table 50 reports the overall cost of colorectal cancer by stage of disease at diagnosis.

Table 50: Estimated cost of colorectal cancer

| Resource component | Dukes’ Stage A | Dukes’ Stage B | Dukes’ Stage C | Stage D |
| --- | --- | --- | --- | --- |
| Number of patients | 3241.92 | 9,431.04 | 7,662.72 | 8,841.60 |
| Prevalence | 0.111 | 0.323 | 0.263 | 0.303 |
| Mean cost per patient | £7,250.84 | £12,441.41 | £19,076.90 | £11,945.78 |
| Price Inflation | | | | 1.392 |
| Mean cost per patient (2014/15) | £10,091 | £17,315 | £26,550 | £16,626 |
| Standard error (2014/15) | £953.64 | £1,732 | £2,655 | £1,663 |
| Total Cost (2014/15) | | | | £18,729 |

## Osteoarthritis

The annual cost of osteoarthritis were estimated in a report in 2010 (60). In this report the authors estimated the expected cost of osteoarthritis from three previous costing studies. The costs include GP attendance, nurse consultations, replacement surgery, help at home and prescription medications. The estimated annual cost of osteoarthritis was £783 in £2008. This was inflated to 2014/15 prices at £962 (standard error £96).

## Depression

Depression is modelled as a chronically recurrent disorder, with patients experiencing further depressive episodes after remission. In the model it is assumed that patients continue to incur costs of depression following an initial diagnosis. These costs reflect ongoing resource use to deal with relapse and prevention of relapse.

A recent trial to prevent secondary depressive episodes collected comprehensive cost data from a sample of individuals with depression (61). The cost estimate reflects the ongoing cost of depression after an acute episode, therefore is compatible with our characterisation of depression as a chronic condition. The resource uses identified in the control arm were extracted to estimate the costs of depression. The costs from this data (inflated to 2014/15 prices) were not implemented directly into the SPHR diabetes prevention model as this would have over-estimated the number of GP visits. The model already accounts for GP attendance due to depression as described in Section 10.1. Therefore, a revised estimate of the cost of depression, excluding GP consultation was estimated using updated unit costs. The resource use estimates and revised unit cost estimates used to generate a cost of depression excluding GP utilisation are reported in Table 51.

Table 51: Depression utilisation of services and total estimated cost

|  | Assumption for costs | Unit cost | Source | Inflation | Annual utilisation | Source | Cost per year |
| --- | --- | --- | --- | --- | --- | --- | --- |
| Practice nurse at surgery | GP nurse face to face assume 10 mins | £8.83 | (62) | 1.0206 | 1.52 | (61) | £13.70 |
| Practice nurse at home visit | GP nurse face to face assume 30 mins | £26.50 | (62) | 1.0206 | 0.02 | (61) | £0.54 |
| Practice nurse telephone | GP nurse face to face assume 10 mins | £8.83 | (62) | 1.0206 | 0.11 | (61) | £0.99 |
| Health visitor | Health visitor per hour visit 30 mins | £35.50 | (62) | 1.0206 | 0.05 | (61) | £1.94 |
| District nurse | Community nurse 30 mins | £24.50 | (62) | 1.0206 | 0.01 | (61) | £0.38 |
| Other nurse | GP nurse face to face assume 10 mins | £8.83 | (62) | 1.0206 | 0.13 | (61) | £1.17 |
| HCA phelbotomist | Clinical support worker 10 mins | £4.17 | (62) | 1.0206 | 0.31 | (61) | £1.05 |
| Other primary care | Advanced nurse with qualifications | £25.00 | (62) | 1.0206 | 0.19 | (61)) | £4.85 |
| Out of hours | Inflated of trial costs | £22.30 | (62) | 1.2045 | 0.23 | (61) | £6.18 |
| NHS direct | Inflated of trial costs | £21.00 | (62) | 1.2045 | 0.09 | (61) | £2.28 |
| Walk-in centre | Inflated of trial costs | £32.24 | (62) | 1.2045 | 0.21 | (61)) | £8.15 |
| Prescribed medications | Inflated of trial costs | £7.98 | (62) | 1.2045 | 7.74 | (61) | £74.42 |
| Secondary care | Emergency Medicine, Any Investigation | £109.0 | (42) | 1 | 0.26 | (61) | £21.06 |
|  | | | | | | | £136.71 |

# Employer COsts

In order to capture wider social benefits of interventions for diabetes prevention, the model was designed to estimate the number of sickness days taken conditional on health status. The model utilises data from a study that estimates productivity loss due to poor health, using days absent from paid employment and normal activities, EQ-5D score, International Classification of Disease (ICD) chapter and socio-demographic data (63). The results can be used to predict the level of productivity loss associated with EQ-5D values and specific disease diagnosis, measured by number of days absent from work. Data was used from a prospective survey of inpatients discharged from a hospital in Wales, United Kingdom from April 2002 to January 2009. The number of days absent from paid employment due to ill health (N=51,326) in the six weeks following discharge was estimated using a zero-inflated negative binomial regression model, which produced large spikes at 0 (zero days off paid employment/normal activities) and 42 days.

The following disease diagnoses were used in the model to estimate the impact of disease on work productivity.

1. Colorectal or breast cancer were associated with ICD group for neoplasms.
2. Diabetes diagnosis was associated with ICD group for Endocrine, nutritional and metabolic diseases.
3. Depression was associated with ICD group for Mental and behavioural disorders.
4. Cardiovascular disease was associated with ICD group for diseases of the circulatory system.

The statistical model estimated the number of days absent from work from an employed population. In the SPHR model the number of days absent from work was only applied to individuals in the HSE who reported being in employment and less than 65 years old. The simulated number of days of sick leave was multiplied by 8.67 to scale up the 6 week estimate to the annual cycle of the model.

The cost of sick days to the employer was calculated based on a method derived from a previous study of work absenteeism (64). In the SPHR model it is assumed that the employee’s usual salary is not included in the employer cost because the productivity of the replacement worker would generate gains to the employer to compensate for the absent worker. Therefore the employer cost calculation includes excess costs incurred and/or loss of productivity during periods of worker absence. The cost to the employer of work absence due to ill health is based on the number of days of absence, losses due to work not completed, occupational sick pay and the cost of a replacement worker. Productivity losses were estimated based on the friction method, which assumes that there are sufficient number of unemployed people within the UK in order to replace workers on sick leave after a given friction period. In this analysis we assumed a friction period of 10 weeks during which the employer incurs a cost due to productivity losses. After the friction period a replacement worker is assumed to be as productive as the employee on sick leave. During the sick leave period there are costs incurred because the employer is obliged to pay statutory sick pay for 28 weeks and many employers also provide occupational sick pay (OSP). In this analysis we assumed that the employee receives full pay for 15 weeks. Within the friction period this payment is subsumed into the employee’s usual salary, which would have been paid by the employer in the absence of sick leave. However, after the friction period the OSP is included in our estimate of the employer’s cost, because in this period the employer would be paying for the employee on sick leave in addition to a replacement worker. If the period of absence exceeds 15 weeks (75 days) the employer pays half the salary for a maximum of 16.4 weeks, and no further payments for the remaining period. Table 52 summarises the timing of costs incurred due to periods of absence from work.

The average salary per day is based on a UK national average salary plus national insurance contributions at this salary (65). The cost of a replacement worker was calculated in a recent report which estimated the logistical costs of advertising spend for a new employee, the cost of using an agency to recruit for a new employee and the number of days taken for internal HR processes related to a new employee (66). We assume that there are no additional costs to training the replacement worker.

Table 52: Employer cost algorithm for days absent from work due to ill health

| Days absence | Productivity lost over friction period | Occupational sick pay | Cost replacement worker |
| --- | --- | --- | --- |
| 1-50 | £103.4 per day |  |  |
| 51-75 |  | Full pay £103.4 per day | Cost of advertising and recruitment temporary worker £5433. |
| 76-157 |  | Half pay £51.7 per day |  |
| 157-260 |  | None |  |

If an individual of working age dies whilst in employment, the cost of recruiting a replacement worker is included in the calculation of employer costs.

# Utilities

## Baseline Utility

Baseline utilities for all individuals in the cohort were extracted from the HSE 2011. The tariffs for the responses to the 3 level EQ-5D were derived from a UK population study (67). Baseline utility was assumed to decline due to ageing. In the simulation, utility declines by an absolute decrement of 0.004 per year. This estimate is based on previous HTA modelling in cardiovascular disease (14).

## Utility Decrements

The utility decrements for long term chronic conditions were applied to the age and BMI adjusted EQ-5D score. We assumed that a diagnosis of diabetes was not associated with a reduction in EQ-5D independent of the utility decrements associated with complications, comorbidities or depression. Cardiovascular disease, renal failure, amputation, foot ulcers, blindness, cancer, osteoarthritis and depression were all assumed to result in utility decrements. The utility decrements are measured as a factor which is applied to the individual’s age and BMI adjusted baseline. If individuals have multiple chronic conditions the utility decrements are multiplied together to give the individual’s overall utility decrement from comorbidities and complications, in line with current NICE guidelines for combining comorbidities (68).

Due to the number of health states it was not practical to conduct a systematic review to identify utility decrements for all health states. A pragmatic approach was taken to search for health states within existing health technology assessments for the relevant disease area or by considering studies used in previous economic models for diabetes prevention. Discussions with experts in health economic modelling were also used to identify prominent sources of data for health state utilities.

Two sources of data were identified for diabetes related complications. A recent study from the UKPDS estimated the impact of changes in health states from a longitudinal cohort (69). They estimated the impact of myocardial infarction, ischaemic heart disease, stroke, heart failure, amputation and blindness on quality of life using seven rounds of EQ-5D questionnaires administered between 1997 and 2007. This data was used to estimate the utility decrement for amputation and congestive heart failure. The absolute decrement for amputation was converted into utility decrement factors that could be multiplied by the individuals’ current EQ-5D to estimate the relative effect of the complication. Blindness was included in the statistical model used for this analysis however the UKPDS analysis reported an increase in health state utility following a diagnosis with blindness. Discussions with the authors highlighted that this was due to treatment following formal classification with blindness and it was decided that this increase in health state utility should not be included in the cost-effectiveness model.

Utility decrements for renal failure and foot ulcers were not available from the UKPDS study described above. A study by Coffey et al. (2000) was used to estimate utility decrements for renal failure and foot ulcers (70). In this study, 2,048 subjects with type 1 and type 2 diabetes were recruited from specialty clinics. The Self-Administered Quality of Well Being index (QWB-SA) was used to calculate a health utility score.

A meta-analysis of utility values for diabetes and diabetes related complications estimated utility decrements for amputation, ulcer, end stage renal failure and blindness (71). The study pooled utility measures using different health state valuation measures in a meta-analysis. Pooling health state utility values is problematic because of the fact that different valuation methods and different preference-based measures (PBMs) can generate different values on exactly the same clinical health state (72).There were not sufficient studies in the meta-analysis to adjust for the effects of health state valuation measure on the result. This is a limitation of the analysis and we decided that it was preferable to use estimates from single studies.

Utility decrements for cardiovascular events were taken from an HTA assessing statins to reflect the utility decrements in all patients (14) rather than using the UKPDS, which is only representative of a diabetic population. The study conducted a literature review to identify appropriate utility multipliers for stable angina, unstable angina, myocardial infarction and stoke. We used these estimates in the model and assume that transient ischaemic attack is not associated with a utility decrement in line with this HTA.

We identified a systematic review of breast cancer utility studies following consultation with colleagues with experience in this area. The review highlighted a single burden of illness study with a broad utility decrement for cancer (73), rather than utilities by cancer type or disease status. This study was most compatible with the structure of the cost-effectiveness structure. Within this study 1823 cancer survivors and 5469 age-, sex-, and educational attainment-matched control subjects completed EQ-5D questionnaires to estimate utility with and without cancer.

The utility decrement for osteoarthritis was taken from a Health Technology Assessment that assessed the clinical effectiveness and cost-effectiveness of glucosamine sulphate/hydrochloride and chondroitin sulphate in modifying the progression of osteoarthritis of the knee (74).

A review of cost-effectiveness studies highlights the scarcity of studies of health-related quality of life in depression (75). The utility studies identified in the review described depression states by severity and did not adjust for comorbid conditions. Furthermore, the valuations were variable between studies suggesting poor consistency in the estimations. Therefore, it was difficult to apply these in the model. We decided to use a study which had used the EQ-5D in an RCT, for consistency with our utility measure (76). They report an average post treatment utility of 0.67, from which we estimated the utility decrement compared with the average utility reported in the HSE dataset. The decrement was then converted into a relative utility reduction.

Table 53 reports the multiplicative utility factors that are used in the model to describe health utility decrements from comorbid complications. The mean absolute decrement estimated in each study is reported alongside the baseline utility for each study. The utility factor was estimated by dividing the implied health utility with the comorbidity by the baseline utility.

**Table 53: Utility decrement factors**

|  | Mean Absolute decrement | St. error absolute decrement | Baseline Utility | Multiplicative Utility Factor | Source |
| --- | --- | --- | --- | --- | --- |
| Foot ulcer | -0.099 | 0.013 | 0.689 | 0.856 | Coffey (70) |
| Amputation | -0.172 | 0.045 | 0.807 | 0.787 | UKPDS (69) |
| Blind | 0.033 | 0.027 | 0.807 | 1.041 | UKPDS (69) |
| Renal failure | -0.078 | 0.026 | 0.689 | 0.887 | Coffey (70) |
| Stable Angina |  |  |  | 0.801 | Ward HTA (14) |
| Unstable Angina y1 |  |  |  | 0.770 | Ward HTA (14) |
| Unstable Angina y2 |  |  |  | 0.770 | Ward HTA (14) |
| Myocardial Infarction y1 |  |  |  | 0.760 | Ward HTA (14) |
| Myocardial Infarction y2 |  |  |  | 0.760 | Ward HTA (14) |
| Transient Ischaemic Attack |  |  |  | 1.000 | Ward HTA (14) |
| Stroke y1 |  |  |  | 0.629 | Ward HTA (14) |
| Stroke y2 |  |  |  | 0.629 | Ward HTA (14) |
| Breast Cancer | -0.060 |  | 0.800 | 0.913 | Yabroff (73) |
| Colorectal Cancer | -0.060 |  | 0.800 | 0.913 | Yabroff (73) |
| Osteoarthritis | -0.101 |  |  |  | Black HTA (74) |
| Depression | -0.116 |  | 0.7905 | 0.875 | Benedict (76) |
| Congestive Heart Failure | -0.101 | 0.032 |  | 0.875 | UKPDS (69) |
| UKPDS baseline utility 0.807; HSE baseline 0.7905 | | | | | |

# Model Validation

The SPHR model has undergone a thorough process of error checking and internal and external validations. Model verification (comprising error checking and internal validation) included ensuring that mean PSA sampling values corresponded to mean parameter values, following individuals over time as they went through the model to ensure that trajectories were behaving as expected in response to treatments and interventions, and building the QRISK2 (22) separately in Excel to ensure that CVD predictions were accurate. We also tested the ability of the model to predict the results of the UKPDS outcomes model v2 (20), which acted as an internal validation for those outcomes encoded by UKPDS in the SPHR model, and an external validation for cardiovascular outcomes that were encoded in the SPHR model using QRISK2 (see below). As a consequence of this we realised that the SPHR model was vastly overestimating the incidence of renal failure, and resulted in our switching to UKPDS v1 for this outcome (12).

We developed four tests to compare model outcomes with reported data from external data sources. The first test assessed the incidence of type-2 diabetes in the population and sub-groups of the population and compared the results with incidence data from the EPIC study. This tested whether the Whitehall II glycaemia trajectories were performing adequately. The second validation study simulated data from the HSE 2003 cohort for eight years to observe predicted distributions of metabolic risk factors and the prevalence of health outcomes and compared them with the HSE 2011 cohort. The third validation exercise simulated the ADDITION diabetes trial and observed whether similar outcomes were observed compared with the data. Finally, a diabetic cohort was simulated in the model to compare outcomes with the UKPDS data.

## Prediction of Diabetes Incidence

This validation assessed whether the Whitehall II model for glycaemia trajectories predicted incidence of diabetes diagnosed by the HbA1c test in sub-groups of patients from the HSE2003 cohort. Its objective was to assess the ability of the Whitehall II trajectories in the model to predict the recorded incidence of diabetes.

### Methods

Data summarising the incidence of type 2 diabetes was obtained from the EPIC Norfolk cohort (77), which of similar studies was thought to be the most likely to represent the population distribution found in the HSE. EPIC Norfolk consists of 5735 individuals aged between 40 and 74, without diabetes at baseline, who were followed for three years after an initial HbA1c test in the late 1990s. Diabetes incidence rates included both doctor diagnosed diabetes and diagnosis due to HbA1c≥48 mmol/mol (6.5%) in the follow-up period. Diabetes incidence was recorded for the total population and for sub-groups based upon their initial HbA1c status.

The model was run over a three year time course sampling only those individuals aged between 40 and 74 at the start of simulation. The starting population was generated from HSE 2003 rather than HSE 2011, as the dataset was more contemporaneous with the EPIC study and better reflected the population distribution within the IGR categories. Individuals with diabetes were excluded from the starting population. Diabetes incidence after three years of simulation was determined for subgroups of individuals who were initially measured with a high level of impaired glucose regulation (IGR) (HbA1c = 6.0-6.4%) or moderate IGR (HbA1c = 5.5-5.9%), and for the total population. Diabetes incidence in the model was represented by the total percentage of individuals in which HbA1c was measured as ≥48 mmol/mol (6.5%) in two consecutive health checks, rather than those with a diagnosis of diabetes, in order to reflect the EPIC data.

### Results & Discussion

The incidence of diabetes in the simulation is summarised in Table 54. The diabetes model overestimates diabetes incidence in both the total population and in the high IGR population, but underestimates diabetes incidence in the moderate IGR population. The overestimation of total population incidence is likely to be a consequence of the overestimation of diabetes incidence in the high IGR group.

Baseline populations are relatively similar and cannot account for the large differences in diabetes incidence in the high and moderate IGR subgroups. We investigated whether the differences in incidence between high and moderate groups are due to differences between the Whitehall II cohort and the EPIC data. HbA1c values were collected in phases 7 and 9 of the Whitehall II cohort. The incidence of diabetes (defined by HbA1c greater than 48 mmol/mol (6.5%)) after 5 years observation in the cohort was 2.5%. The incidence among high IGR was 44.2% and among moderate IGR was 5.12%. Therefore, the Whitehall II cohort does observe a much greater incidence of diabetes among those individuals with high IGR than the EPIC cohort. However, the low incidence of diabetes among those individuals with moderate IGR is not reflected in the Whitehall II cohort data.

Table 54: Comparison of simulated outcomes with the EPIC-Norfolk cohort

|  | **Diabetes Prevention Model (N=50000)** | | **EPIC-Norfolk (N=5735)** | | | |
| --- | --- | --- | --- | --- | --- | --- |
| Baseline characteristics | | | | | | |
|  | Mean | Standard deviation | Mean | | Standard deviation | |
| **Age (years)** | 55.1 | 9.8 | 57.4 | | 9.4 | |
| **Male (%)** | 45.2 | N/A | 43.3 | | N/A | |
| **BMI (kg/m^2^)** | 27.7 | 5.0 | 25.9 | | 3.7 | |
| Diabetes incidence after 3 years | | | | | | |
| Subset of individuals | Percentage of total individuals | Mean diabetes incidence (%)* | Percentage of total individuals | Mean diabetes incidence (%)** | | 95% CI |
| **High IGR**  **(HbA1c = 6.0-6.4)** | 5.9 | 11.2 | 6.5 | 7.0 | | 4.8-10.1 |
| **Moderate IGR (HbA1c = 5.5-5.9)** | 27.9 | 0.2 | 24.4 | 1.5 | | 1.0-2.3 |
| **Total Population** | 100 | 2.2 | 100 | 1.3 | | 1.0-1.5 |
| BMI Body Mass Index; CI Confidence Interval  *HbA1c≥48 mmol/mol (6.5%) in two consecutive health checks; **HbA1c≥48 mmol/mol (6.5%) and/or doctor diagnosis of diabetes | | | | | | |

The low diabetes incidence among individuals with moderate IGR is likely to be a consequence of the way the trajectories for HbA1c work in the model. Most individuals have a gradually increasing HbA1c as they age, meaning that three years is insufficient in the vast majority of cases for a someone with moderate IGR to progress to diabetes. Equally, many more people with high IGR will progress to diabetes as they are already close to the threshold. We suggest that the model may be more accurate at predicting diabetes incidence over a longer time period due to the nature of the quadratic equations used to predict HbA1c.

Finally, it is possible that some individuals within the EPIC study who learnt they had high IGR at the beginning of the study would have changed their behaviour as a consequence of their high risk of diabetes or as a consequence of undergoing health screening (although this was not reported). Changes to diet and exercise would impact upon HbA1c and lead to underestimates of diabetes incidence. No such effect would occur in the model as individuals are unaware of their HbA1c status.

## Using Data from HSE 2003 to predict HSE 2011

This validation aimed to observe whether the Whitehall II statistical model predicted the future distribution of metabolic risk factors and prevalence of diabetes in age-selected sub-groups from the HSE 2003 data. We were aware that the Whitehall II cohort does not necessarily describe prospective changes in the population metabolic risk factors that were forecast. This analysis identified the potential error in the Whitehall II statistical models to describe temporal changes in the risk profile of the population. The analysis also monitored whether the prevalence of diabetes and cardiovascular disease were correctly estimated for the age-groups. The objective was to evaluate the ability of the model to reproduce observed changes in population wide metabolic data between two time points.

### Methods

Data from HSE 2003 was chosen as a starting point as values were obtainable for most of the parameters used in HSE 2011 (78). The total sample size of HSE 2003 is 18,553, which is considerably larger than the sample size of HSE 2011 (10,617).

Data was extracted from HSE 2003 using similar methods to those used for extraction of data from HSE 2011. A value for one of the parameters used in HSE 2011 was unavailable. This referred to diagnosis of “diabetes from blood sample or doctor diagnosis”. However, the question on “doctor diagnosed diabetes” was thought to be adequate to assign individuals a diagnosis of diabetes. Individuals with a prior diagnosis of diabetes were included for the purposes of validation, but individuals under the age of 16 (n=3717) were removed from the dataset, resulting in a final sample size of 14,836. Missing data was estimated in the same way as described in section 6.4.

As for HSE 2011, QRISK scores and EQ-5D scores were calculated for all individuals. To align ethnicity in HSE 2003 to QRISK, all ‘Asian’ and ‘British Asian’ individuals were assumed to be ‘Indian’ (largest Asian subgroup and median risk ratio within all Asian subgroups in QRISK), all ‘Black’ and ‘British Black’ individuals were assumed to be ‘Black Caribbean’ (largest Black subgroup) and ‘Mixed Race’ individuals were assumed to be ‘Other’. For those with a history of cardiovascular disease, the nature of the illness was randomly assigned according to age and sex-related probabilities of different types of cardiovascular event. The characteristics of HSE 2003 are described in Table 55.

Table 55: Characteristics of the final sample from HSE 2003 (N=14836), including individuals with diagnosed diabetes

|  | Number | Percentage |  |
| --- | --- | --- | --- |
| **Male** | 6602 | 44.50 |  |
| **White** | 13661 | 92.08 |  |
| **IMD1 (least deprived)** | 3334 | 22.47 |  |
| **IMD2** | 2950 | 19.88 |  |
| **IMD3** | 2929 | 19.74 |  |
| **IMD4** | 3059 | 20.62 |  |
| **IMD5 (most deprived)** | 2564 | 17.28 |  |
| **Non-smoker** | 7445 | 50.18 |  |
| **Anti-hypertensive treatment** | 2178 | 14.68 |  |
| **Statins** | 791 | 5.33 |  |
| **Diagnosed Diabetes** | 611 | 4.12 |  |
| **Cardiovascular Disease (CVD)** | 1119 | 7.54 |  |
| SUBTYPES OF CVD | Number | Percentage of CVD |  |
| **Stable Angina** | 411 | 36.73 |  |
| **Unstable Angina** | 122 | 10.90 |  |
| **MI** | 246 | 21.98 |  |
| **TIA** | 69 | 6.17 |  |
| **Stroke** | 271 | 24.22 |  |
|  | Mean | Standard Deviation | Median |
| **Age (years)** | 48.21 | 18.49 | 47.00 |
| **BMI (kg/m^2^)** | 26.96 | 5.01 | 26.35 |
| **Total Cholesterol (mmol/L)** | 5.70 | 1.18 | 5.60 |
| **HDL Cholesterol (mmol/L)** | 1.53 | 0.39 | 1.50 |
| **HbA1c (%)** | 5.34 | 0.73 | 5.20 |
| **Systolic Blood Pressure (mm Hg)** | 129.30 | 19.13 | 126.50 |
| **EQ-5D (TTO)** | 0.862 | 0.223 | 1.000 |
| BMI Body Mass Index; IMD Index of Multiple Deprivation; EQ-5D 5 dimensions Euroqol (health related quality of life index); MI Myocardial Infarction; TIA Transient Ischaemic Attack | | | |

For the purposes of validation, it was also necessary to include the characteristics of patients with diagnosed diabetes in the HSE 2011 sample as a comparison with the projected HSE 2003 data. The characteristics of HSE 2011 including diagnosed diabetics are summarised in Table 56.

Table 56: Characteristics of the final sample from HSE 2011 (N=8610), including individuals with diagnosed diabetes

|  | Number | Percentage |  |
| --- | --- | --- | --- |
| **Male** | 3822 | 44.39 |  |
| **White** | 7719 | 89.65 |  |
| **IMD1 (least deprived)** | 1774 | 20.60 |  |
| **IMD2** | 1823 | 21.17 |  |
| **IMD3** | 1830 | 21.25 |  |
| **IMD4** | 1597 | 18.55 |  |
| **IMD5 (most deprived)** | 1586 | 18.42 |  |
| **Non-smoker** | 4550 | 52.85 |  |
| **Anti-hypertensive treatment** | 1544 | 17.93 |  |
| **Statins** | 929 | 10.79 |  |
| **Diagnosed Diabetes** | 572 | 6.64 |  |
| **Cardiovascular Disease (CVD)** | 639 | 7.42 |  |
| SUBTYPES OF CVD | Number | Percentage of CVD |  |
| **Stable Angina** | 232 | 36.31 |  |
| **Unstable Angina** | 83 | 12.99 |  |
| **MI** | 137 | 21.44 |  |
| **TIA** | 40 | 6.26 |  |
| **Stroke** | 147 | 23.00 |  |
|  | Mean | Standard Deviation | Median |
| **Age (years)** | 49.64 | 18.70 | 49.00 |
| **BMI (kg/m^2^)** | 27.39 | 5.36 | 26.64 |
| **Total Cholesterol (mmol/l)** | 5.42 | 1.07 | 5.40 |
| **HDL Cholesterol (mmol/l)** | 1.52 | 0.44 | 1.50 |
| **HbA1c (%)** | 5.73 | 0.78 | 5.60 |
| **Systolic Blood Pressure (mm Hg)** | 126.50 | 17.00 | 124.50 |
| **EQ-5D (TTO)** | 0.825 | 0.244 | 0.848 |
| BMI Body Mass Index; IMD Index of Multiple Deprivation; EQ-5D 5 dimensions Euroqol (health related quality of life index); MI Myocardial Infarction; TIA Transient Ischaemic Attack | | | |

Individuals from HSE 2003 were grouped into five different age bands (A=20-29, B=30-39, C=40-49, D=50-59, E=60-69), which were simulated separately. 50,000 individuals were generated for each age group then the model was run over a time course of 8 years to simulate the aging of individuals between 2003 and 2011. For each age group, separate sets of distribution statistics were obtained for HbA1c, BMI, systolic blood pressure, total and HDL cholesterol, diabetes prevalence and cardiovascular disease prevalence before and after simulation. This was compared with data extracted from equivalent age bands for HSE 2011 (A’=28-37, B’=38-47, C’=48-57, D’=58-67, E’=68-77).

### Results & Discussion

Distribution statistics for each age group are presented in Table 57-Table 61. The differences between the HSE 2003 modelled projection and the HSE 2011 data are summarised as follows:

- There is a general tendency for the model to slightly under-predict mean HbA1c (Figure 12).
- There is a general over-prediction of systolic blood pressure in the model in all age groups (
- Figure 13).
- BMI is over-predicted in the model, particularly in younger age groups (
- Figure 14).
- The model slightly over-predicts cholesterol levels in the youngest age group, but under-predicts it for the older groups (Figure 15).
- EQ-5D is slightly over-predicted in the model (
- Figure 16).
- The model over-predicts diabetes diagnoses in all age groups apart from the middle one (age 48-47), where the HSE 2011 has an unexpected peak in diabetes diagnoses (Figure 17). The over-prediction is most evident in the youngest age groups.
- The model predicts cardiovascular disease quite accurately, although slightly over-predicts in the oldest age groups (
- Figure 18).

Table 57: Comparison of simulated outcomes from HSE 2003 with actual data from HSE 2011: Age group A - 20-29

| **A** | **HSE 2003: Before simulation (n=1855)** | | | | **HSE 2003: After simulation (n=49607)** | | | | **HSE 2011:**  **(n=1339)** | | | |
| --- | --- | --- | --- | --- | --- | --- | --- | --- | --- | --- | --- | --- |
|  | **Age 20-29** | | | | **Age 28-37** | | | | **Age 28-37** | | | |
|  | Mean | SD | | Median | Mean | SD | | Median | Mean | SD | | Median |
| **Age (years)** | 24.8 | 2.9 | | 25.0 | 32.8 | 2.8 | | 33.0 | 32.5 | 2.9 | | 33.0 |
| **HbA1c (%)** | 5.0 | 0.4 | | 5.0 | 5.2 | 1.0 | | 5.2 | 5.4 | 0.6 | | 5.4 |
| **BMI kg/m^2^** | 25.1 | 5.0 | | 24.2 | 27.7 | 5.5 | | 26.8 | 26.8 | 5.4 | | 25.7 |
| **Systolic Blood Pressure (mm Hg)** | 119.0 | 11.9 | | 119.0 | 125.2 | 15.6 | | 125.3 | 118.2 | 12.6 | | 117.5 |
| **Total Cholesterol (mmol/l)** | 4.9 | 1.0 | | 4.8 | 5.3 | 0.9 | | 5.3 | 5.0 | 1.0 | | 4.9 |
| **HDL Cholesterol (mmol/l)** | 1.5 | 0.4 | | 1.5 | 1.5 | 0.4 | | 1.5 | 1.5 | 0.4 | | 1.5 |
| **EQ-5D** | 0.929 | 0.153 | | 1.000 | 0.901 | 0.212 | | 1.000 | 0.894 | 0.186 | | 1.000 |
|  | Number | | Percentage | | Number | | Percentage | | Number | | Percentage | |
| **Diabetes** | 14 | | 0.8 | | 1448 | | 2.9 | | 16 | | 1.2 | |
| **Cardiovascular Disease** | 7 | | 0.4 | | 343 | | 0.7 | | 6 | | 0.4 | |
| BMI Body Mass Index; SD Standard Deviation | | | | | | | | | | | | |

Table 58: Comparison of simulated outcomes from HSE 2003 with actual data from HSE 2011: Age group B - 30-39

| **B** | **HSE 2003: Before simulation (n=2788)** | | | | **HSE 2003: After simulation (n=49382)** | | | | **HSE 2011:**  **(n=1595)** | | | |
| --- | --- | --- | --- | --- | --- | --- | --- | --- | --- | --- | --- | --- |
|  | **Age 30-39** | | | | **Age 38-47** | | | | **Age 38-47** | | | |
|  | Mean | SD | | Median | Mean | SD | | Median | Mean | SD | | Median |
| **Age (years)** | 34.7 | 2.9 | | 35.0 | 42.7 | 2.9 | | 43.0 | 42.5 | 2.9 | | 42.0 |
| **HbA1c (%)** | 5.1 | 0.6 | | 5.1 | 5.4 | 1.0 | | 5.3 | 5.6 | 0.7 | | 5.5 |
| **BMI kg/m^2^** | 26.8 | 5.2 | | 26.0 | 29.0 | 5.7 | | 28.2 | 27.5 | 5.2 | | 26.6 |
| **Systolic Blood Pressure (mm Hg)** | 120.0 | 12.6 | | 119.0 | 127.4 | 15.1 | | 126.9 | 121.0 | 13.9 | | 120.0 |
| **Total Cholesterol (mmol/l)** | 5.4 | 1.1 | | 5.3 | 5.4 | 0.9 | | 5.4 | 5.4 | 0.9 | | 5.3 |
| **HDL Cholesterol (mmol/l)** | 1.5 | 0.4 | | 1.4 | 1.6 | 0.4 | | 1.5 | 1.5 | 0.4 | | 1.4 |
| **EQ-5D** | 0.911 | 0.178 | | 1.000 | 0.887 | 0.223 | | 1.000 | 0.849 | 0.226 | | 1.000 |
|  | Number | | Percentage | | Number | | Percentage | | Number | | Percentage | |
| **Diabetes** | 37 | | 1.3 | | 2245 | | 4.5 | | 50 | | 3.1 | |
| **Cardiovascular Disease** | 13 | | 0.5 | | 777 | | 1.6 | | 24 | | 1.5 | |
| BMI Body Mass Index; SD Standard Deviation | | | | | | | | | | | | |

Table 59: Comparison of simulated outcomes from HSE 2003 with actual data from HSE 2011: Age group C - 40-49

| **C** | **HSE 2003: Before simulation (n=2581)** | | | | **HSE 2003: After simulation (n=48882)** | | | | **HSE 2011:**  **(n=1412)** | | | |
| --- | --- | --- | --- | --- | --- | --- | --- | --- | --- | --- | --- | --- |
|  | **Age 40-49** | | | | **Age 48-57** | | | | **Age 48-57** | | | |
|  | Mean | SD | | Median | Mean | SD | | Median | Mean | SD | | Median |
| **Age (years)** | 44.2 | 2.9 | | 44.0 | 52.2 | 2.9 | | 52.0 | 52.3 | 2.9 | | 52.0 |
| **HbA1c (%)** | 5.3 | 0.8 | | 5.2 | 5.6 | 1.1 | | 5.4 | 5.8 | 0.9 | | 5.6 |
| **BMI kg/m^2^** | 27.4 | 5.2 | | 26.7 | 29.1 | 5.7 | | 28.4 | 28.4 | 5.5 | | 27.5 |
| **Systolic Blood Pressure (mm Hg)** | 124.9 | 16.0 | | 123.0 | 131.4 | 15.9 | | 131.0 | 127.6 | 15.9 | | 125.5 |
| **Total Cholesterol (mmol/l)** | 5.7 | 1.1 | | 5.6 | 5.5 | 0.9 | | 5.4 | 5.7 | 1.0 | | 5.7 |
| **HDL Cholesterol (mmol/l)** | 1.5 | 0.4 | | 1.5 | 1.6 | 0.4 | | 1.6 | 1.6 | 0.5 | | 1.5 |
| **EQ-5D** | 0.882 | 0.204 | | 1.000 | 0.857 | 0.246 | | 1.000 | 0.807 | 0.261 | | 0.848 |
|  | Number | | Percentage | | Number | | Percentage | | Number | | Percentage | |
| **Diabetes** | 57 | | 2.2 | | 3286 | | 6.7 | | 108 | | 7.6 | |
| **Cardiovascular Disease** | 46 | | 1.8 | | 2183 | | 4.5 | | 64 | | 4.5 | |
| BMI Body Mass Index; SD Standard Deviation | | | | | | | | | | | | |

Table 60: Comparison of simulated outcomes from HSE 2003 with actual data from HSE 2011: Age group D - 50-59

| **D** | **HSE 2003: Before simulation (n=2564)** | | | | **HSE 2003: After simulation (n=47187)** | | | | **HSE 2011:**  **(n=1387)** | | | |
| --- | --- | --- | --- | --- | --- | --- | --- | --- | --- | --- | --- | --- |
|  | **Age 50-59** | | | | **Age 58-67** | | | | **Age 58-67** | | | |
|  | Mean | SD | | Median | Mean | SD | | Median | Mean | SD | | Median |
| **Age (years)** | 54.6 | 2.8 | | 55.0 | 62.6 | 2.8 | | 63.0 | 62.6 | 2.8 | | 63.0 |
| **HbA1c (%)** | 5.4 | 0.7 | | 5.3 | 5.7 | 1.1 | | 5.6 | 5.9 | 0.8 | | 5.8 |
| **BMI kg/m^2^** | 27.9 | 4.9 | | 27.2 | 29.1 | 5.4 | | 28.4 | 28.3 | 5.2 | | 27.6 |
| **Systolic Blood Pressure (mm Hg)** | 131.5 | 17.0 | | 130.0 | 136.1 | 15.6 | | 136.3 | 132.9 | 17.5 | | 131.5 |
| **Total Cholesterol (mmol/l)** | 6.1 | 1.1 | | 6.0 | 5.4 | 1.0 | | 5.4 | 5.9 | 1.1 | | 5.9 |
| **HDL Cholesterol (mmol/l)** | 1.6 | 0.4 | | 1.5 | 1.7 | 0.4 | | 1.6 | 1.6 | 0.5 | | 1.5 |
| **EQ-5D** | 0.840 | 0.245 | | 1.000 | 0.822 | 0.266 | | 1.000 | 0.787 | 0.265 | | 0.796 |
|  | Number | | Percentage | | Number | | Percentage | | Number | | Percentage | |
| **Diabetes** | 109 | | 4.3 | | 4814 | | 10.2 | | 121 | | 8.7 | |
| **Cardiovascular Disease** | 157 | | 6.1 | | 5476 | | 11.6 | | 139 | | 10.0 | |
| BMI Body Mass Index; SD Standard Deviation | | | | | | | | | | | | |

Table 61: Comparison of simulated outcomes from HSE 2003 with actual data from HSE 2011: Age group E - 60-69

| **E** | **HSE 2003: Before simulation (n=1968)** | | | | **HSE 2003: After simulation (n=43050)** | | | | **HSE 2011:**  **(n=989)** | | | |
| --- | --- | --- | --- | --- | --- | --- | --- | --- | --- | --- | --- | --- |
|  | **Age 60-69** | | | | **Age 68-77** | | | | **Age 68-77** | | | |
|  | Mean | SD | | Median | Mean | SD | | Median | Mean | SD | | Median |
| **Age (years)** | 64.2 | 2.9 | | 64.0 | 72.1 | 2.9 | | 72.0 | 72.2 | 2.8 | | 72.0 |
| **HbA1c (%)** | 5.6 | 0.7 | | 5.5 | 5.9 | 1.1 | | 5.7 | 6.1 | 0.9 | | 5.9 |
| **BMI kg/m^2^** | 28.1 | 4.6 | | 27.6 | 28.8 | 5.2 | | 28.2 | 28.5 | 4.9 | | 28.1 |
| **Systolic Blood Pressure (mm Hg)** | 137.8 | 18.9 | | 136.0 | 139.7 | 16.3 | | 139.5 | 134.5 | 17.0 | | 133.0 |
| **Total Cholesterol (mmol/l)** | 6.2 | 1.0 | | 6.2 | 5.2 | 1.1 | | 5.2 | 5.8 | 1.1 | | 5.8 |
| **HDL Cholesterol (mmol/l)** | 1.6 | 0.4 | | 1.5 | 1.6 | 0.4 | | 1.6 | 1.6 | 0.5 | | 1.5 |
| **EQ-5D** | 0.813 | 0.250 | | 0.848 | 0.790 | 0.272 | | 0.802 | 0.744 | 0.278 | | 0.796 |
|  | Number | | Percentage | | Number | | Percentage | | Number | | Percentage | |
| **Diabetes** | 165 | | 8.4 | | 6836 | | 15.9 | | 152 | | 15.4 | |
| **Cardiovascular Disease** | 299 | | 15.2 | | 9741 | | 22.6 | | 199 | | 20.1 | |
| BMI Body Mass Index; SD Standard Deviation | | | | | | | | | | | | |

Figure 13: Comparison of actual mean HbA1c levels from different age groups within HSE 2011, with predicted mean HbA1c levels after 8 years simulation using HSE 2003 baseline data.

Figure 14: Comparison of actual mean systolic blood pressure from different age groups within HSE 2011, with predicted mean systolic blood pressure after 8 years simulation using HSE 2003 baseline data.

Figure 15: Comparison of actual mean BMI from different age groups within HSE 2011, with predicted mean BMI after 8 years simulation using HSE 2003 baseline data.

Figure 16: Comparison of actual mean total cholesterol from different age groups within HSE 2011, with predicted mean total cholesterol after 8 years simulation using HSE 2003 baseline data.

Figure 17: Comparison of actual mean EQ-5D values from different age groups within HSE 2011, with predicted mean EQ-5D values after 8 years simulation using HSE 2003 baseline data.

Figure 18: Comparison of actual diabetes prevalence from different age groups within HSE 2011, with predicted diabetes prevalence after 8 years simulation using HSE 2003 baseline data.

Figure 19: Comparison of actual cardiovascular disease prevalence from different age groups within HSE 2011, with predicted cardiovascular disease prevalence after 8 years simulation using HSE 2003 baseline data.

## Predicting the Results of the ADDITION study

The ADDITION trial monitored metabolic risk factors, cardiovascular disease and mortality of recruited patients. We compared the data reported in key publications from the ADDITION trial to observe whether similar outcomes were identified in the simulation. The objective was to evaluate the model’s ability to simulate the results of a trial comparing an intensive intervention with usual care.

### Methods

ADDITION is a cluster-randomised controlled trial of screening for type 2 diabetes that took place in the UK (Cambridge and Leicester), Denmark and the Netherlands (79). The trial aimed to evaluate the efficacy and cost-effectiveness of population based screening for type-2 diabetes, and the effect of an intensive intervention compared with usual care for people diagnosed with diabetes. The second aspect of the study was used for validation of the model. Evaluation of the intensive intervention was based on cardiovascular outcomes data from the entire ADDITION trial (80).

Baseline patient level data for the study was obtained from the ADDITION authors and used to populate the model. Evaluation of the model’s ability to predict the effects of the intensive intervention was undertaken by comparing two versions of the model in which the individuals with diabetes from the ADDITION-Europe study were subjected either to usual care or to an intensive intervention. In the ADDITION-Europe trial, intensive intervention included extra sessions with the GP and practice nurse, referral to a dietician, intensive optimisation of cholesterol levels, blood pressure and blood glucose over the course of the first year after diagnosis, use of a glucometer and a pack of educational materials for the patient. Many of these features could not be added directly to the model, so instead the difference in five year metabolic outcomes between the two trial arms was used. The only metabolic parameters that showed significant changes in the study after five years of intensive intervention when compared with usual care were SBP (-2.86 mm Hg), total cholesterol (-0.27 mmol/l) and HbA1c (-0.08%). For the purposes of simulation, these changes were applied for the first five years to all individuals in the intensive treatment arm of the model, and then cardiovascular outcomes were assessed.

### Results & Discussion

In common with ADDITION-Cambridge, the SPHR Diabetes model does not predict a significant difference in mortality between screened and unscreened populations. Overall mortality rates are about one third higher in the Diabetes Prevention model than in the ADDITION-Cambridge study, indicating that the model is slightly over-predicting mortality.

The trajectories of all four metabolic parameters differ somewhat between the model and the ADDITION-Europe data (Table 62). In both arms of the ADDITION study, there is a reduction after diagnosis in the mean values of BMI, SBP, total cholesterol and HbA1c. However, in the SPHR Diabetes model there is an increase in HbA1c and BMI, whilst the reduction in SBP and total cholesterol is lower than that seen ADDITION-Europe. This indicates that the model may not be accurately reflecting improvements in health that occur as a consequence of normal care after diabetes diagnosis.

Table 62: Comparison of the Diabetes Prevention Model and the ADDITION-Europe study: metabolic data at baseline and after 5 years of follow-up/simulation

|  | **SPHR Diabetes Model** | | | | | | **ADDITION-Europe Trial** | | | | | | | |
| --- | --- | --- | --- | --- | --- | --- | --- | --- | --- | --- | --- | --- | --- | --- |
|  | **Before Simulation** | | **After Simulation** | | | | **Before Treatment** | | | | **After Treatment** | | | |
|  | Both Arms (n=50,000) | | Normal Care (n=50,000) | | Intensive Intervention (n=50,000) | | Normal Care (n=1,379) | | Intensive Intervention (n=1,678) | | Normal Care (n=1,285) | | Intensive Intervention (n=1,574) | |
|  | Mean | SD | Mean | SD | Mean | SD | Mean | SD | Mean | SD | Mean | SD | Mean | SD |
| **BMI (kg/m^2^)** | 31.6 | 5.5 | 32.3 | 5.8 | 32.3 | 5.8 | 31.6 | 5.6 | 31.6 | 5.6 | 31.0 | 5.6 | 31.1 | 5.7 |
| **SBP**  **(mm Hg)** | 149.0 | 21.5 | 146.2 | 19.9 | 143.8 | 19.7 | 149.8 | 21.3 | 148.5 | 22.1 | 138.1 | 17.6 | 134.8 | 16.8 |
| **Total Cholesterol (mm/l)** | 5.5 | 1.1 | 4.8 | 1.2 | 4.5 | 1.2 | 5.6 | 1.2 | 5.5 | 1.1 | 4.4 | 0.9 | 4.2 | 0.9 |
| **HbA1c (%)** | 7.0 | 1.5 | 7.7 | 0.8 | 7.6 | 0.8 | 7.0 | 1.5 | 7.0 | 1.6 | 6.7 | 0.95 | 6.6 | 0.95 |
| SPHR School for Public Health Research; ADDITION Anglo-Danish-Dutch Study of Intensive Treatment in People with Screen Detected Diabetes in Primary Care; BMI Body Mass Index; SBP Systolic Blood Pressure; HbA1c Glycated Haemoglobin; SD Standard Deviation | | | | | | | | | | | | | | |

In the ADDITION-Europe study, cardiovascular events, cardiovascular mortality and all-cause mortality were measured at the five year time point. Table 63 summarises these results and compares them with the simulated outcomes from the model. CVD and mortality are slightly over-predicted in both arms of the model compared with the trial, likely as a consequence of the higher metabolic values predicted by the model. However, the model predicts the slight but non-significant improvement in outcomes between the two arms of the trial fairly accurately.

Table 63: The ADDITION trial: comparison of simulated outcomes with the ADDITION-Europe study

|  | **SPHR Diabetes Model** | | | | **ADDITION-Europe** | | | |
| --- | --- | --- | --- | --- | --- | --- | --- | --- |
|  | Normal Care | Intensive Intervention | Hazard Ratio | | Normal Care | Intensive Intervention | Hazard Ratio | |
|  | Percentage | Percentage | Mean | 95% CI | Percentage | Percentage | Mean | 95% CI |
| **Cardiovascular events** | 9.9 | 9.5 | 0.96 | 0.93-1.00* | 8.5 | 7.2 | 0.83 | 0.65-1.05 |
| **Cardiovascular mortality** | 2.8 | 2.7 | 0.95 | 0.88-1.02 | 1.6 | 1.5 | 0.88 | 0.51-1.51 |
| **All cause mortality** | 9.0 | 8.8 | 0.98 | 0.94-1.03 | 6.7 | 6.2 | 0.91 | 0.69-1.21 |
| CI 95% Confidence Interval *The upper bound for the 95% CI is slightly below 1, meaning that the intervention is just significantly different at p=0.05 | | | | | | | | |

## UKPDS Major Events in Diabetes

The UKPDS has recorded long-term outcomes for individuals with diabetes in the UK. It is currently used in many economic models of diabetes to predict the incidence of micro- and macro-vascular events as well as mortality in diabetes. In the SPHR diabetes model it is only used to estimate the incidence of renal failure, blindness, amputation and ulcers. This validation is hence an internal validation for the microvascular outcomes of the SPHR model, and an external validation for other outcomes. The aim was to evaluate if the incidence of microvascular, macrovascular and fatal complications of diabetes are similar to those estimated in the UKPDS outcomes model.

### Methods

The UKPDS outcomes model 2 reports the simulated percentage of individuals with major events after 10 years from the UKPDS model (20). The SPHR diabetes model was tested by generating 50,000 individuals aged between 25 and 65 with diabetes from the HSE 2011. The model was run over a time course of 10 years to obtain figures for 10 year prevalence.

### Results & Discussion

The incidences of major events in the UKPDS Outcomes model and the SPHR diabetes model are reported in Table 64.

Table 64: Major events in the UKPDS and SPHR simulation

| **10 year prevalence (%)** | **UKPDS Outcomes Model 2 (N=3984)** | **SPHR model (N=50000)** |
| --- | --- | --- |
| Renal Failure | 0.5 | 0.4 |
| Ulcer | 1.8 | 1.8 |
| Amputation | 1.5 | 1.9 |
| 2^nd^ Amputation | 0.44 | 0.6 |
| Blindness | 2.9 | 3.0 |
| MI | 9.9 | 5.1 |
| Stroke | 6.2 | 6.1 |
| Heart Failure | 4 | 5.1 |
| Death | 22.5 | 13.9 |

The validation indicates that the SPHR Diabetes model predicts very similar 10 year prevalence values to the UKPDS outcomes model for those outcomes which are determined through the UKPDS itself. The exception to this is amputation, which is over-predicted in the SPHR model compared with the UKPDS model. In the UKPDS outcomes model, amputation is specified using a different algorithm for people with a pre-existing ulcer or for those without. To simplify things, the SPHR diabetes model uses a single algorithm to specify amputation, with the addition of an extra parameter for pre-existing ulcer estimated from the difference in the hazard ratio for amputation between individuals with or without pre-existing ulcer in the UKPDS. This is likely to be the source of the discrepancy in estimation of amputation prevalence, as alteration of this parameter has a large effect on prevalence of amputation (data not shown). However, overall this validation indicates that the UKPDS model has been correctly implemented in the SPHR Diabetes model.

The UKPDS model predicts a higher incidence of MI than is simulated in the SPHR model using the QRISK algorithm to predict first cardiovascular event. Other studies have found that the UKPDS Outcomes model v1 predicts higher risks for these events than are observed in other datasets (81), although the new UKPDS outcomes model v2 has been shown to predict lower incidence of these outcomes than the UKPDS model v1. The lower still prediction of the SPHR model may reflect recent medical developments. Alternatively, given that the predictions for stroke are fairly accurate, the discrepancy may be in the way that CVD events are distributed rather than the number of CVD events per se.

The incidence of heart failure is higher in the SPHR simulation compared with the UKPDS. The incidence of congestive heart failure is simulated from the Framingham risk algorithm in men and women. It is possible that the higher incidence of heart failure is due to differences in the risk of heart failure in the UK and US. We have sought a UK algorithm for estimating the risk of congestive heart failure, however none were found. We considered whether it would be best to change the model to use the UKPDS equation only in diabetic patients. However, we dismissed this idea because this would assume that non-diabetics were not at risk of congestive heart failure. However, it is likely that the SPHR model currently over-estimates the incidence of congestive heart failure.

Finally, mortality is under-predicted in the SPHR model compared with the UKPDS outcomes model. One potential cause of this discrepancy is the incorporation in the SPHR model of improvements in diabetes care that may have been made since the UKPDS trial. Given that the SPHR model actually over-estimates mortality in some of the other validations (e.g. ADDITION) this is probably not a concern.

# Diabetes Prevention Interventions

## Selecting Interventions to be included in the Model

Interventions were identified with the input from a group of project stakeholders who included clinicians, patients with diabetes and public health policy makers. Potential interventions were divided into the following population groups:

1. For the general population to reduce risk factors for diabetes;
2. For people with non-diabetic hyperglycaemia;
3. For people within the general population who are at high risk of developing non-diabetic hyperglycaemia and type 2 diabetes, including identification and risk assessment (eg. overweight or obese, low socioeconomic status, South Asian, those with cardiovascular disease, those picked up by health checks).

Systematic reviews produced for NICE projects already exist for (B) and (C) above (3;4). No evidence on walking and cycling/ transport policy interventions was identified within the review of systematic reviews; however an existing NICE report describes a recent review of this area (82).

A literature review was undertaken for population-level interventions (A above). A search was also done for evidence relating to population level (i.e. not targeted) interventions to either prevent pre-diabetes or obesity. Due to the numerous potential interventions suggested within the first stakeholder workshop and the fact that the search was designed to identify all interventions, an approach was followed which combined terms for individuals at risk of developing diabetes and terms for prevention of diabetes/obesity. A decision was made to focus on systematic reviews of population level interventions, therefore a systematic review filter was applied to the search. This literature search was undertaken in Medline and Medline in Process via OVID in October 2012. Retrieved references were imported into Reference Manager and sifted for inclusion in the review.

Following on from the database search, reference lists of the relevant systematic reviews were scutinised for other systematic reviews and individual studies. Cited reference searching was undertaken for included systematic reviews and a search was undertaken on Google to identify any available full reports of systematic reviews. A targeted Google search was also undertaken to supplement the database search as outlined above.

Given the heterogeneity in the studies identified from these systematic reviews it was not possible to conduct a meta-analysis of interventions. Instead, example interventions were selected by the stakeholder group to represent interesting policies and demonstrate the capacity of the model for future policy questions. The details of the interventions identified in the search and from previous reviews were presented to the project stakeholders at a workshop. The stakeholder’s discussion was used to select the final list of interventions to include in the analysis. The stakeholders stressed the desirability of presenting a spectrum of intervention types, and the discussion focused on taxation, community education, agricultural policy, food retailer interventions, physical activity for transport, workplace interventions and risk assessment. Given the constraints of the project there was a need to limit the interventions included within the final model and based upon the discussion within the workshop a subset of interventions were selected for inclusion in the model. Table 65 reports the intervention types selected for inclusion and exclusion.

At the national level a taxation policy was chosen. Evidence for the effectiveness of the intervention was available and modelling studies estimated the price elasticity of taxable products (83). A concern was raised around considering taxation due to (i) the possibility of consumption of poor alternatives and (ii) implementation issues given the power of the food industry. The former was addressed by using evidence which reports alternative consumption and including this within the model. Agricultural policy was not included at this point in the project, mainly due to the absence of evidence and the complexity of the systems relating policy to individual consumption. However, it could be incorporated into the model in the future.

At the community level workplace interventions, local transport policy, retailer policy and community education programmes were considered. Local transport policy was not included in the final analysis.

At the individual level, those identified as high-risk would be identified through a risk assessment and blood test strategy. Of the other targeted groups identified in the stakeholder meeting it was decided to exclude children (and other primordial prevention rather than primary prevention), due to the added complexity of modelling a life course.

Table 65: Types of interventions considered for inclusion in the model

| **Intervention Coverage** | **Selected for Inclusion** | **Selected for Exclusion** |
| --- | --- | --- |
| General Population (Indiscriminate National Policy) | Taxation | Agricultural Policy |
| Communities | Workplace | Transport policy |
|  | Retailer policy |  |
|  | Community education programme |  |
| High-risk individuals* | Non-diabetic hyperglycaemic | Children/ early years |

*These will be interventions that are feasible within the real world (translational).

## Intervention A: Soft-Drinks Tax

### Effectiveness

The effect of soft drinks taxation on BMI by age group and income has been estimated in a comprehensive modelling exercise (83). The effect on people aged 50 or above is not significant so was assumed to be zero in the base case, but allowed to vary in the probabilistic sensitivity analysis. These estimates were implemented straight into the Diabetes model without further assumptions.

Table 66: Change in BMI by age given a 20% tax on sugar sweetened soft drinks (98)

|  | 16-29 year olds | 30-49 year olds | >=50 year olds |
| --- | --- | --- | --- |
| Change in BMI | -0.23 (-0.28 to -0.20) | -0.05 (-0.07 to -0.03) | 0.01 (-0.01 to 0.03) |

The impact of BMI was not assumed to vary by socioeconomic status because the impact on BMI did not vary substantially across income groups (83). The price elasticities used in Briggs et al. are reported below in Table 67.

Table 67: Estimated own price and cross price elasticity values for drinks from Briggs et al. (83)

| Drink group | Milk | Water | Fruit juice | Diet soft drink (concentrated) | SSDs (concentrated) | Diet soft drinks (non-concentrated) | SSD (non-concentrated) | Tea and coffee | Other drinks | Beer | Wine | Other alcohol |
| --- | --- | --- | --- | --- | --- | --- | --- | --- | --- | --- | --- | --- |
| Milk | -0.981 | 0.002 | 0.008 | 0.012 | 0.032 | 0.070 | 0.157 | 0.165 | 0.017 | 0.002 | 0.003 | 0.002 |
| Water | 0.016 | -1.174 | 0.009 | 0.008 | 0.021 | 0.051 | 0.098 | 0.189 | 0.019 | 0.001 | 0.002 | 0.01 |
| Fruit juice | 0.025 | 0.003 | -0.971 | 0.010 | 0.027 | 0.058 | 0.128 | 0.121 | 0.013 | 0.001 | 0.002 | 0.001 |
| Diet soft drink (concentrated) | 0.019 | 0.002 | 0.005 | -0.979 | 0.304 | 0.082 | 0.073 | 0.110 | 0.011 | 0.001 | 0.002 | 0.001 |
| SSDs (concentrated) | 0.020 | 0.002 | 0.005 | 0.107 | -0.921 | 0.100 | 0.179 | 0.112 | 0.012 | 0.001 | 0.002 | 0.001 |
| Diet soft drinks (non-concentrated) | 0.026 | 0.002 | 0.007 | 0.005 | 0.027 | -0.903 | 0.167 | 0.147 | 0.016 | 0.002 | 0.003 | 0.002 |
| SSD (non-concentrated) | 0.027 | 0.002 | 0.008 | -0.004 | 0.013 | 0.063 | -0.811 | 0.154 | 0.016 | 0.002 | 0.003 | 0.002 |
| Tea and coffee | 0.031 | 0.005 | 0.007 | 0.013 | 0.035 | 0.076 | 0.168 | -0.912 | -0.006 | 0.002 | 0.002 | 0.002 |
| Other drinks | 0.016 | 0.003 | 0.004 | 0.007 | 0.018 | 0.038 | 0.089 | 0.340 | -0.822 | 0.001 | 0.001 | 0.001 |
| Beer | -0.001 | 0.000 | 0.000 | -0.001 | -0.004 | -0.006 | -0.007 | -0.001 | -0.001 | -0.921 | 0.032 | 0.016 |
| Wine | -0.001 | 0.000 | -0.001 | -0.001 | -0.002 | -0.005 | -0.008 | -0.008 | -0.001 | 0.022 | -1.009 | 0.021 |
| Other alcohol | -0.01 | 0.000 | 0.000 | -0.001 | -0.001 | -0.003 | -0.006 | -0.006 | -0.001 | 0.018 | 0.035 | -0.969 |

In Briggs et al. the price elasticities are applied to the Living Costs and Food Survey to estimate the change in sugar sweetened beverages purchased at the household level. Percent changes in purchases are translated to percent changes consumption at the individual using the National Diet and Nutrition Survey by assuming the same change in consumption as purchases. They used the change in volume of drinks consumed to estimate the change in the energy intake, using measures of the average calorie density of beverages, as reported in the National Diet and Nutrition Survey. The final step in the model estimates the change in the distribution of body mass index as a result of the change in the mean energy intake on the basis of the assumption that body mass index in the population follows a log-normal distribution and that the variance of body mass index in the population is unaffected by the change in energy intake.

The Whitehall II statistical models estimate an indirect effect from the intervention modifying BMI on systolic blood pressure, total cholesterol and HbA1c trajectories. The indirect effects of the intervention are reported in Table 68.

Table 68: Implied indirect changes in other metabolic risk factors

|  | 16-29 year olds | 30-49 year olds |
| --- | --- | --- |
| Change in BMI (kg/m^2^) | -0.23 | -0.05 |
| Indirect change in Systolic blood pressure (mmHg) | -0.053 | -0.012 |
| Indirect change in Total Cholesterol (mmol/L) | -0.022 | -0.005 |
| Indirect change in HbA1c (%) | -0.002 | -0.001 |

### Population

The soft drinks taxation policy was applied to the general population; however the effectiveness of the intervention was conditional on the age of the individual at the start of the model.

### Cost

The soft drinks taxation was assumed to not incur any costs. In theory, taxation would probably generate additional income, but we decided that it was outside the scope of the model to estimate its value.

## Intervention B: Fruit and Vegetable Retail provision

### Effectiveness

The Wrigley Leeds Tesco store opening was studied to observe the impact on the local community’s fruit and vegetable consumption. The results informed the formulation of a regression model to predict change in fruit and veg after the store opened (84). Using the data reported in this study it was estimated that the mean increase in fruit and vegetables consumed was 0.162 portions per day^[[2]](#footnote-2)^.

The evidence for relating a change in fruit and vegetable consumption to a change in BMI is contradictory. We instead decided to relate changes in fruit and vegetable consumption directly to changes in HbA1c and systolic blood pressure using data from two different studies.

A cross-sectional analysis from the European Prospective Investigation into Cancer and Nutrition in Norfolk (EPIC-Norfolk) investigated how plasma vitamin C levels relate to HbA1c (85). The study reported the results of a linear regression, which shows that a 20µmol/l increase in plasma vitamin C is associated with a reduction in HbA1c of 0.08% for men and 0.05% for women, when adjusted for possible confounders including age and BMI. According to the study, a 20µmol/l increase in plasma vitamin C is equivalent to eating an extra orange per day. Assuming that the vitamin C in one orange is equivalent to the vitamin C in one portion of fruit or veg, and taking the weighted mean for men and women, we estimated that the retail policy would reduce HbA1c by an average of 0.010% per person.

A randomised controlled clinical trial testing the efficacy of an intervention promoting consumption of fruit and vegetables, found that there was a mean increase of 1.4 portions of fruit or vegetables consumed per day in the intervention group compared with the control group (86). This was associated with a reduction in systolic blood pressure of 4.0 mm Hg. Implementing this value straight into the diabetes model suggested that the retail policy would reduce blood pressure by 0.46 mm Hg.

### Population

We applied this intervention to individuals in the highest quintile of the Townsend deprivation score, as these people are more likely to have inadequate access to fruit and vegetable provision.

### Costs

The costs of this intervention were assumed to be incurred by the private sector and were not included in the analysis. Therefore, the evaluation only considered the health gains of the policy.

## Intervention C: Worksite environment

### Effectiveness

The Heartbeat Award scheme implemented healthy food options in cafeterias in the workplace and observed the impact on workers dietary patterns before and after the menu changes (87). The results of the study reported the proportion of individuals who made a positive switch to healthier food options after the changes in the workplace café. The proportions were compared between participating and non-participating workplaces using odds ratios. The four food groups that demonstrated a significant improvement over the study were sweet puddings, fried food, fruit and milk. The magnitude of improvement or worsening was not reported in the statistical analysis. The benefits of the work place intervention were measured in terms of the increase in fruit consumption and the switching of milk from a higher to a lower fat choice. We decided not to account for the reduction in fried food and sweet puddings due to a lack of evidence about nutritional content and food substitution.

The study did not estimate the mean change in fruit and vegetable consumption for the 11.9% of individuals who made a positive change. Therefore, it was assumed that they increased their consumption of fruit by one portion per day. We used the same assumptions and evidence to translate change in fruit and vegetable consumption to HbA1c and systolic blood pressure that were described for the retail provision intervention (85;86); this translated to a mean reduction in HbA1c of 0.063%, and in systolic blood pressure of 2.86 mm Hg for the 11.9% who were reported as eating more fruit and vegetables.

8.9% of individuals were reported as switching their milk choice from a higher fat to a lower fat option. Milk choices were not documented, so it was assumed that individuals switched from full fat milk to lower fat milk choices based on population-wide consumption of milk types (Table 68). Calorie and fat content in different milk types was obtained from the Dairy Council (88). The quantity of milk drunk by each individual was assumed to be the population mean of 1506ml per week; this value was obtained from the Defra Family Food Survey 2012 (88).

Table 69: Nutritional content and consumption of different milk types (89)

| **Type of Milk** | Fat Content (g/100ml) | Saturated Fat (g/100ml) | Calories (per 100ml) | Consumption (% consumers) |
| --- | --- | --- | --- | --- |
| Full fat | 4.0 | 2.6 | 68 | 23 |
| Semi-skimmed | 1.8 | 1.1 | 47 | 63 |
| Skimmed | 0.3 | 0.1 | 35 | 6 |
| 1% | 1.0 | No data | No data | No data |

It was assumed that fat consumption would reduce due to milk switching by the mean change in fat content of full fat milk, compared with the weighted mean of lower fat alternatives. The mean reduction in fat consumption was calculated as 2.33g per 100ml of milk, or 5.01g per day.

Evidence was available from a cross-sectional study from EPIC-Norfolk to relate HbA1c levels to dietary fat consumption as a percentage of daily calories (90). We estimated that mean daily fat intake would drop from 32% to 29.8% of total daily calories as a result of milk switching, which corresponds to a reduction in HbA1c of 0.0156%.

Table 70: Dietary fat consumption and changes in HbA1c (90)

| **Independent Variable** | **Regression coefficients (per 1 SD change in fat)** | **P** | **Mean daily intake (weighted male and female)** | **Standard Deviation** |
| --- | --- | --- | --- | --- |
| Total Fat | 0.0420 | <0.001 | 32% | 5.9% |
| Saturated Fat | 0.0476 | <0.001 | 12.5% | 3.4% |
| Ratio Polyunsaturated Fat to Saturated Fat | -0.0200 | 0.013 | 0.51 | 0.22 |

### Population

We applied this intervention to randomly selected individuals in employment. We assumed that 20% of workplaces in the population have canteens which adopt the intervention. However, only 11.9% of individuals in the workplace were assumed to respond positively to the programme in terms of fruit consumption, and 8.9% of individuals were assumed to respond positively in terms of milk switching. Random selection of individuals was independent for the two responses.

### Costs

The cost of the Heartbeat Award Scheme includes the cost of the environmental health officer to visit the premises and inspect menu changes for healthy eating options. The health authority also issue promotional material and certificates to the workplace and these printing costs were factored into the overall intervention cost (Table 70).

Table 71: Cost estimates for the Heartbeat Award Scheme

| Cost type | Description | Unit cost |
| --- | --- | --- |
| Personnel costs | Environment health officer to inspect establishments, and assess menus. A week of work per workplace valued at the UK average salary. | £474 |
| Printing costs | Posters, leaflets, door stickers, flyers, certificates | £25 |
| Total Cost per workplace | | £499 |
| Per capita cost (assuming 100 employees per workplace) | | £4.99 |

## Intervention D: Community Education Programmes

### Effectiveness

We identified three community education programmes that could be included in the model to describe the effectiveness of targeted education interventions in “at risk” communities. Community nurses working in partnership with a community dietician in Camelon, a deprived area of Scotland, developed a group-based weight management intervention specifically for obese men (91). The second intervention was a Mediterranean diet class for socially deprived women with Rheumatoid Arthritis (92). The third intervention was a food skills intervention for individuals from urban deprived communities and was not included in the final analysis as it only reported changes in fruit and veg consumption and there were no significant differences in these outcomes at 6 months follow-up (93). As a consequence, we used the other two intervention programmes as an example of the effectiveness of community programmes in men and women respectively. A summary of how the interventions were added to the model is provided in Table 71. The increase in fruit and vegetable consumption was assumed to produce direct effects on HbA1c (-0.09%) and systolic blood pressure (-0.41 mm Hg) independently of the effects on BMI, in the same way as described in the fruit and vegetable retail provision intervention above (85;86).

Table 72: Estimates and assumptions applied in the model for community interventions

|  | Eligible | Uptake | Change in BMI | Change in fruit and veg | Assumptions |
| --- | --- | --- | --- | --- | --- |
| Mediterranean | Females  in  highest deprivation quintile | Assumed 11.4% to align with men | -1.04kg/m^2^ | 0.143 extra portions per day | 1. No compliance data reported.  2. Benefit at 6 months maintained to 12 months.  3. Applied to non-Rheumatoid arthritis population  4. Applied to highest quintile of Townsend score. |
| Men’s diets | Men  >30kg/m^2^ | 11.4% | -1.29 kg/m^2^ | Assumed 0.143 extra portions per day to align with women. | 1. Benefit at 6 months maintained to 12 months.  2. Applied to men with BMI>30 kg/m^2^ |

The Whitehall II statistical models estimate an indirect effect from the intervention modifying BMI on systolic blood pressure, total cholesterol and HbA1c trajectories. The indirect effects for HbA1c and systolic blood pressure were adjusted for the direct effects described above to avoid double counting. The indirect effects of the intervention are reported in Table 73.

Table 73: Implied indirect changes in other metabolic risk factors

|  | Men’s diets | Mediterranean |
| --- | --- | --- |
| Change in BMI (kg/m^2^) | -1.29 | -1.04 |
| Indirect change in Systolic blood pressure (mmHg) | -0.293 | -0.242 |
| Indirect change in Total Cholesterol (mmol/L) | -0.123 | -0.102 |
| Indirect change in HbA1c (%) | -0.013 | -0.010 |

### Population

These interventions were combined such that within the same analysis, women with the highest deprivation quintile were offered a cooking class, whilst men with a BMI >30kg/m^2^ were offered the multi-component small scale diet programme. The assumed uptake rates for these interventions are reported in Table 71.

### Costs

The interventions described in Table 71 were previously evaluated as part of the NICE public health guidance (PH35) (3). In this evaluation the estimated costs of the intervention were £82 for the Mediterranean cooking class and £179 for the men’s diets per participant.

## Intervention E: Translational Diabetes Prevention Programme

### Effectiveness

A meta-analysis of translational diabetes prevention programmes was used to estimate the change in BMI, HbA1c, systolic blood pressure and cholesterol at 12 months (94). The review included studies that had run diet and or exercises classes for individuals with a high risk of diabetes. The definition of risk of diabetes varied between studies, but many included risk classification based on increased blood glucose. The review reported mean changes in metabolic measurements at 12 months (Table 72). In the model, the intervention was offered to individuals with impaired glucose regulation. The change in BMI was taken directly from the meta-analysis. The change in HbA1c, systolic blood pressure and total cholesterol were adjusted down to reflect the independent effect over and above the effect of changes in BMI estimated using the Whitehall statistical model. This avoided double counting of treatment benefits.

Table 73: Metabolic changes 12 months after diet and exercise interventions for individuals at high risk of diabetes

|  | BMI (kg/m^2^) | HbA1c (%) | Systolic BP (mm Hg) | Total Cholesterol (mmol/l) |
| --- | --- | --- | --- | --- |
| 12 months after intervention | -0.94 | -0.121 | -0.1975 | -0.098 |

### Population

Individuals were assumed to be identified as part of the NHS Vascular checks programme. Adults aged 40-65 in the Health Survey for England were assumed to be invited to the NHS Vascular Checks programme. Attendance at the vascular checks were assumed to be 43.7% based on a review of NHS health checks in Stoke on Trent (95). An individual’s risk of diabetes was assessed using the Leicester Risk Score (96) whilst attending the vascular checks (94), and was invited for diabetes screening if the score was greater than 4·75. Individuals who attended screening with HbA1c >=47.5 mmol/mol (6·5%) were diagnosed with diabetes. Individuals with HbA1c>=42.5mmol/mol (6·0%), and not diagnosed with diabetes were offered the lifestyle programme. An intervention uptake rate of 32% was assumed based on estimates from Public Health England (97).

### Costs

The intervention costs were designed to replicate the costing methods used in the NICE Public Health guidance (PH38) (4). Given the -2.12kg mean weight loss, this intervention most closely matched to the moderate intensity intervention described in the guideline which cost £100 per individual in the first year. We assumed that individuals received 6 monthly maintenance classes after the visits in years 2-4 at a cost of £60 per year.

## Maintenance of Intervention Effects

Ideally, weight regain rates and the altered trajectories of HbA1c and systolic blood pressure would be modelled separately for each intervention based upon long term follow-up data. Unfortunately, this data was not available for most of the interventions considered. With this in mind, we decided to apply the full effectiveness of each intervention for the first year only, then in subsequent years, to assume that effectiveness would diminish linearly, reaching zero effect after 5 years.

## Layering Interventions

The model is sufficiently flexible to enable layering of interventions in order to determine which combinations are highly cost-effective and which combinations could be used to efficiently target certain subpopulations. Interventions can be layered in several different ways, to reflect what will occur when an individual is subject to more than one intervention. Layering can be considered to be either additive, synergistic (i.e. greater than additive), antagonistic (multiple interventions result in less effect than a single intervention) or it may have an effect that is somewhere between antagonistic and additive (one example being that the individual might only obtain an affect from one of the layered interventions).

# Probabilistic Sensitivity Analysis

Probabilistic sensitivity analysis (PSA) was enabled in the model to describe the uncertainty in parameter inputs of the model and how this translates into uncertainty in the outcomes of the model. A suitable distribution was selected for each parameter, based upon its mean and standard error. Random sampling simultaneously across all input parameter distributions allowed parameter uncertainty to be quantified. 1000 different random samples of parameter values were selected, and each was applied to a different random cohort of 20,000 individuals. For each PSA sample, the model was run and results compiled. Given the large number of parameters in the model and thus the capacity for error, a thorough process of checking that mean sampling values corresponded to mean parameter values was undertaken to ensure that the results were as accurate as possible. A list of model parameters, their distribution for PSA and their source is provided in the following tables.

## GP Attendance in the General Population

In the probabilistic sensitivity analysis the parameters of the Yorkshire Health Study negative binomial model are sampled from a multivariate normal distribution, using the mean estimates described in Table 15 and covariance matrix in Table 16.

Table 74: GP attendance reported in the Yorkshire Health Study (N= 18,437) (10)

|  | Mean | Standard error | Uncertainty Distribution |
| --- | --- | --- | --- |
| Age | 0.0076 | 0.0005 | MULTIVARIATE NORMAL |
| Male | -0.1495 | 0.0159 | MULTIVARIATE NORMAL |
| BMI | 0.0110 | 0.0015 | MULTIVARIATE NORMAL |
| Ethnicity (Non-white) | 0.2620 | 0.0375 | MULTIVARIATE NORMAL |
| Heart Disease | 0.2533 | 0.0289 | MULTIVARIATE NORMAL |
| Depression | 0.6127 | 0.0224 | MULTIVARIATE NORMAL |
| Osteoarthritis | 0.2641 | 0.0238 | MULTIVARIATE NORMAL |
| Diabetes | 0.2702 | 0.0278 | MULTIVARIATE NORMAL |
| Stroke | 0.1659 | 0.0474 | MULTIVARIATE NORMAL |
| Cancer | 0.2672 | 0.0414 | MULTIVARIATE NORMAL |
| Intercept | -0.5014 | 0.0468 | MULTIVARIATE NORMAL |
| Alpha | 0.3423 | 0.0108 | MULTIVARIATE NORMAL |

Table 75: Variance-covariance matrix for GP attendance regression

|  | Age | Male | BMI | Ethnicity (Non-white) | Heart Disease | Depression | Osteo-arthritis | Diabetes | Stroke | Cancer | Intercept | Alpha |
| --- | --- | --- | --- | --- | --- | --- | --- | --- | --- | --- | --- | --- |
| Age | 0.0000 |  |  |  |  |  |  |  |  |  |  |  |
| Male | 0.0000 | 0.0003 |  |  |  |  |  |  |  |  |  |  |
| BMI | 0.0000 | 0.0000 | 0.0000 |  |  |  |  |  |  |  |  |  |
| Ethnicity (Non-white) | 0.0000 | 0.0000 | 0.0000 | 0.0014 |  |  |  |  |  |  |  |  |
| Heart Disease | 0.0000 | 0.0000 | 0.0000 | 0.0000 | 0.0008 |  |  |  |  |  |  |  |
| Depression | 0.0000 | 0.0000 | 0.0000 | 0.0000 | 0.0000 | 0.0005 |  |  |  |  |  |  |
| Osteoarthritis | 0.0000 | 0.0000 | 0.0000 | 0.0000 | 0.0000 | 0.0000 | 0.0006 |  |  |  |  |  |
| Diabetes | 0.0000 | 0.0000 | 0.0000 | 0.0000 | -0.0001 | 0.0000 | 0.0000 | 0.0008 |  |  |  |  |
| Stroke | 0.0000 | 0.0000 | 0.0000 | 0.0000 | -0.0002 | -0.0001 | 0.0000 | -0.0001 | 0.0022 |  |  |  |
| Cancer | 0.0000 | 0.0000 | 0.0000 | 0.0000 | 0.0000 | 0.0000 | 0.0000 | 0.0000 | -0.0001 | 0.0017 |  |  |
| Intercept | 0.0000 | 0.0000 | -0.0001 | -0.0002 | 0.0002 | 0.0000 | 0.0002 | 0.0003 | 0.0000 | 0.0001 | 0.0022 |  |
| Alpha | 0.0000 | 0.0000 | 0.0000 | 0.0000 | 0.0000 | 0.0000 | 0.0000 | 0.0000 | 0.0000 | 0.0000 | 0.0000 | 0.0010 |

## Whitehall II Statistical Model of Metabolic Trajectories

The parameters derived from the Whitehall II statistical model of metabolic trajectories are described in Table 76 and Table 78.

Table 76: Coefficient estimates for metabolic risk factor parallel growth models

|  | **Parameter Description** | **Estimated Mean** | **Standard error** | **p-value** |
| --- | --- | --- | --- | --- |
| BMI Intercept | |  |  |  |
| $\alpha_{10}$ | Population mean BMI intercept | 2.2521 | 0.045 | <0.001 |
| $\boldsymbol{\gamma}_{\boldsymbol{10}}$ | Age at baseline coefficient for BMI intercept | 0.0056 | 0.001 | <0.001 |
|  | Sex coefficient for BMI intercept | -0.0311 | 0.012 | 0.009 |
|  | Family history of CVD coefficient for BMI intercept | -0.0079 | 0.012 | 0.515 |
| $\upsilon_{10}$ | Random error term for BMI intercept | 0.1165 | 0.003 | <0.001 |
| BMI linear slope | |  |  |  |
| $\alpha_{11}$ | Population mean BMI linear slope | 0.6409 | 0.042 | <0.001 |
| $\boldsymbol{\gamma}_{\boldsymbol{11}}$ | Age at baseline coefficient for BMI linear slope | -0.0084 | 0.001 | <0.001 |
|  | Sex coefficient for BMI linear slope | -0.0285 | 0.011 | 0.009 |
|  | Family history of CVD coefficient for BMI linear slope | -0.0155 | 0.010 | 0.117 |
| $\upsilon_{11}$ | Random error term for BMI linear slope | 0.0222 | <0.001 | <0.001 |
| BMI quadratic slope | |  |  |  |
| $\alpha_{12}$ | Population mean BMI quadratic slope | -0.2007 | 0.023 | <0.001 |
| $\boldsymbol{\gamma}_{\boldsymbol{12}}$ | Age at baseline coefficient for quadratic slope | 0.0026 | <0.001 | <0.001 |
|  | Sex coefficient for quadratic slope | 0.0089 | 0.006 | 0.147 |
|  | Family history of CVD coefficient for quadratic slope | 0.0104 | 0.006 | 0.061 |
| $\varepsilon_{1}$ | Random error term for BMI | 0.0104 | <0.001 | <0.001 |
| Glyc Intercept | |  |  |  |
| $\alpha_{20}$ | Population mean glyc intercept | 0 | NA | NA |
| $\boldsymbol{\gamma}_{\boldsymbol{20}}$ | Smoker coefficient for glyc intercept | -0.1388 | 0.029 | <0.001 |
| $\tau_{20}$ | Association between BMI intercept and glyc intercept | 0.2620 | 0.024 | <0.001 |
| $\upsilon_{20}$ | Random error term for glyc intercept | 0.0851 | 0.008 | <0.001 |
| Glyc linear slope | |  |  |  |
| $\alpha_{21}$ | Population mean glyc linear slope | -0.4255 | 0.071 | <0.001 |
| $\boldsymbol{\gamma}_{\boldsymbol{21}}$ | Sex coefficient for glyc linear slope | 0.1486 | 0.045 | 0.001 |
|  | Ethnicity coefficient for glyc linear slope | -0.0218 | 0.081 | 0.786 |
|  | Family history of T2DM coefficient for glyc linear slope | -0.0512 | 0.054 | 0.345 |
|  | Smoker coefficient for glyc linear slope | 0.1796 | 0.066 | 0.007 |
| $\tau_{21}$ | Association between BMI intercept and glyc linear slope | 0.0821 | 0.024 | 0.001 |
| $\tau_{22}$ | Association between BMI linear slope and glyc linear slope | 0.1984 | 0.073 | 0.007 |
| $\upsilon_{21}$ | Random error term for glyc linear slope | 0.0222 | 0.011 | 0.053 |
| Glyc quadratic slope | |  |  |  |
| $\alpha_{22}$ | Population mean glyc quadratic slope | 0.1094 | 0.025 | <0.001 |
| $\boldsymbol{\gamma}_{\boldsymbol{22}}$ | Sex coefficient for glyc quadratic slope | -0.0855 | 0.027 | 0.002 |
|  | Ethnicity coefficient for glyc quadratic slope | 0.0899 | 0.049 | 0.067 |
|  | Family history of T2DM coefficient for glyc quadratic slope | 0.0633 | 0.033 | 0.052 |
|  | Smoker coefficient for glyc quadratic slope | -0.0390 | 0.040 | 0.330 |
| $\upsilon_{22}$ | Random error term for glyc quadratic slope | 0.0107 | 0.003 | 0.002 |
| $\varepsilon_{2}$ | Glyc measurement error | 0.0707 | 0.005 | <0.001 |
| SBP Intercept | |  |  |  |
| $\alpha_{30}$ | Population mean SBP intercept | 0.6934 | 0.021 | <0.001 |
| $\boldsymbol{\gamma}_{\boldsymbol{30}}$ | Age at baseline coefficient for SBP intercept | 0.0043 | <0.001 | <0.001 |
|  | Sex coefficient for SBP intercept | 0.0380 | 0.004 | <0.001 |
|  | Smoking coefficient for SBP intercept | -0.0243 | 0.006 | <0.001 |
|  | Ethnicity coefficient for SBP intercept | 0.0078 | 0.007 | 0.300 |
|  | Family history of CVD coefficient for SBP intercept | 0.0061 | 0.004 | 0.160 |
| $\boldsymbol{\tau}_{\boldsymbol{31}}$ | Association between BMI intercept and SBP intercept | 0.1080 | 0.006 | <0.001 |
| $\upsilon_{30}$ | Random error term for SBP intercept | 0.0085 | 0.00 | <0.001 |
| SBP linear slope | |  |  |  |
| $\alpha_{31}$ | Population mean SBP linear slope | -0.0227 | 0.021 | 0.278 |
| $\boldsymbol{\gamma}_{\boldsymbol{31}}$ | Age at baseline coefficient for SBP linear slope | 0.0024 | <0.001 | <0.001 |
|  | Sex coefficient for SBP linear slope | -0.0004 | 0.004 | 0.927 |
|  | Smoking coefficient for SBP linear slope | 0.0205 | 0.005 | <0.001 |
|  | Ethnicity coefficient for SBP linear slope | 0.0224 | 0.007 | 0.001 |
|  | Family history of CVD coefficient for SBP linear slope | -0.0013 | 0.004 | 0.748 |
| $\boldsymbol{\tau}_{\boldsymbol{31}}$ | Association between BMI intercept and SBP linear slope | -0.0396 | 0.006 | <0.001 |
|  | Association between BMI linear slope and SBP linear slope | 0.2325 | 0.019 | <0.001 |
| $\upsilon_{31}$ | Random error term for SBP linear slope | 0.0024 | <0.001 | <0.001 |
| $\varepsilon_{3}$ | SBP measurement error variance | 0.0093 | <0.001 | <0.001 |
| TC Intercept | |  |  |  |
| $\alpha_{40}$ | Population mean TC intercept | 2.9956 | 0.176 | <0.001 |
| $\boldsymbol{\gamma}_{\boldsymbol{40}}$ | Age at baseline coefficient for TC intercept | 0.0456 | 0.003 | <0.001 |
|  | Sex coefficient for TC intercept | 0.0660 | 0.036 | 0.070 |
| $\tau_{40}$ | Association between BMI intercept and TC intercept | 0.4459 | 0.049 | <0.001 |
| $\upsilon_{40}$ | Random error term for TC intercept | 0.8960 | 0.025 | <0.001 |
| TC linear slope | |  |  |  |
| $\alpha_{41}$ | Population mean TC linear slope | 2.1216 | 0.128 | <0.001 |
| $\boldsymbol{\gamma}_{\boldsymbol{41}}$ | Age at baseline coefficient for TC linear slope | -0.0316 | 0.002 | <0.001 |
|  | Sex coefficient for TC linear slope | -0.2677 | 0.026 | <0.001 |
| $\tau_{41}$ | Association between BMI intercept and TC linear slope | -0.4808 | 0.035 | <0.001 |
| $\tau_{42}$ | Association between BMI linear slope and TC linear slope | 0.9802 | 0.108 | <0.001 |
| $\upsilon_{41}$ | Random error term for TC linear slope | 0.1583 | 0.011 | <0.001 |
| $\varepsilon_{4}$ | TC measurement error variance | 0.3426 | 0.006 | <0.001 |
| HDL Intercept | |  |  |  |
| $\alpha_{50}$ | Population mean HDL intercept | 2.4124 | 0.054 | <0.001 |
| $\boldsymbol{\gamma}_{\boldsymbol{50}}$ | Age at baseline coefficient for HDL intercept | 0.0032 | 0.011 | <0.001 |
|  | Sex coefficient for HDL intercept | -0.3710 | 0.001 | <0.001 |
| $\tau_{51}$ | Association between BMI intercept and HDL intercept | -0.3514 | 0.015 | <0.001 |
| $\upsilon_{50}$ | Random error term for HDL intercept | 0.0827 | -0.040 | <0.001 |
| HDL linear slope | |  |  |  |
| $\alpha_{51}$ | Population mean HDL linear slope | 0.1241 | 0.034 | <0.001 |
| $\boldsymbol{\gamma}_{\boldsymbol{51}}$ | Age at baseline coefficient for HDL linear slope | 0.0020 | 0.001 | <0.001 |
|  | Sex coefficient for HDL linear slope | 0.0041 | 0.007 | 0.558 |
| $\boldsymbol{\tau}_{\boldsymbol{51}}$ | Association between BMI intercept and HDL linear slope | -0.0400 | 0.010 | <0.001 |
| $\upsilon_{51}$ | Random error term for HDL linear slope | 0.0090 | 0.001 | <0.001 |
| $\varepsilon_{5}$ | HDL measurement error variance | 0.0333 | 0.001 | <0.001 |

Table 77: Coefficient estimates for latent glycaemic measurement model

|  | Parameter Description | Estimated Mean | Standard error | p-value |
| --- | --- | --- | --- | --- |
| $\mu_{0}$ | FPG intercept | 4.2903 | 0.089 | <0.001 |
| $\theta_{01}$ | Glycaemic factor to FPG | 1 | NA | NA |
| $\theta_{02}$ | Age to FPG | 0.0031 | 0.001 | 0.022 |
| $\theta_{03}$ | Sex to FPG | 0.2129 | 0.021 | <0.001 |
| $\theta_{04}$ | Ethnicity to FPG | 0.0100 | 0.037 | 0.786 |
| $\theta_{05}$ | Family history of diabetes to FPG | 0.1168 | 0.025 | <0.001 |
| $\varepsilon_{0}$ | FPG measurement error variance | 0.1649 | 0.007 | <0.001 |
| $\mu_{1}$ | 2-hr Glucose intercept | 0.5707 | 0.223 | 0.011 |
| $\theta_{11}$ | Glycaemic factor to 2-hr glucose | 2.4384 | 0.078 | <0.001 |
| $\theta_{12}$ | Age to 2-hr glucose | 0.0716 | 0.003 | <0.001 |
| $\theta_{13}$ | Sex to 2-hr glucose | -0.1411 | 0.058 | 0.014 |
| $\theta_{14}$ | Ethnicity to 2-hr glucose | 0.3047 | 0.100 | 0.002 |
| $\theta_{15}$ | Family history of diabetes to 2-hr glucose | 0.3496 | 0.068 | <0.001 |
| $\varepsilon_{1}$ | 2-hr measurement error variance | 2.3679 | 0.054 | <0.001 |
| $\mu_{2}$ | HbA1c intercept | 4.4769 | 0.073 | <0.001 |
| $\theta_{21}$ | Glycaemic factor to HBA1c | 0.5074 | 0.016 | <0.001 |
| $\theta_{22}$ | Age to HBA1c | 0.0101 | 0.001 | <0.001 |
| $\theta_{23}$ | Sex to HBA1c | -0.0457 | 0.001 | <0.001 |
| $\theta_{24}$ | Ethnicity to HBA1c | 0.1854 | 0.030 | <0.001 |
| $\theta_{25}$ | Family history of diabetes to HBA1c | 0.0563 | 0.020 | 0.004 |
| $\varepsilon_{2}$ | HbA1c measurement error variance | 0.1166 | 0.003 | <0.001 |

Table 78: Covariance matrix $\boldsymbol{\Omega}$ for individual random error

|  | $\upsilon_{10}$ | $\upsilon_{11}$ | $\upsilon_{20}$ | $\upsilon_{21}$ | $\upsilon_{22}$ | $\upsilon_{30}$ | $\upsilon_{31}$ | $\upsilon_{40}$ | $\upsilon_{41}$ | $\upsilon_{50}$ | $\upsilon_{51}$ |
| --- | --- | --- | --- | --- | --- | --- | --- | --- | --- | --- | --- |
| $\upsilon_{10}$ | 0.1165 |  |  |  |  |  |  |  |  |  |  |
| $\upsilon_{11}$ | 0.0095 | 0.0131 |  |  |  |  |  |  |  |  |  |
| $\upsilon_{20}$ | <0.0010 | <0.0010 | 0.0851 |  |  |  |  |  |  |  |  |
| $\upsilon_{21}$ | <0.0010 | <0.0010 | 0.0222 | 0.0209 |  |  |  |  |  |  |  |
| $\upsilon_{22}$ | <0.0010 | <0.0010 | <0.0010 | <0.0010 | 0.0107 |  |  |  |  |  |  |
| $\upsilon_{30}$ | <0.0010 | <0.0010 | 0.0080 | <0.0010 | <0.0010 | 0.0085 |  |  |  |  |  |
| $\upsilon_{31}$ | <0.0010 | <0.0010 | <0.0010 | 0.0018 | <0.0010 | <0.0017 | 0.0024 |  |  |  |  |
| $\upsilon_{40}$ | <0.0010 | <0.0010 | 0.0324 | <0.0010 | <0.0010 | 0.0031 | <0.0010 | 0.8960 |  |  |  |
| $\upsilon_{41}$ | <0.0010 | <0.0010 | <0.0010 | -<0.0012 | <0.0010 | <0.0010 | 0.0066 | -0.2229 | 0.1583 |  |  |
| $\upsilon_{50}$ | <0.0010 | <0.0010 | -0.0118 | <0.0010 | <0.0010 | 0.0010 | <0.0010 | 0.0273 | <0.0010 | 0.0827 |  |
| $\upsilon_{51}$ | <0.0010 | <0.0010 | <0.0010 | -0.0059 | <0.0010 | <0.0010 | 0.0020 | <0.0010 | 0.0159 | 0.0061 | 0.0090 |

### HbA1c trajectory in individuals diagnosed with type 2 diabetes

The input parameters for the initial reduction in HbA1c and long term trend in HbA1c following diagnosis, derived from analysis of the UKPDS outcomes model (12), are reported in Table 79 and Table 80 respectively.

Table 79: Estimated change in HbA1c in first year following diabetes diagnosis

|  | Distribution | Parameter 1 | Parameter 2 | Central estimate |
| --- | --- | --- | --- | --- |
| Change in HbA1c Intercept | NORMAL | -2.9465 | 0.0444513 | -2.9465 |
| HbA1c at baseline | NORMAL | 0.5184 | 0.4521958 | 0.5184 |

Table 80: Estimated change in HbA1c following diabetes diagnosis over long term

| Parameter Description | Distribution | Parameter 1 | Parameter 2 | Central estimate |
| --- | --- | --- | --- | --- |
| Longitudinal HbA1c for diabetes intercept | NORMAL | -0.024 | 0.017 | -0.024 |
| Longitudinal HbA1c for diabetes log(time since diagnosis) | NORMAL | 0.144 | 0.009 | 0.144 |
| Longitudinal HbA1c for diabetes Second year | NORMAL | -0.333 | 0.05 | -0.333 |
| Longitudinal HbA1c for diabetes lag HbA1c | NORMAL | 0.759 | 0.004 | 0.759 |
| Longitudinal HbA1c for diabetes HbA1c at diagnosis | NORMAL | 0.085 | 0.004 | 0.0896 |

### Systolic blood pressure and cholesterol trajectory following treatment

The changes in systolic blood pressure and total cholesterol following treatment with anti-hypertensives or statins and statin uptake are reported in Table 81.

Table 81: Treatment effects following treatment

| Parameter Description | Distribution | Parameter 1 | Parameter 2 | Central estimate | Source |
| --- | --- | --- | --- | --- | --- |
| Simvastatin treatment effects | NORMAL | -1.45 | 0.11 | -1.45 | (12) |
| Anti-hypertensive treatment effect | NORMAL | -8.4 | 0.638 | -8.4 | (15) |
| Statin Uptake | UNIFORM | 0.65 | (0.4-0.9) | 0.65 | (13) |

### Metabolic Risk Factor screening

The distribution for the HbA1c threshold at which opportunistic screening for type 2 Diabetes is initiated even if the individual does not have a history of cardiovascular disease, microvascular disease or identified impaired glucose regulation is reported in Table 82.

Table 82: Threshold for HbA1c opportunistic diagnosis

| Parameter Description | Distribution | Parameter 1 | Parameter 2 | Central estimate | Source |
| --- | --- | --- | --- | --- | --- |
| HbA1c at diagnosis | NORMAL | 8.1 | 0.073 | 8.1 | (16) |

## Comorbid Outcomes and Mortality

### Cardiovascular disease

The parameter distributions for men and women based on the QRISK2 model (22) are reported in Table 83.

Table 83: Input parameters of the QRISK2 risk model

| Parameter Description | Distribution | Parameter 1 | Parameter 2 | Central estimate |
| --- | --- | --- | --- | --- |
| QRISK female ethnicity 2 | NORMAL | 0.2163 | 0.0537 | 0.2163 |
| QRISK female ethnicity 3 | NORMAL | 0.6905 | 0.069 | 0.6905 |
| QRISK female ethnicity 4 | NORMAL | 0.3423 | 0.1073 | 0.3423 |
| QRISK female ethnicity 5 | NORMAL | 0.0731 | 0.1071 | 0.0731 |
| QRISK female ethnicity 6 | NORMAL | -0.0989 | 0.0619 | -0.0989 |
| QRISK female ethnicity 7 | NORMAL | -0.2352 | 0.1275 | -0.2352 |
| QRISK female ethnicity 8 | NORMAL | -0.2956 | 0.1721 | -0.2956 |
| QRISK female ethnicity 9 | NORMAL | -0.1010 | 0.0793 | -0.1010 |
| QRISK female smoke 2 | NORMAL | 0.2033 | 0.0152 | 0.2033 |
| QRISK female smoke 3 | NORMAL | 0.48200 | 0.0220 | 0.4820 |
| QRISK female smoke 4 | NORMAL | 0.6126 | 0.0178 | 0.6126 |
| QRISK female smoke 5 | NORMAL | 0.7481 | 0.0194 | 0.7481 |
| QRISK female age 1 | NORMAL | 5.0373 | 1.0065 | 5.0327 |
| QRISK female age 2 | NORMAL | -0.0108 | 0.0022 | -0.0108 |
| QRISK female bmi | NORMAL | 0.4724 | 0.0423 | 0.4724 |
| QRISK female cholesterol | NORMAL | 0.6375 | 0.0143 | 0.6375 |
| QRISK female sbp | NORMAL | 0.0106 | 0.0045 | 0.0106 |
| QRISK female townsend | NORMAL | 0.060 | 0.0068 | 0.060 |
| QRISK female fibrillation | NORMAL | 1.3261 | 0.0310 | 1.3261 |
| QRISK female RA | NORMAL | 0.3626 | 0.0319 | 0.3626 |
| QRISK female Renal | NORMAL | 0.7636 | 0.0639 | 0.7636 |
| QRISK female Hypertension | NORMAL | 0.5421 | 0.0115 | 0.5421 |
| QRISK female diabetes | NORMAL | 0.8940 | 0.0199 | 0.8940 |
| QRISK female family history cvd | NORMAL | 0.5997 | 0.0122 | 0.5997 |
| QRISK female age1 * smoke 1 | NORMAL | 0.1774 | 0.0355 | 0.1774 |
| QRISK female age 1 * smoke 2 | NORMAL | -0.3277 | 0.0655 | -0.3277 |
| QRISK age1 * smoke 3 | NORMAL | -1.1533 | 0.2307 | -1.1533 |
| QRISK female age 1 * smoke 4 | NORMAL | -1.5397 | 0.3079 | -1.5397 |
| QRISK female age 1 * atrial fibrillation | NORMAL | -4.6084 | 0.922 | -4.6084 |
| QRISK female age 1 * renal | NORMAL | -2.6401 | 0.5280 | -2.6401 |
| QRISK female age 1 * hypertension | NORMAL | -2.2480 | 0.4496 | -2.2480 |
| QRISK female age 1 * diabetes | NORMAL | -1.8452 | 0.3690 | -1.8452 |
| QRISK female age 1 * bmi | NORMAL | -3.0851 | 0.6170 | -3.0851 |
| QRISK female age 1 * family history cvd | NORMAL | -0.2481 | 0.0496 | -0.2481 |
| QRISK female age 1 * sbp | NORMAL | -0.0132 | 0.0026 | -0.0132 |
| QRISK female age 1 * town | NORMAL | -0.0369 | 0.0074 | -0.0369 |
| QRISK female age 2 * smoke 1 | NORMAL | -0.0053 | 0..0001 | -0.0053 |
| QRISK female age 2 * smoke 2 | NORMAL | -0.0005 | 0.0001 | -0.0005 |
| QRISK female age 2 * smoke 3 | NORMAL | -0.0105 | 0.0021 | -0.0105 |
| QRISK female age 2 * smoke 4 | NORMAL | -0.0155 | 0.0031 | -0.0155 |
| QRISK female age 2 * fibrillation | NORMAL | -0.0507 | 0.0101 | -0.0507 |
| QRISK female age 2 * renal | NORMAL | 0.0343 | 0.0069 | 0.0343 |
| QRISK female age 2 * hypertension | NORMAL | 0.0258 | 0.0051 | 0.0258 |
| QRISK female age 2 * diabetes | NORMAL | 0.0180 | 0.0036 | 0.0180 |
| QRISK female age 2 * bmi | NORMAL | 0.0345 | 0.0069 | 0.0345 |
| QRISK female age 2 * family history cardiovascular | NORMAL | -0.0062 | 0.0012 | -0.0062 |
| QRISK female age 2 * sbp | NORMAL | -0.000029 | 0.000006 | -0.000029 |
| QRISK female age 2 * townsend | NORMAL | -0.0011 | 0.0002 | -0.0011 |
| QRISK female 1 year survival | CONSTANT | 0.9983 | NA | NA |
| QRISK male ethnicity 2 | NORMAL | 0.3163 | 0.0425 | 0.3163 |
| QRISK male ethnicity 3 | NORMAL | 0.6092 | 0.0547 | 0.6092 |
| QRISK male ethnicity 4 | NORMAL | 0.5958 | 0.0727 | 0.5958 |
| QRISK male ethnicity 5 | NORMAL | 0.1142 | 0.0845 | 0.1142 |
| QRISK male ethnicity 6 | NORMAL | -0.3489 | 0.0641 | -0.3489 |
| QRISK male ethnicity 7 | NORMAL | -0.3604 | 0.1094 | -0.3604 |
| QRISK male ethnicity 8 | NORMAL | -0.2666 | 0.1538 | -0.2666 |
| QRISK male ethnicity 9 | NORMAL | -0.1208 | 0.0734 | -0.1208 |
| QRISK male SMOKE 2 | NORMAL | 0.2033 | 0.0152 | 0.2033 |
| QRISK male SMOKE 3 | NORMAL | 0.4820 | 0.0220 | 0.4820 |
| QRISK male SMOKE 4 | NORMAL | 0.6126 | 0.0178 | 0.6126 |
| QRISK male SMOKE 5 | NORMAL | 0.7481 | 0.0194 | 0.7481 |
| QRISK male age 1 | NORMAL | 47.316 | 9..4630 | 47.316 |
| QRISK male age 2 | NORMAL | -101.236 | 20.247 | -101.236 |
| QRISK male bmi | NORMAL | 0.5425 | 0.0299 | 0.5425 |
| QRISK male cholesterol | NORMAL | 0.14425 | 0.0022 | 0.14425 |
| QRISK male sbp | NORMAL | 0.0081 | 0.0046 | 0.0081 |
| QRISK male townsend | NORMAL | 0.0365 | 0.0048 | 0.0365 |
| QRISK male fibrillation | NORMAL | 0.7547 | 0.1018 | 0.7547 |
| QRISK male RA | NORMAL | 0.3089 | 0.0445 | 0.3089 |
| QRISK male renal | NORMAL | 0.7441 | 0.0702 | 0.7441 |
| QRISK male hypertension | NORMAL | 0.6965 | 0.011 | 0.6965 |
| QRISK male age 1 smoke 1 | NORMAL | -3.8805 | 0.7761 | -3.8805 |
| QRISK male age 1 smoke 2 | NORMAL | -16.703 | 3.3406 | -16.703 |
| QRISK male age 1 smoke 3 | NORMAL | -15.3738 | 3.5291 | -15.3738 |
| QRISK male age 1 smoke 4 | NORMAL | -17.6453 | 3.5291 | -17.6453 |
| QRISK male age 1 fibrillation | NORMAL | -7.0146 | 1.4056 | -7.0282 |
| QRISK male age 1 renal | NORMAL | -17.015 | 3.4029 | -17.015 |
| QRISK male age 1 hypertension | NORMAL | 33.9625 | 6.7925 | 33.9625 |
| QRISK male age 1 diabetes | NORMAL | 12.7886 | 2.5577 | 12.7886 |
| QRISK male age 1 bmi | NORMAL | 3.2680 | 0.6536 | 3.2680 |
| QRISK male age 1 fxcd | NORMAL | -17.9219 | 3.5844 | -17.9219 |
| QRISK male age 1 sbp | NORMAL | -0.1511 | 0.030 | -0.1511 |
| QRISK male age 1 town | NORMAL | -2.5502 | 0.5100 | -2.5502 |
| QRISK male age 2 SMOKE 1 | NORMAL | 7.9709 | 1.5942 | 7.9709 |
| QRISK male age 2 SMOKE 2 | NORMAL | 23.6859 | 4.7372 | 23.6859 |
| QRISK male age 2 SMOKE 3 | NORMAL | 23.1371 | 4.6274 | 23.1371 |
| QRISK male age 2 SMOKE 4 | NORMAL | 26.8674 | 5.3735 | 26.8674 |
| QRISK male age 2 Fibrillation | NORMAL | 14.4518 | 2.8904 | 14.4518 |
| QRISK male age 2 renal | NORMAL | 28.2702 | 5.654 | 28.2702 |
| QRISK male age 2 hypertension | NORMAL | -18.8167 | 3.7633 | -18.8167 |
| QRISK male age 2 diabetes | NORMAL | 0.9630 | 0.1926 | 0.963 |
| QRISK male age 2 bmi | NORMAL | 10.5517 | 2.1103 | 10.5517 |
| QRISK male age 2 FXCD | NORMAL | 26.6047 | 5.3209 | 26.6047 |
| QRISK male age 2 sbp | NORMAL | 0.2911 | 0.0582 | 0.2911 |
| QRISK male age 2 town | NORMAL | 3.007 | 0.6014 | 3.007 |
| QRISK2 male 1 year survival | CONSTANT | 0.997 | NA | NA |

The QRISK2 model was modified to allow a linear relationship between HbA1c and the risk of cardiovascular disease for individuals with Impaired Glucose tolerance and type 2 Diabetes (HbA1c>42 mmol/mol). The parameter distributions for these additional inputs are reported in Table 84.

Table 84: Additional parameters for linear relationship between HbA1c and cardiovascular disease

| Parameter Description | Distribution | Parameter 1 | Parameter 2 | Central estimate | Source |
| --- | --- | --- | --- | --- | --- |
| Female RR of MI due to HbA1c in diabetics | LOGNORMAL | 0.078 | 0.030 | 1.08 | (20) |
| Male RR of MI due to HbA1c in diabetics | LOGNORMAL | 0.108 | 0.023 | 1.11 | (20) |
| RR of stroke due to HbA1c in diabetics | LOGNORMAL | 0.092 | 0.026 | 1.096 | (20) |
| Log(RR) of cvd due to IGR | NORMAL | 0.223 | 0.043 | 1.25 | (26) |

### Congestive Heart Failure

The parameter distributions for congestive heart failure based on the Framingham Heart Study (27) are reported in Table 85.

Table 85: Input parameters for Congestive Heart Failure Risk model for men and women

| Parameter Description | Distribution | Parameter 1 | Parameter 2 | Central estimate |
| --- | --- | --- | --- | --- |
| Male Heart failure baseline hazard | NORMAL | -9.2087 | 0.9209 | -9.2087 |
| Male Heart failure Age | NORMAL | 0.0412 | 0.0278 | 0.0412 |
| Male Heart failure LVH | NORMAL | 0.9026 | 1.0359 | 0.9026 |
| Male Heart failure Heart rate | NORMAL | 0.0166 | 0.0174 | 0.0166 |
| Male Heart failure Systolic blood pressure | NORMAL | 0.00804 | 0.0117 | 0.00804 |
| Male Heart failure CHD | NORMAL | 1.6079 | 0.5336 | 1.6079 |
| Male Heart failure Valve disease | NORMAL | 0.9714 | 0.6557 | 0.9714 |
| Male Heart failure Diabetes | NORMAL | 0.2244 | 0.6682 | 0.2244 |
| Female Heart failure baseline hazard | NORMAL | -10.7988 | 1.0799 | -10.7988 |
| Female Heart failure Age | NORMAL | 0.0503 | 0.0301 | 0.0503 |
| Female Heart failure LVH | NORMAL | 1.3402 | 0.8298 | 1.3402 |
| Female Heart failure Heart rate | NORMAL | 0.0105 | 0.0193 | 0.0105 |
| Female Heart failure Systolic blood pressure | NORMAL | 0.00337 | 0.0109 | 0.00337 |
| Female Heart failure CHD | NORMAL | 1.5549 | 0.5973 | 1.5549 |
| Female Heart failure Valve disease | NORMAL | 1.3929 | 0.6707 | 1.3929 |
| Female Heart failure Diabetes | NORMAL | 1.3857 | 0.7105 | 1.3857 |
| Female Heart failure BMI | NORMAL | 0.0578 | 0.0555 | 0.0578 |
| Female Heart failure Valve disease | NORMAL | -0.986 | 1.4370 | -0.986 |

### Microvascular Complications

The parameter distributions for the risk models for foot ulcer, blindness, renal failure, first amputation and second amputation are reported in Table 86. Parameters for renal failure were based on the UKPDS Outcomes Model 1 (12), whereas parameters for other microvascular complications were based on the UKPDS Outcomes Model 2 (20).

Table 86: Input parameters for microvascular complications

| Parameter Description | Distribution | Parameter 1 | Parameter 2 | Central estimate |
| --- | --- | --- | --- | --- |
| Renal failure baseline hazard | NORMAL | -10.016 | 0.939 | -10.016 |
| Renal failure Weibull shape | NORMAL | 1.865 | 1.4352 | 1.865 |
| Renal failure systolic blood pressure | NORMAL | 0.404 | 0.106 | 0.404 |
| Renal failure blindness | NORMAL | 2.082 | 0.551 | 2.082 |
| Foot ulcer baseline hazard | NORMAL | -11.295 | 1.13 | -11.295 |
| Foot ulcer age at diagnosis | NORMAL | 0.043 | 0.014 | 0.043 |
| Foot ulcer female | NORMAL | -0.962 | 0.255 | -0.962 |
| Foot ulcer BMI | NORMAL | 0.053 | 0.019 | 0.053 |
| Foot ulcer HbA1c | NORMAL | 0.16 | 0.056 | 0.16 |
| Foot ulcer PVD | NORMAL | 0.968 | 0.258 | 0.968 |
| Amputation baseline hazard | NORMAL | -14.844 | 1.205 | -14.844 |
| Amputation age at diagnosis | NORMAL | 0.023 | 0.011 | 0.023 |
| Amputation female | NORMAL | -0.445 | 0.189 | -0.445 |
| Amputation atrial fibrillation | NORMAL | 1.088 | 0.398 | 1.088 |
| Amputation HbA1c | NORMAL | 0.248 | 0.042 | 0.248 |
| Amputation HDL | NORMAL | -0.059 | 0.032 | -0.059 |
| Amputation heart rate | NORMAL | 0.098 | 0.05 | 0.098 |
| Amputation MMALB | NORMAL | 0.602 | 0.18 | 0.602 |
| Amputation peripheral vascular disease | NORMAL | 1.01 | 0.189 | 1.01 |
| Amputation white blood count | NORMAL | 0.04 | 0.017 | 0.04 |
| Amputation Stroke | NORMAL | 1.299 | 0.245 | 1.299 |
| Amputation shape | NORMAL | 2.067 | 0.193 | 2.067 |
| Amputation with Ulcer lambda | NORMAL | -0.881 | 0139 | -0.881 |
| Amputation with Ulcer age at diagnosis | NORMAL | -0.065 | 0.027 | -0.065 |
| Amputation with Ulcer PVD | NORMAL | 1.769 | 0.449 | 1.769 |
| Second Amputation baseline hazard | NORMAL | -3.455 | 0.565 | -3.455 |
| Second Amputation HbA1c | NORMAL | 0.127 | 0.06 | 0.127 |
| Blindness baseline hazard | NORMAL | -10.6774 | 0.759 | -10.6774 |
| Blindness age at diagnosis | NORMAL | 0.047 | 0.009 | 0.047 |
| Blindness HbA1c | NORMAL | 0.171 | 0.032 | 0.171 |
| Blindness heart rate | NORMAL | 0.08 | 0.039 | 0.08 |
| Blindness systolic blood pressure | NORMAL | 0.068 | 0.032 | 0.068 |
| Blindness white blood cells | NORMAL | 0.052 | 0.019 | 0.052 |
| Blindness CHF | NORMAL | 0.841 | 0.287 | 0.841 |
| Blindness IHD | NORMAL | 0.61 | 0.208 | 0.61 |

### Cancer

The parameter distributions for the incidence and hazard ratios for breast cancer and colorectal cancer are reported in Table 87.

Table 87: Input parameters for breast cancer and colorectal cancer risk models

| Parameter Description | Distribution | Parameter 1 | Parameter 2 | Central estimate | Source |
| --- | --- | --- | --- | --- | --- |
| Colorectal cancer men | NORMAL | 0.0011 | 0.0001 | 0.0011 | (34) |
| Colorectal cancer women | NORMAL | 0.0005 | 0.0000 | 0.0005 | (34) |
| Breast cancer pre-menopause | NORMAL | 0.0010 | 0.0001 | 0.0010 | (32) |
| Breast cancer post-menopause | NORMAL | 0.0028 | 0.0002 | 0.0028 | (32) |
| Colorectal cancer BMI relative risk for men | LOGNORMAL | 0.1906 | 0.0111 | 1.21 | (33) |
| Colorectal cancer BMI relative risk for women | LOGNORMAL | 0.0392 | 0.0151 | 1.04 | (33) |
| Breast cancer BMI relative risk for pre-menopause | LOGNORMAL | -0.1165 | 0.0251 | 0.89 | (33) |
| Breast cancer BMI relative risk for post-menopause | LOGNORMAL | 0.0862 | 0.0205 | 1.09 | (33) |

The parameter distributions for breast and colorectal cancer mortality are reported in Table 88.

Table 88: Input parameters for breast cancer and colorectal cancer mortality (38)

| Parameter Description | Distribution | Parameter 1 | Parameter 2 | Central estimate |
| --- | --- | --- | --- | --- |
| Breast cancer 5 year survival | BETA | 439.69 | 2354.44 | 0.157 |
| Colorectal cancer 5 year survival | BETA | 1457.56 | 1806.35 | 0.447 |

### Osteoarthritis

The parameter distributions for the incidence and hazard ratios for osteoarthritis are reported below.

Table 89: Input parameters for the osteoarthritis risk model (5)

| Parameter Description | Distribution | Parameter 1 | Parameter 2 | Central estimate |
| --- | --- | --- | --- | --- |
| Osteoarthritis incidence | NORMAL | 0.0053 | 0.0000004 | 0.0053 |
| Osteoarthritis RR of diabetes | LOGNORMAL | 0.723 | 0.317 | 2.06 |
| Osteoarthritis RR of BMI | LOGNORMAL | 0.073 | 0.026 | 1.076 |

### Depression

The parameter distributions for the incidence and hazard ratios for depression are reported below.

Table 90: Input parameters for the depression risk model

| Parameter Description | Distribution | Parameter 1 | Parameter 2 | Central estimate | Source |
| --- | --- | --- | --- | --- | --- |
| Odds of depression | BETA | 336 | 8803 | 0.0397 | (36) |
| Odds ratio for diabetes | LOGNORMAL | 0.4187 | 0.1483 | 1.52 | (36) |
| Odds ratio for stroke | LOGNORMAL | 1.8406 | 0.5826 | 6.3 | (37) |

### Mortality

The other cause mortality rates by age were assumed constant in the probabilistic sensitivity analysis (39). The parameter distribution for the hazard ratio for other cause mortality with diabetes is reported below.

Table 91: Input parameters for mortality hazard ratio for diabetes (37)

| Parameter Description | Distribution | Parameter 1 | Parameter 2 | Central estimate |
| --- | --- | --- | --- | --- |
| Mortality hazard ratio for diabetes | LOGNORMAL | 0.588 | 0.186 | 1.80 |

## Utilities

The parameter distributions used to estimate health state utilities in the model are reported below.

Table 92: Utility input parameters

| Parameter Description | Distribution | Parameter 1 | Parameter 2 | Central estimate | Source |
| --- | --- | --- | --- | --- | --- |
| Renal/ulcer baseline utility | NORMAL | 0.689 | 0.014 | 0.689 | (70) |
| Renal dialysis | NORMAL | -0.078 | 0.026 | -0.078 | (70) |
| Foot ulcer | NORMAL | -0.099 | 0.013 | -0.099 | (70) |
| Amputation/heart failure baseline utility | NORMAL | 0.807 | 0.005 | 0.807 | (20) |
| Heart failure | NORMAL | -0.101 | 0.032 | -0.101 | (20) |
| Amputation | NORMAL | -0.172 | 0.045 | -0.172 | (20) |
| Stable angina multiplicative factor decrement | NORMAL | 0.801 | 0.038 | 0.801 | (14) |
| Unstable angina multiplicative factor decrement | NORMAL | 0.77 | 0.038 | 0.77 | (14) |
| MI multiplicative factor decrement | NORMAL | 0.76 | 0.018 | 0.76 | (14) |
| Stroke multiplicative factor decrement | NORMAL | 0.629 | 0.04 | 0.629 | (14) |
| Cancer baseline utility | NORMAL | 0.8 | 0.0026 | 0.8 | (73) |
| Cancer decrement | NORMAL | -0.06 | 0.008 | -0.06 | (73) |
| Osteoarthritis utility | NORMAL | 0.69 | 0.069 | 0.69 | (74) |
| Depression baseline utility | NORMAL | 0.48 | 0.048 | 0.48 | (76) |
| Depression remitters | NORMAL | 0.31 | 0.031 | 0.31 | (76) |
| Depression responders | NORMAL | 0.20 | 0.020 | 0.20 | (76) |
| Depression non-responders | NORMAL | 0.070 | 0.007 | 0.070 | (76) |
| Depression drop-outs | NORMAL | 0.050 | 0.005 | 0.050 | (76) |
| Age utility decrement | NORMAL | -0.004 | 0.0001 | -0.004 | (14) |

## Unit Health Care Costs

| Parameter Description | Distribution | Parameter 1 | Parameter 2 | Central estimate | Source |
| --- | --- | --- | --- | --- | --- |
| Cost of insulin | GAMMA | 3.194 | 391.85 | 1251.5 | (47) |
| Cost of anti-hypertensives | GAMMA | 100 | 1.83 | 183.01 | (48) |
| Cost of GP appointment | GAMMA | 100 | 0.43 | 43 | (62) |
| Nurse appointment (Advanced) | GAMMA | 100 | 0.25 | 25 | (62) |
| Health care assistant appointment | GAMMA | 100 | 0.0417 | 4.17 | (62) |
| Eye screening | GAMMA | 15.366 | 1.478 | 22.709 | (45) |
| HbA1c test | GAMMA | 100 | 0.03 | 3 | (42) |
| Lipids test | GAMMA | 100 | 0.03 | 1 | (42) |
| LfT test | GAMMA | 100 | 0.03 | 1 | (42) |
| B12 test | GAMMA | 100 | 0.03 | 1 | (42) |
| Urine test | GAMMA | 100 | 0.03 | 1 | (42) |
| Nicotine replacement therapy | GAMMA | 100 | 1.02 | 102 | (62) |
| HbA1c diagnosis screening | GAMMA | 100 | 0.14 | 14 | (42) |
| Unstable Angina hospital admission | GAMMA | 311.79 | 3 | 1191.4 | (13) |
| Revascularisation in hospital | GAMMA | 300 | 17 | 5638.6 | (13) |
| MI Hospital admission | GAMMA | 248.48 | 5 | 1452.3 | (13) |
| First Outpatient appointment | GAMMA | 100 | 1 | 154.45 | (13) |
| Subsequent outpatient appointments | GAMMA | 75 | 1 | 102.8 | (13) |
| Fatal CHD | GAMMA | 300 | 2 | 665.5 | (47) |
| Fatal Stroke | GAMMA | 280 | 13 | 4149.5 | (46) |
| First year stroke cost | GAMMA | 350 | 23 | 9075 | (46) |
| Subsequent year stroke cost | GAMMA | 100 | 26 | 2579 | (13) |
| Glytrin Spray | CONSTANT | 11.92 | NA | NA | (13) |
| Isosorbide mononitrate | CONSTANT | 12.79 | NA | NA | (13) |
| Verapamil | CONSTANT | 47.79 | NA | NA | (13) |
| Atenolol | CONSTANT | 34.42 | NA | NA | (13) |
| Aspirin | CONSTANT | 7.57 | NA | NA | (13) |
| Ramipril | CONSTANT | 85.47 | NA | NA | (13) |
| ARB | CONSTANT | 239.35 | NA | NA | (13) |
| Clopidogrel | CONSTANT | 523.92 | NA | NA | (13) |
| Congestive Heart Failure | GAMMA | 67 | 43 | 2921 | (98) |
| Blindness year 1 | GAMMA | 24 | 47 | 1147 | (98) |
| Blindness subsequent years | GAMMA | 36 | 10 | 370 | (98) |
| Amputation year 1 | GAMMA | 27 | 405 | 11125 | (98) |
| Amputation subsequent years | GAMMA | 16 | 24 | 395 | (98) |
| Renal Haemodialysis | GAMMA | 100 | 397 | 39736 | (53) |
| Renal Automated Peritoneal dialysis | GAMMA | 100 | 257 | 25720 | (53) |
| Renal Ambulatory peritoneal dialysis | GAMMA | 100 | 187 | 18657 | (53) |
| Renal transplant | GAMMA | 100 | 224 | 22359 | (55) |
| Immunosuppressants | GAMMA | 100 | 66 | 6576 | (55) |
| Foot ulcer not infected | GAMMA | 100 | 1.59 | 158.53 | (56) |
| Foot ulcer with cellulitis | GAMMA | 100 | 4.19 | 419 | (56) |
| Foot ulcer with osteomyelitis | GAMMA | 100 | 7.76 | 776 | (56) |
| Breast Cancer | GAMMA | 100 | 130.58 | 13058 | (58) |
| Colorectal cancer Dukes A | GAMMA | 100 | 95.36 | 9536 | (59) |
| Colorectal cancer Dukes B | GAMMA | 100 | 163.63 | 163.63 | (59) |
| Colorectal cancer Dukes C | GAMMA | 100 | 250.90 | 25090 | (59) |
| Colorectal cancer Dukes D | GAMMA | 100 | 157.11 | 15711 | (59) |
| Osteoarthritis | GAMMA | 100 | 9.09 | 909 | (60) |
| Depression – Practice nurse surgery | GAMMA | 100 | 0.09 | 8.83 | (61) |
| Depression – Practice nurse home | GAMMA | 100 | 0.27 | 26.50 | (61) |
| Depression – Practice nurse telephone | GAMMA | 100 | 0.09 | 8.83 | (61) |
| Depression – Health visitor | GAMMA | 100 | 0.36 | 35.50 | (61) |
| Depression – District nurse | GAMMA | 100 | 0.25 | 24.50 | (61) |
| Depression – Other nurse | GAMMA | 100 | 0.09 | 8.83 | (61) |
| Depression – HCA phlebotomist | GAMMA | 100 | 0.04 | 4.17 | (61) |
| Depression – Other primary care | GAMMA | 100 | 0.25 | 25.00 | (61) |
| Depression – Out of Hours | GAMMA | 100 | 0.25 | 25.39 | (61) |
| Depression – NHS Direct | GAMMA | 100 | 0.24 | 23.90 | (61) |
| Depression – Walk-in Centre | GAMMA | 100 | 0.37 | 36.70 | (61) |
| Depression – Prescribed medicines | GAMMA | 100 | 0.09 | 9.09 | (61) |
| Depression – Secondary Care | GAMMA | 100 | 1.09 | 109.00 | (61) |

## Intervention Effects

|  | Distribution | Parameter 1 | Parameter 2 | Central Estimate | Source |
| --- | --- | --- | --- | --- | --- |
| Soft drinks tax – BMI 16-29 | NORMAL | -0.23 | 0.020 | -0.23 | (83) |
| Soft drinks tax – BMI 30-49 | NORMAL | -0.05 | 0.010 | -0.05 | (83) |
| Soft drinks tax – BMI 50+ | NORMAL | 0 | 0.010 | 0 | (83) |
| Retail Policy – HbA1c | NORMAL | -0.01 | 0.002 | -0.01 | (84) |
| Retail Policy – Systolic Blood Pressure | NORMAL | -0.46 | 0.086 | -0.46 | (84) |
| Workplace – HbA1c (Vitamin C) | NORMAL | -0.06 | 0.014 | -0.06 | (85;86) |
| Workplace – Systolic Blood Pressure | NORMAL | -2.86 | 0.531 | -2.86 | (85;86) |
| Workplace – HbA1c (Vitamin C) | NORMAL | -0.02 | 0.003 | -0.02 | (85;86) |
| Education – BMI Male | NORMAL | -1.29 | 0.258 | -1.29 | (91) |
| Education – BMI Female | NORMAL | -1.04 | 0.208 | -1.04 | (92) |
| Education – HbA1c | NORMAL | -0.01 | 0.002 | -0.01 | (85;86) |
| Education – Systolic blood pressure | NORMAL | -0.41 | 0.076 | -0.41 | (85;86) |
| IGR lifestyle – BMI | NORMAL | -0.96 | 0.157 | -0.96 | (94) |
| IGR lifestyle – HbA1c | NORMAL | -0.13 | 0.043 | -0.13 | (94) |
| IGR lifestyle – Systolic blood pressure | NORMAL | -4.3 | 0.923 | -4.3 | (94) |
| IGR lifestyle - Cholesterol | NORMAL | -0.18 | 0.028 | -0.18 | (94) |

# One-Way Sensitivity Analyses

A series of one-way sensitivity analyses were carried out in order to test the strength of certain assumptions that we had made. These were of two types. Firstly, some of the assumptions based upon particularly weak data relating to the interventions were tested. In addition, we carried out sensitivity analyses related to some of the other parameters and assumptions used in the model.

## Sensitivity Analysis around the Interventions

Much of the data for the five interventions is based upon assumptions and extrapolations from multiple data sources rather than from long term randomised controlled trials collecting data on multiple metabolic risk factor endpoints. We decided to test the sensitivity of the results to modifying those parameters that were based upon particularly weak data. They focus on three main areas of uncertainty; duration of intervention effect, intervention uptake and intervention efficacy. The one-way sensitivity analyses around the interventions are summarised in Table 93.

Table 93: One-way sensitivity analyses used to test the strength of parameters

| Parameter Affected | Modification | Justification | Interventions Affected |
| --- | --- | --- | --- |
| Duration of Intervention Effect | Make effects last for duration of lifetime | Intervention is continuous due to policy change. | A  B |
|  | Increase duration of effects - diminish over ten years | Original five year duration based only on estimation | C  D  E |
|  | Reduce duration of effects - diminish over two years | Original five year duration based only on estimation | C  D  E |
| Intervention Uptake | Switch uptake rates between Intervention D and Intervention E. | Would be useful to see how important uptake rates are on intervention success | D  E |
|  | Reduce number of people affected by intervention B to 50% of upper Townsend quintile. | Unlikely that everyone in deprived areas is lacking good retail provision. | B |
| Intervention Efficacy | Increase efficacy of intervention C by estimating extra effects on BMI and HbA1c due to reducing fried food/sweet puddings. | Increasing the competitiveness of the workplace intervention. | C |
|  | Remove the effects of vitamin C and fat intake on HbA1c levels | Cross-sectional studies used to provide data. Only show correlation, not causation. | B  C  D |

### Duration of Intervention Effect

For the majority of interventions no data exists for the duration of intervention effect, and so for all interventions it was estimated to be maximal in year one, then diminish to zero (compared with basecase) over the following five years. A fixed duration of effect across all interventions was chosen to allow comparisons of the interventions independent upon the duration of response. This assumption is particularly unrealistic for interventions A and B, as both a soft drinks tax and a retail policy resulting in better access to fruit and vegetables would be likely to have a persistent effect in changing habits and behaviours. For interventions C, D and E, we decided to test the strength of the original assumption by either increasing the duration of effect to 10 years or reducing it to two years.

### Intervention Uptake

The uptake of interventions is likely to have a large implication for their efficacy in reducing disease prevalence on a population-wide basis. Interventions D (community weight loss intervention) and E (screening and intensive intervention in high risk individuals) have very different uptake rates; only 11.4% of the eligible population choose to take up intervention D, whilst 42% choose to be screened and undergo intervention E. We decided to switch the uptake rates for interventions D and E to see how this affected their relative cost-effectiveness.

We also thought that there was some uncertainty around the question of how many people currently have poor access to fresh fruit and vegetables, and therefore would benefit from the retail intervention (B). There is very little data on how many people are actually living in ‘food deserts’, but it would be reasonable to assume that at least some of the most deprived people do currently have good local retail access and therefore that our initial assumptions would overestimate the numbers who would benefit. To test the sensitivity of the results to this assumption, we decided to randomly select 50% of the most deprived quintile of the population to receive the intervention.

### Intervention Efficacy

Although efficacy is based on good quality data for many of the interventions, there are still a few areas for uncertainty. For the workplace intervention (C), the data was particularly poor as no information was given about the magnitude of changes in consumption of fruit, sweet puddings and fried food (99). The latter two are particularly difficult to estimate due to a lack of data about potential food substitution, or what in particular is represented by a sweet pudding or fried food, and therefore they were not included in the original analysis. We thought it would be interesting to try to maximise the potential health benefits of intervention C by estimating (in a fairly arbitrary way) the effect of reductions in fried food and sweet puddings on BMI and HbA1c (via fat intake) as reported by 5.3% and 5.5% of individuals respectively. A reduction in fried food was considered to be a replacement of one Big Mac meal with a healthy home cooked meal each week, resulting in a reduction of 1000 calories and 70g of fat per week. A reduction in sweet puddings was considered to be a replacement of one sponge pudding with a low fat yoghurt each week, resulting in a reduction of 500 calories and 27g of fat per week.

Another area for uncertainty regarding intervention efficacy is surrounding two pieces of cross-sectional data that were used to derive reductions in HbA1c levels dependent upon vitamin C intake and fat intake (105;109). The data indicates that there is a correlation between HbA1c levels and vitamin C/fat intake, but this does not imply that there is a causative link. Given the lack of better quality data, values from these sources were used to inform the analysis. We decided that it was important to test how sensitive the cost-effectiveness of interventions B, C and D were to the removal of parameters based upon these correlations.

In addition we ran one way sensitivity analyses for all interventions using their 95% confidence intervals for treatment effect. We also looked at the impact of variation in intervention cost by 20%.

## Sensitivity Analysis around other Parameters

The list of other one-way sensitivity analyses carried out is presented in Table 94.

Table 94: List of sensitivity analyses

| **Description of Sensitivity Analysis** |
| --- |
| Discount rate 0% |
| Discount rate 3.5% |
| Non-intervention costs at 2.5^th^ CI |
| Non-intervention costs at 97.5^th^ CI |
| Cardiovascular costs at 2.5^th^ CI |
| Cardiovascular costs at 97.5^th^ CI |
| Diabetes costs at 2.5^th^ CI |
| Diabetes costs at 97.5^th^ CI |
| Microvascular costs at 2.5^th^ CI |
| Microvascular costs at 97.5^th^ CI |
| All utility decrements at 2.5^th^ CI |
| All utility decrements 97.5^th^ CI |
| Cardiovascular utility decrements at 2.5^th^ CI |
| Cardiovascular utility decrements at 97.5^th^ CI |
| Microvascular utility decrements at 2.5^th^ CI |
| Microvascular utility decrements at 97.5^th^ CI |
| BMI utility decrements at 2.5^th^ CI |
| BMI utility decrements at 97.5^th^ CI |
| Statin uptake 50% |
| Statin uptake 80% |
| QRISK IGR hazard ratio 2.5^th^ CI |
| QRISK IGR hazard ratio 97.5^th^ CI |
| No BMI effect on cancer incidence |
| No BMI or diabetes effect on osteoarthritis incidence |
| No diabetes or stroke effect on depression incidence |
| No diabetes effect on mortality |
| Low cardiovascular incidence |
| High cardiovascular incidence |
| Low diabetes diagnosis threshold |
| High diabetes diagnosis threshold |
| Low congestive heart failure incidence |
| High congestive heart failure incidence |

# Model Limitations and Further Research

## Model Limitations

**Limited baseline sample data:** The model is based on data from 8038 individuals from the HSE 2011. A large proportion of individuals were missing answers for at least one of the variables required for input into the model, so missing data had to be assumed or imputed. Imputation relied on an assumption that non-response was arbitrary for each variable, which may not be correct. For other variables, assumption of negative responses may underestimate the true numbers of individuals affected. Although the HSE should be broadly representative of the UK population, the relatively small numbers and the necessity to rely only on data from individuals who were willing to respond, means that data may be biased or skewed for some variables. The use of a UK adult population also means that the model may not be appropriate for modelling diabetes in other countries or in children.

**Whitehall trajectories:** Use of a quadratic form is beneficial because glycaemia increases at an increasing rate as observed in other studies (99). This provides a better description of the implications of not screening for diabetes, because unscreened individuals will not be detected until their HbA1c levels are much higher. A linear slope would describe a much more shallow progression of HbA1c before diabetes is detected. However, a disadvantage of this functional form is that all individuals are simulated with the same timescale, therefore progression is slow in the short term but increases for all individuals as time increases.

**Poor quality intervention data:** Many of the interventions are based upon poor quality data due to the lack of good information about the effect on metabolic factors, general lack of detail as to intervention efficacy and short follow-up times. In particular, the evidence relating fat intake or plasma vitamin C to HbA1c levels was based on two cross-sectional studies, which means the effect is only correlative and not necessarily causative. We have also had to make many assumptions in implementing interventions. The retail policy was assumed to affect all individuals living in deprived areas, but many of these people will now have good access to fruit and vegetable provision. The workplace intervention was assumed to affect 20% of the working population, although we have no data on how many individuals would really be affected. The workplace intervention data was also very vague in terms of quantifying diet changes and we were forced to assume its effects on fruit and vegetable consumption and milk switching. We also were unable to find studies relating intake of certain foods to effects on metabolic factors and as a consequence we have potentially underestimated the efficacy of interventions. For example, we could not find any data linking sugar intake to HbA1c levels, or fruit and vegetable intake to cholesterol levels.

**Model complexity:** The complexity of the simulation is necessary to encompass the multiple factors impacting on type 2 diabetes and the multiple outcomes of hyper-glycaemia. However, this complexity also means that the model is difficult to understand and it is very difficult to ensure removal of all potential errors. This means that we cannot guarantee that the model is free of errors that could potentially have effects on costs, QALYs and resulting cost-effectiveness of interventions.

## Further Research

**Improvements to the Whitehall model for metabolic risk trajectories:** The Whitehall II analysis assumed that all participants were observed at equal time intervals between phases of the study. This was necessary to be able to implement the analysis in MPlus software, in which the data needed to be specified in wide format. Analyses by age group were investigated but could not be completed because of the low proportion of observations between age groups. The variation in time intervals between phases was not large and was not expected to impact substantially on the results of these analyses. Nonetheless we would recommend that further research explores an alternative specification of the model in which time is a continuous variable. This would also allow a more flexible specification of the trajectory of HbA1c and may avoid assuming a quadratic functional form.

The effect of changes in BMI on changes in glycaemia has been shown to be small in the Whitehall II analysis. It is likely that changes in physical activity and diet will have additional effects on changes in glycaemia, independent of their indirect effects on BMI. We have incorporated the effects of diet into the model using other data sources. However, further research should explore whether these factors could be incorporated into the Whitehall II analysis to allow them to be causally related to all metabolic risk factors in a longitudinal analysis.

**Incorporate a behavioural intervention to increase physical activity:** We identified a systematic review and meta-analysis of randomised controlled trials for behavioural interventions targeting physical activity and exercise in type 2 diabetics (100). The study found that a range of targeted behavioural interventions were successful in significantly increasing physical activity in diagnosed diabetics, leading to corresponding improvements in BMI and HbA1c. The mean reduction in BMI was 1.05 kg/m^2^ and the mean reduction in HbA1c was 0.32% for follow-up times ranging from 1 month to 2 years.

These values could be implemented directly into the model in a similar way to those used for the translational diabetes prevention programme (Section 14.6). As for the other interventions, the effect could be assumed to be maximal in the first year and diminish linearly over the next 5 years, although sensitivity analysis should test the possibility of a sustained reduction over a longer period of time, as this was suggested from the small number of trials with 2 year follow-up. Intervention costs were not calculated in the study and would have to be estimated directly from clinical trial data or other sources. Cost and efficacy can vary widely depending upon the nature of the intervention and the training given to interventionists, so ideally sensitivity analysis would be used to determine cost-effectiveness given a range of intervention costs and associated improvements in BMI and HbA1c.

**Incorporate fibre intake into dietary interventions:** One of our stakeholders identified a meta-analysis linking intake of dietary fibre to HbA1c levels (101). This could be incorporated into the model as part of a dietary intervention in which participants are encouraged to eat more fibre. None of the studies used in the meta-analysis involved participants eating more fruit and vegetables; rather the focus was on eating high fibre bread and cereals, or on adding fibre such as Guar gum directly to the normal diet. In theory, the amount of fibre in an average portion of fruit and vegetables could be calculated and the resulting effect on HbA1c determined. However, given that the effect of fruit and vegetable intake on HbA1c is already incorporated within the model via plasma vitamin C levels, and it is unclear whether effects of fibre and vitamin C on HbA1c are independent, further research is required before this option is taken.

**Investigate subgroup-specific differences in intervention uptake, efficacy or duration:** Current analyses of high risk subgroups assume that interventions have the same uptake, the same efficacy in reducing metabolic trajectories and the same duration of action in different population subgroups. This is unlikely to be true as it is known for example, that individuals from deprived areas are less likely to take up screening opportunities. Further research is required to investigate the current evidence base on subgroup specific intervention effects and use the resulting data to tailor intervention effect accordingly.

Reference List

(1) Watson P, Preston L, Squires H, Chilcott J, Brennan A. Modelling the Economics of Type 2 Diabetes Mellitus Prevention: A Literature Review of Methods. Appl Health Econ Health Policy 2014;12(3):239-53.

(2) Squires H. A methodological framework for developing the structure of Public Health economic models. White Rose ethesis online 2014Available from: URL: <http://etheses.whiterose.ac.uk/5316/>

(3) National Institute for Health and Care Excellence. PH35: Preventing type 2 diabetes: population and community-level interventions. National Institute for Health and Care Excellence 2011NICE public health guidance 35Available from: URL: https://[www.nice.org.uk/guidance/ph35](http://www.nice.org.uk/guidance/ph35)

(4) National Institute for Health and Care Excellence. PH38 Preventing type 2 diabetes - risk identification and interventions for individuals at high risk: guidance. National Institute for Health and Care Excellence 2012NICE public health guidance 38Available from: URL: <http://guidance.nice.org.uk/PH38/Guidance/pdf/English>

(5) Schett G, Kleyer A, Perricone C, Sahinbegovic E, Iagnocco A, Zwerina J, et al. Diabetes is an independent predictor for severe osteoarthritis: results from a longitudinal cohort study. Diabetes Care 2013 Feb;36(2):403-9.

(6) Gillett M, Royle P, Snaith A, Scotland G, Poobalan A, Imamura M, et al. Non-pharmacological interventions to reduce the risk of diabetes in people with impaired glucose regulation: a systematic review and economic evaluation. Health Technol Assess 2012 Aug;16(33):1-iv.

(7) Panel on Food, Nutrition, Physical Activity and the Prevention of Cancer. World Cancer Research Fund (WCRF) 2014Available from: URL: [www.dietandcancerreport.org/cancer_resource_center/downloads/Second_Expert_Report_full.pdf](http://www.dietandcancerreport.org/cancer_resource_center/downloads/Second_Expert_Report_full.pdf)

(8) NatCen Social Research. Health Survey for England. University College London Department of Epidemiology and Public Health 2011Available from: URL: <http://www.esds.ac.uk/findingData/hseTitles.asp>

(9) Marmot M, Brunner E. Cohort Profile: the Whitehall II study. Int J Epidemiol 2005 Apr;34(2):251-6.

(10) Green MA, Li J, Relton C, Strong M, Kearns B, Wu M, et al. Cohort Profile: The Yorkshire Health Study. Int J Epidemiol 2014 Jul 9;dyu121.

(11) Colagiuri S, Cull CA, Holman RR. Are lower fasting plasma glucose levels at diagnosis of type 2 diabetes associated with improved outcomes?: U.K. prospective diabetes study 61. Diabetes Care 2002 Aug;25(8):1410-7.

(12) Clarke PM, Gray AM, Briggs A, Farmer AJ, Fenn P, Stevens RJ, et al. A model to estimate the lifetime health outcomes of patients with type 2 diabetes: the United Kingdom Prospective Diabetes Study (UKPDS) Outcomes Model (UKPDS no. 68). Diabetologia 2004 Oct;47(10):1747-59.

(13) Ara R, Pandor A, Stevens J, Rees A, Rafia R. Early high-dose lipid-lowering therapy to avoid cardiac events: a systematic review and economic evaluation. Health Technol Assess 2009 Jul;13(34):1-118.

(14) Ward S, Lloyd JM, Pandor A, Holmes M, Ara R, Ryan A, et al. A systematic review and economic evaluation of statins for the prevention of coronary events. Health Technol Assess 2007 Apr;11(14):1-iv.

(15) National Institute for Health and Care Excellence. Hypertension: Clinical management of primary hypertension in adults. 2011. Report No.: CG 127.

(16) Wald DS, Law M, Morris JK, Bestwick JP, Wald NJ. Combination therapy versus monotherapy in reducing blood pressure: meta-analysis on 11,000 participants from 42 trials. Am J Med 2009 Mar;122(3):290-300.

(17) Davies MJ, Heller S, Skinner TC, Campbell MJ, Carey ME, Cradock S, et al. Effectiveness of the diabetes education and self management for ongoing and newly diagnosed (DESMOND) programme for people with newly diagnosed type 2 diabetes: cluster randomised controlled trial. BMJ 2008 Mar 1;336(7642):491-5.

(18) National Institute of Health and Care Excellence. Statins for the prevention of cardiovascular events in patients at increased risk of developing cardiovascular disease or those with established cardiovascular disease. National Institute for Health and Care Excellence; 2006. Report No.: Technology appraisals, TA94.

(19) CG127 Hypertension: costing template. National Institute for Care and Clinical Excellence 2011Available from: URL: <http://guidance.nice.org.uk/CG127/CostingTemplate/xls/English>

(20) Hayes AJ, Leal J, Gray AM, Holman RR, Clarke PM. UKPDS outcomes model 2: a new version of a model to simulate lifetime health outcomes of patients with type 2 diabetes mellitus using data from the 30 year United Kingdom Prospective Diabetes Study: UKPDS 82. Diabetologia 2013 Sep;56(9):1925-33.

(21) D'Agostino RB, Sr., Vasan RS, Pencina MJ, Wolf PA, Cobain M, Massaro JM, et al. General cardiovascular risk profile for use in primary care: the Framingham Heart Study. Circulation 2008 Feb 12;117(6):743-53.

(22) Hippisley-Cox J, Coupland C, Vinogradova Y, Robson J, Minhas R, Sheikh A, et al. Predicting cardiovascular risk in England and Wales: prospective derivation and validation of QRISK2. BMJ 2008 Jun 28;336(7659):1475-82.

(23) McEwan P, Bennett H, Ward T, Bergenheim K. Refitting of the UKPDS 68 risk equations to contemporary routine clinical practice data in the UK. Pharmacoeconomics 2015 Feb;33(2):149-61.

(24) ClinRisk. QResearch 2013Available from: URL: <http://www.qrisk.org/>

(25) Hippisley-Cox J, Coupland C, Robson J, Brindle P. Derivation, validation, and evaluation of a new QRISK model to estimate lifetime risk of cardiovascular disease: cohort study using QResearch database. BMJ 2010 Dec 9;341:c6624. doi: 10.1136/bmj.c6624.:c6624.

(26) Khaw KT, Wareham N, Luben R, Bingham S, Oakes S, Welch A, et al. Glycated haemoglobin, diabetes, and mortality in men in Norfolk cohort of european prospective investigation of cancer and nutrition (EPIC-Norfolk). BMJ 2001 Jan 6;322(7277):15-8.

(27) Kannel WB, D'Agostino RB, Silbershatz H, Belanger AJ, Wilson PW, Levy D. Profile for estimating risk of heart failure. Arch Intern Med 1999 Jun 14;159(11):1197-204.

(28) Kaffashian S, Dugravot A, Brunner EJ, Sabia S, Ankri J, Kivimaki M, et al. Midlife stroke risk and cognitive decline: a 10-year follow-up of the Whitehall II cohort study. Alzheimers Dement 2013 Sep;9(5):572-9.

(29) Johansen NB, Vistisen D, Brunner EJ, Tabak AG, Shipley MJ, Wilkinson IB, et al. Determinants of aortic stiffness: 16-year follow-up of the Whitehall II study. PLoS One 2012;7(5):e37165.

(30) Dadvand P, Rankin J, Shirley MD, Rushton S, Pless-Mulloli T. Descriptive epidemiology of congenital heart disease in Northern England. Paediatr Perinat Epidemiol 2009 Jan;23(1):58-65.

(31) Davies M, Hobbs F, Davis R, Kenkre J, Roalfe AK, Hare R, et al. Prevalence of left-ventricular systolic dysfunction and heart failure in the Echocardiographic Heart of England Screening study: a population based study. Lancet 2001 Aug 11;358(9280):439-44.

(32) Lahmann PH, Hoffmann K, Allen N, van Gils CH, Khaw KT, Tehard B, et al. Body size and breast cancer risk: findings from the European Prospective Investigation into Cancer And Nutrition (EPIC). Int J Cancer 2004 Sep;111(5):762-71.

(33) Renehan AG, Tyson M, Egger M, Heller RF, Zwahlen M. Body-mass index and incidence of cancer: a systematic review and meta-analysis of prospective observational studies. Lancet 2008 Feb 16;371(9612):569-78.

(34) Pischon T, Lahmann PH, Boeing H, Friedenreich C, Norat T, Tjonneland A, et al. Body size and risk of colon and rectal cancer in the European Prospective Investigation Into Cancer and Nutrition (EPIC). J Natl Cancer Inst 2006 Jul 5;98(13):920-31.

(35) Palmer AJ, Roze S, Valentine WJ, Minshall ME, Foos V, Lurati FM, et al. The CORE Diabetes Model: Projecting long-term clinical outcomes, costs and cost-effectiveness of interventions in diabetes mellitus (types 1 and 2) to support clinical and reimbursement decision-making. Curr Med Res Opin 2004;20(Suppl. 1):S5-S26.

(36) Golden SH, Lazo M, Carnethon M, Bertoni AG, Schreiner PJ, Diez Roux AV, et al. Examining a bidirectional association between depressive symptoms and diabetes. JAMA 2008 Jun 18;299(23):2751-9.

(37) Whyte EM, Mulsant BH, Vanderbilt J, Dodge HH, Ganguli M. Depression after stroke: a prospective epidemiological study. J Am Geriatr Soc 2004 May;52(5):774-8.

(38) Cancer Survival in England: Patients Diagnosed, 2006–2010 and Followed up to 2011. Office of National Statistics 2012Available from: URL: <http://www.ons.gov.uk/ons/publications/re-reference-tables.html?edition=tcm%3A77-277733>

(39) Mortality Statistics: Deaths registered in England and Wales (Series DR), 2011. Office of National Statistics 2013Available from: URL: <http://www.ons.gov.uk/ons/publications/re-reference-tables.html?edition=tcm%3A77-277727>

(40) Seshasai SR, Kaptoge S, Thompson A, Di AE, Gao P, Sarwar N, et al. Diabetes mellitus, fasting glucose, and risk of cause-specific death. N Engl J Med 2011 Mar 3;364(9):829-41.

(41) Curtis L. Unit costs of health and social care. 2014.

(42) NHS reference costs 2013-14. Department of Health 2015Available from: URL: https://[www.gov.uk/government/publications/nhs-reference-costs-2013-to-2014](http://www.gov.uk/government/publications/nhs-reference-costs-2013-to-2014)

(43) British National Formulary. <http://www> bnf org/ 2015

(44) Gillett M, Chilcott J, Goyder L, Payne N, Thokala P, Freeman C, et al. Prevention of type 2 diabetes: risk identification and interventions for individuals at high risk. NICE Centre for Public Health Excellence 2011Available from: URL: <http://www.nice.org.uk/nicemedia/live/12163/57046/57046.pdf>

(45) Burr JM, Mowatt G, Hernandez R, Siddiqui MA, Cook J, Lourenco T, et al. The clinical effectiveness and cost-effectiveness of screening for open angle glaucoma: a systematic review and economic evaluation. Health Technol Assess 2007 Oct;11(41):iii-x, 1.

(46) Belsey JD, Pittard JB, Rao S, Urdahl H, Jameson K, Dixon T. Self blood glucose monitoring in type 2 diabetes. A financial impact analysis based on UK primary care. Int J Clin Pract 2009 Mar;63(3):439-48.

(47) Poole C, Tetlow T, McEwan P, Holmes P, Currie C. The prescription cost of managing people with type 1 and type 2 diabetes following initiation of treatment with either insulin glargine or insulin determir in routine general practice in the UK: a retrospective database analysis. Current Medical Research and Opinion 2007;23(1):S41-S48.

(48) Blak BT, Mullins CD, Shaya FT, Simoni-Wastila L, Cooke CE, Weir MR. Prescribing trends and drug budget impact of the ARBs in the UK. Value Health 2009 Mar;12(2):302-8.

(49) Luengo-Fernandez R, Gray AM, Rothwell PM. A population-based study of hospital care costs during 5 years after transient ischemic attack and stroke. Stroke 2012 Dec;43(12):3343-51.

(50) Alva M, Gray A, Mihaylova B, Leal J, Holman R. The impact of diabetes-related complications on healthcare costs: new results from the UKPDS (UKPDS 84). Diabetic Medicine 2014;459-66.

(51) Palmer S, Sculpher M, Philips Z, Robinsonm M., Ginnelly L, Bakhai A eal. A cost-effectiveness model comparing alternative management strategies for the use of glycoprotein IIb/IIIa antagonists in non-ST-elevation acute coronary syndrome. Report to the National Institute for Clinical Excellence.; 2008.

(52) Youman P, Wilson K, Harraf F, Kalra L. The economic burden of stroke in the United Kingdom. Pharmacoeconomics 2003;21 Suppl 1:43-50.:43-50.

(53) Baboolal K, McEwan P, Sondhi S, Spiewanowski P, Wechowski J, Wilson K. The cost of renal dialysis in a UK setting--a multicentre study. Nephrol Dial Transplant 2008 Jun;23(6):1982-9.

(54) Byrne C, Steenkamp R, Castledine C, Ansell D, Feehally J. UK Renal Registry 12th Annual Report (December 2009): chapter 4: UK ESRD prevalent rates in 2008: national and centre-specific analyses. Nephron Clin Pract 2010;115 Suppl 1:c41-67. doi: 10.1159/000301159. Epub@2010 Mar 31.:c41-c67.

(55) Cost-effectiveness of transplantation. NHS Blood and Transplant . 2013.

Ref Type: Online Source

(56) Gordois A, Scuffham P, Shearer A, Oglesby A, Tobian JA. The health care costs of diabetic peripheral neuropathy in the US. Diabetes Care 2003 Jun;26(6):1790-5.

(57) OECD. Purchasing Power Parities (PPPs) for OECD Countries. <http://stats> oecd org/Index aspx?datasetcode=SNA_TABLE4 2013Available from: URL: <http://www.oecd.org/>

(58) Madan J, Rawdin A, Stevenson M, Tappenden P. A rapid-response economic evaluation of the UK NHS Cancer Reform Strategy breast cancer screening program extension via a plausible bounds approach. Value Health 2010 Mar;13(2):215-21.

(59) Tappenden P, Eggington S, Nixon R, Chilcott J, Sakai H, Karnon J. Colorectal cancer screening options appraisal Report to the English Bowel Cancer Screening Working Group. National Health Service 2004Available from: URL: <http://www.cancerscreening.nhs.uk/bowel/scharr.pdf>

(60) The economic costs of arthritis for the UK economy. Oxford Economics 2014Available from: URL: https://[www.oxfordeconomics.com/publication/open/222531](http://www.oxfordeconomics.com/publication/open/222531)

(61) Chalder M, Wiles NJ, Campbell J, Hollinghurst SP, Searle A, Haase AM, et al. A pragmatic randomised controlled trial to evaluate the cost-effectiveness of a physical activity intervention as a treatment for depression: the treating depression with physical activity (TREAD) trial. Health Technol Assess 2012;16(10):1-iv.

(62) Curtis L. Unit costs of health and social care. 2012.

(63) Mukuria C, Rowan D, Hernandez M, Dixon S. Examining productivity losses associated with health related quality of life using patient data. Policy Research Unit in Economic Evaluation of Health and Care Interventions (EEPRU) 2014Available from: URL: <http://www.eepru.org.uk/Examining%20productivity%20losses.pdf>

(64) Squires H, Rick J, Carroll C, Hillage J. Cost-effectiveness of interventions to return employees to work following long-term sickness absence due to musculoskeletal disorders. J Public Health (Oxf) 2012 Mar;34(1):115-24.

(65) Labour Market Statistics, June 2014. Office of National Statistics 2014Available from: URL: <http://www.ons.gov.uk/ons/rel/lms/labour-market-statistics/june-2014/statistical-bulletin.html#tab-Average-Weekly-Earnings>

(66) The Cost of Brain Drain: Understanding the finanaical impact of staff turnover. Oxford Economics 2014 FebruaryAvailable from: URL: <http://www.oxfordeconomics.com/publication/open/246524>

(67) Dolan P, Gudex C, Kind P, Williams A. A social tariff for EuroQoL: Results from a UK general population survey. Discussion Paper No. 138. Centre for Health Economics 1995;University of York(York).

(68) Ara R, Wailoo A. NICE DSU Technical Support Document 12: The use of health state utility values in decision models. 2011.

(69) Alva M, Gray A, Mihaylova B, Clarke P. The Effect of Diabetes Complications on Health-Related Quality of Life: The importance of longitudinal data to address patient heterogeneity. Health Econ 2013 Jul 11;10.

(70) Coffey JT, Brandle M, Zhou H, Marriott D, Burke R, Tabaei BP, et al. Valuing health-related quality of life in diabetes. Diabetes Care 2002 Dec;25(12):2238-43.

(71) Lung TW, Hayes AJ, Hayen A, Farmer A, Clarke PM. A meta-analysis of health state valuations for people with diabetes: explaining the variation across methods and implications for economic evaluation. Qual Life Res 2011 Dec;20(10):1669-78.

(72) Peasgood T, Brazier J. Is Meta-Analysis for Utility Values Appropriate Given the Potential Impact Different Elicitation Methods Have on Values? Pharmacoeconomics 2015 Nov;33(11):1101-5.

(73) Yabroff KR, Lawrence WF, Clauser S, Davis WW, Brown ML. Burden of illness in cancer survivors: findings from a population-based national sample. J Natl Cancer Inst 2004 Sep 1;96(17):1322-30.

(74) Black C, Clar C, Henderson R, MacEachern C, McNamee P, Quayyum Z, et al. The clinical effectiveness of glucosamine and chondroitin supplements in slowing or arresting progression of osteoarthritis of the knee: a systematic review and economic evaluation. Health Technol Assess 2009 Nov;13(52):1-148.

(75) Zimovetz EA, Wolowacz SE, Classi PM, Birt J. Methodologies used in cost-effectiveness models for evaluating treatments in major depressive disorder: a systematic review. Cost Eff Resour Alloc 2012 Feb 1;10(1):1-10.

(76) Benedict A, Arellano J, De CE, Baird J. Economic evaluation of duloxetine versus serotonin selective reuptake inhibitors and venlafaxine XR in treating major depressive disorder in Scotland. J Affect Disord 2010 Jan;120(1-3):94-104.

(77) Chamnan P, Simmons RK, Forouhi NG, Luben RN, Khaw KT, Wareham NJ, et al. Incidence of type 2 diabetes using proposed HbA1c diagnostic criteria in the european prospective investigation of cancer-norfolk cohort: implications for preventive strategies. Diabetes Care 2011 Apr;34(4):950-6.

(78) NatCen Social Research. Health Survey for England. University College London Department of Epidemiology and Public Health 2003Available from: URL: <http://dx.doi.org/10.5255/UKDA-SN-5098-1>

(79) Lauritzen T, Griffin S, Borch-Johnsen K, Wareham NJ, Wolffenbuttel BH, Rutten G. The ADDITION study: proposed trial of the cost-effectiveness of an intensive multifactorial intervention on morbidity and mortality among people with Type 2 diabetes detected by screening. Int J Obes Relat Metab Disord 2000 Sep;24 Suppl 3:S6-11.

(80) Griffin SJ, Borch-Johnsen K, Davies MJ, Khunti K, Rutten GE, Sandbaek A, et al. Effect of early intensive multifactorial therapy on 5-year cardiovascular outcomes in individuals with type 2 diabetes detected by screening (ADDITION-Europe): a cluster-randomised trial. Lancet 2011 Jul 9;378(9786):156-67.

(81) Tao L, Wilson EC, Griffin SJ, Simmons RK. Performance of the UKPDS outcomes model for prediction of myocardial infarction and stroke in the ADDITION-Europe trial cohort. Value Health 2013 Sep;16(6):1074-80.

(82) Walking and cycling: local measures to promote walking and cycling as for ms of travel or recreation: Health economic and modelling report. NICE Public Health Guidance PH41 2014Available from: URL: <http://www.nice.org.uk/guidance/index.jsp?action=download&o=58985>

(83) Briggs AD, Mytton OT, Kehlbacher A, Tiffin R, Rayner M, Scarborough P. Overall and income specific effect on prevalence of overweight and obesity of 20% sugar sweetened drink tax in UK: econometric and comparative risk assessment modelling study. BMJ 2013 Oct 31;347:f6189. doi: 10.1136/bmj.f6189.:f6189.

(84) Wrigley N, Warm D, Margetts B. Deprivation, diet, and food-retail access: findings from the Leeds 'food deserts' study. Environment and Planning 2003;35(1):151-88.

(85) Sargeant LA, Wareham NJ, Bingham S, Day NE, Luben RN, Oakes S, et al. Vitamin C and Hyperglycemia in the European Prospective Investigation into Cancer-Norfolk (EPIC-Norfolk) Study. Diabetes care 2000;23(6):726-32.

(86) John JH, Ziebland S, Yudkin P, Roe LS, Neil HA. Effects of fruit and vegetable consumption on plasma antioxidant concentrations and blood pressure: a randomised controlled trial. Lancet 2002 Jun 8;359(9322):1969-74.

(87) Holdsworth M, Raymond NT, Haslam C. Does the Heartbeat Award scheme in England result in change in dietary behaviour in the workplace? Health Promot Int 2004 Jun;19(2):197-204.

(88) Family Food 2012. Defra 2012Available from: URL: https://[www.gov.uk/government/publications/family-food-2012](http://www.gov.uk/government/publications/family-food-2012)

(89) The nutritional composition of dairy products. The Dairy Council 2014Available from: URL: <http://www.milk.co.uk/page.aspx?intPageID=197>

(90) Harding AH, Sargeant LA, Welch A, Oakes S, Luben RN, Bingham S, et al. Fat consumption and HbA(1c) levels: the EPIC-Norfolk study. Diabetes Care 2001 Nov;24(11):1911-6.

(91) Gray C, Anderson A, Clarke A, Dalziel A, Hunt K, Leishman J, et al. Addressing male obesity: an evaluation of a group-based weight management intervention for Scottish men. Journal of Mens Health 2009;6(1):70-81.

(92) McKellar G, Morrison E, McEntegart A, Hampson R, Tierney A, Mackle G, et al. A pilot study of a Mediterranean-type diet intervention in female patients with rheumatoid arthritis living in areas of social deprivation in Glasgow. Ann Rheum Dis 2007 Sep;66(9):1239-43.

(93) Wrieden WL, Anderson AS, Longbottom PJ, Valentine K, Stead M, Caraher M, et al. The impact of a community-based food skills intervention on cooking confidence, food preparation methods and dietary choices - an exploratory trial. Public Health Nutr 2007 Feb;10(2):203-11.

(94) Dunkley AJ, Bodicoat DH, Greaves CJ, Russell C, Yates T, Davies MJ, et al. Diabetes Prevention in the Real World: Effectiveness of Pragmatic Lifestyle Interventions for the Prevention of Type 2 Diabetes and of the Impact of Adherence to Guideline Recommendations: A Systematic Review and Meta-analysis. Diabetes Care 2014 Apr;37(4):922-33.

(95) Cochrane T, Gidlow CJ, Kumar J, Mawby Y, Iqbal Z, Chambers RM. Cross-sectional review of the response and treatment uptake from the NHS Health Checks programme in Stoke on Trent. J Public Health (Oxf) 2013 Mar;35(1):92-8.

(96) Gray LJ, Davies MJ, Hiles S, Taub NA, Webb DR, Srinivasan BT, et al. Detection of impaired glucose regulation and/or type 2 diabetes mellitus, using primary care electronic data, in a multiethnic UK community setting. Diabetologia 2012 Apr;55(4):959-66.

(97) Thomas C, Sadler S, Squires H, Gillett M, Brennan A. Assessing the potential return on investment of the proposed NHS diabetes prevention programme in different population subgroups. Public Health England 2016Available from: URL: <http://www.yhpho.org.uk/default.aspx?RID=235836>

(98) Clarke P, Gray A, Legood R, Briggs A, Holman R. The impact of diabetes-related complications on healthcare costs: results from the United Kingdom Prospective Diabetes Study (UKPDS Study No. 65). Diabet Med 2003 Jun;20(6):442-50.

(99) Tabak AG, Jokela M, Akbaraly TN, Brunner EJ, Kivimaki M, Witte DR. Trajectories of glycaemia, insulin sensitivity, and insulin secretion before diagnosis of type 2 diabetes: an analysis from the Whitehall II study. Lancet 2009 Jun 27;373(9682):2215-21.

(100) Avery L, Flynn D, van Wersch A, Sniehotta FF, Trenell MI. Changing Physical Activity Behaviour in Type 2 Diabetes. Diabetes care 2012;35:2681-9.

(101) Post RE, Mainous III AG, King DE, Simpson KN. Dietary fiber for the treatment of type 2 diabetes mellitus: A meta-analysis. JABFM 2012;25(1):16-23.

1. The model did not converge when BMI slope was included as a predictor for HDL growth. [↑](#footnote-ref-1)
2. 46% of individuals switch to the new store with an incremental change in fruit and veg of 0.252. 7.8% switch from a budget store with an additional incremental change of 0.595. [↑](#footnote-ref-2)
